# Design of a proteolytic module for improved metabolic modeling of *Bacteroides caccae*

**DOI:** 10.1128/msystems.00153-24

**Published:** 2024-03-22

**Authors:** Amandine Paulay, Ghjuvan M. Grimaud, Raphaël Caballero, Béatrice Laroche, Marion Leclerc, Simon Labarthe, Emmanuelle Maguin

**Affiliations:** 1 Université Paris-Saclay, INRAE, AgroParisTech, UMR1319 Micalis Institute, Jouy-en-Josas, France; 2 Biomathematica, Ajaccio, France; 3 Université Paris-Saclay, INRAE, MaIAGE, Jouy-en-Josas, France; 4 Université Paris-Saclay, Inria, Centre Inria de Saclay, Palaiseau, France; 5 Pendulum Therapeutics, San Francisco, California, USA; 6 University of Bordeaux, INRAE, BIOGECO, Cestas, France; 7 Inria, Univ. Bordeaux, INRAE, Talence, France; Argonne National Laboratory, Lemont, Illinois, USA

**Keywords:** microbiota, flux balance analysis, proteolysis, proteases, metabolic modeling, holobiont

## Abstract

The gut microbiota plays a crucial role in health and is significantly modulated by human diets. In addition to Western diets which are rich in proteins, high-protein diets are used for specific populations or indications, mainly weight loss. In this study, we investigated the effect of protein supplementation on *Bacteroides caccae*, a Gram-negative gut symbiont. The supplementation with whey proteins led to a significant increase in growth rate, final biomass, and short-chain fatty acids production. A comprehensive genomic analysis revealed that *B. caccae* possesses a set of 156 proteases with putative intracellular and extracellular localization and allowed to identify amino acid transporters and metabolic pathways. We developed a fully curated genome-scale metabolic model of *B. caccae* that incorporated its proteolytic activity and simulated its growth and production of fermentation-related metabolites in response to the different growth media. We validated the model by comparing the predicted phenotype to experimental data. The model accurately predicted *B. caccae*’s growth and metabolite production (*R*^2^ = 0.92 for the training set and *R*^2^ = 0.89 for the validation set). We found that accounting for both ATP consumption related to proteolysis, and whey protein accessibility is necessary for accurate predictions of metabolites production. These results provide insights into *B. caccae*’s adaptation to a high-protein diet and its ability to utilize proteins as a source of nutrition. The proposed model provides a useful tool for understanding the feeding mechanism of *B. caccae* in the gut microbiome.

**IMPORTANCE:**

Microbial proteolysis is understudied despite the availability of dietary proteins for the gut microbiota. Here, the proteolytic potential of the gut symbiont *Bacteroides caccae* was analyzed for the first time using pan-genomics. This sketches a well-equipped bacteria for protein breakdown, capable of producing 156 different proteases with a broad spectrum of cleavage targets. This functional potential was confirmed by the enhancement of growth and metabolic activities at high protein levels. Proteolysis was included in a *B. caccae* metabolic model which was fitted with the experiments and validated on external data. This model pinpoints the links between protein availability and short-chain fatty acids production, and the importance for *B. caccae* to gain access to glutamate and asparagine to promote growth. This integrated approach can be generalized to other symbionts and upscaled to complex microbiota to get insights into the ecological impact of proteins on the gut microbiota.

## INTRODUCTION

The gut microbiota, i.e., the complex microbial community living within the intestinal tract in symbiosis with its host, is now recognized as an essential contributor to human health and well-being. High throughput omics data have increasingly revealed mechanisms of interaction between the human host and its commensal microbes (1). The microbiota degrades food residues and produces many metabolites and compounds that are uptaken by the host, such as short-chain fatty acids (SCFAs), amino acids, and essential vitamins (2). The microbiota is also involved in regulation mechanisms at the whole-body scale, such as immune system maturation, intestinal mucosa turnover, or the production of neurotransmitters (3, 4). It is well established that the host diet is one of the important factors that shape the microbiota composition and function, which impacts the interactions with the host (5, 6).

In Western countries, the last decades have been characterized by an increase in dietary protein consumption. Compared to current dietary recommendations (7), the Western diet contains higher levels of fat and proteins and a lower level of fiber (8). It is associated with metabolic disorders such as obesity, type 2 diabetes, and liver diseases (9). Dietary proteins undergo processing in the upper gastrointestinal (GI) tract through the action of proteases and peptidases that are secreted by the human stomach and small intestine. This process leads to the formation of peptides and amino acids, which are primarily absorbed in the small intestine. Nevertheless, in a normal diet, up to 10% of the dietary proteins reach the large intestine, where the microbiota can break them down (10). Nowadays, some diets tend to increase the proportion of proteins in the colon. High-protein diets (HPDs) are recommended for athletes to favor muscle regeneration, for elderly people in an attempt to limit sarcopenia (11, 12)and for obese patients and the general population, as an alternative to calorie restriction for weight loss (13). In addition, as plant proteins are less digestible than animal proteins (14), the trend to re-vegetate diets to limit the drawbacks and environmental impact of Western diets could also result in higher protein levels in the colon. This leads to an expansion of the nutritional niche for proteolytic microorganisms, such as the *Bacteroides* species (5). Of note, increased fecal proteolytic activity is associated with several diseases such as inflammatory bowel diseases (15, 16). Bacterial protein degradation through proteolysis ultimately results in amino acids that can be used to synthesize new proteins or various metabolites such as short-chain fatty acids (SCFAs) or branched-chain fatty acids (BCFAs) which can be energy sources for enterocytes (17) but which are also considered as mediators targeting the host. A number of other amino acid -derived microbial metabolites were also identified as mediators of the microbiota-host cross-talk with positive or detrimental effects (18).

In the last decade, several mathematical methods were developed to study the bacterial metabolism in various environments (19). Among them, genome-scale metabolic reconstructions (GENREs) were based on the core metabolism of whole genomes and generated using the annotated genome of given microorganisms (20–22). Semi-automatic reconstruction allowed the generation of hundreds of GENREs (23), especially among the gut microbiota species (24). On top of GENREs, metabolic fluxes can be predicted across the metabolic network using constraint-based models; the most used one being flux balance analysis (FBA). FBA estimates a flux distribution giving the maximum possible growth rate achievable by a microorganism on defined media usually composed of simple sugars, amino acids, and minerals. However, protein breakdown is not included in constraint-based models, in which dietary proteins are represented by free amino acids.

In this work, we investigated whether proteolysis affected both the growth and metabolism of *Bacteroides caccae* ATCC 43185, a Gram-negative gut symbiont from the abundant *Bacteroidaceae* family, and how to plug this enzymatic process in a curated genome-scale metabolic model that integrated a comprehensive analysis of the species genomes and experimental results. For these purposes, we performed growth experiments with and without whey protein supplementation, analyzed *in silico* the proteolytic potential of 47 *B. caccae* strains, and designed proteolytic modules that were plugged in a *B. caccae* curated metabolic model. We finally used a dynamic flux balance analysis (dFBA) algorithm including several metabolite intake regulations, fitted, and validated on independent data sets, to investigate the dynamic effects of extracellular protein availability on *B. caccae* metabolism.

## RESULTS

### Whey protein supplementation enhanced *B. caccae* metabolism

**Fig 1:**
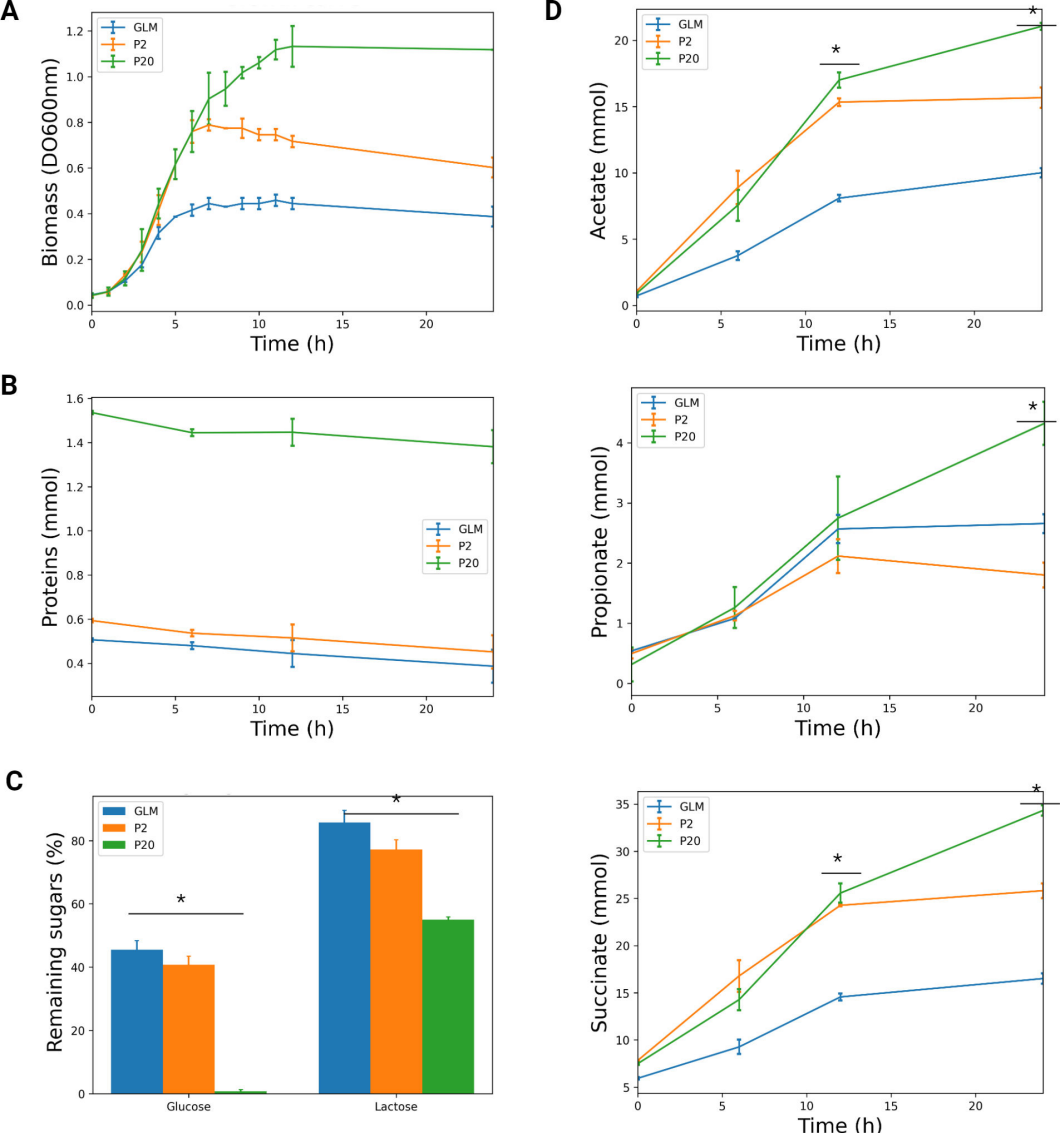
*B. caccae* growth in GLM without and with whey protein supplementation. (**A**) Growth curve of *B. caccae* ATCC 43185 in the GLM, P2 and P20 during 24 h of incubation at 37°C in anaerobiosis. (**B**) Total protein concentrations in culture supernatants at the time of inoculation (T0) and after 6, 12, and 24 h of incubation. (**C**) The percentage of D-glucose and D-lactose remaining in each medium at 24 h after inoculation relative to the initial value at T0. (**D**) The concentrations of acetate, propionate, and succinate at 0, 6, 12, and 24 h. Each value is the mean ± SD from three independent experiments. For each time point, a Kruskal-Wallis test was performed to compare the concentrations of each metabolite across the three media. *, *P* value < 0.05.

To investigate the ability of *B. caccae* ATCC 43185 to metabolize proteins, we used a semi-defined medium called Glucose-Limited Medium (GLM) developed by Jaoui et al. (25), which was supplemented by 2 or 20 g L^−1^ of whey protein leading to the P2 and P20 media, respectively. Note that for technical reasons, we filtered the media leading to a slight decrease of protein concentrations (M&M). The growth kinetics of *B. caccae*, the consumption of carbon sources and proteins, and the production of acetate, propionate, and succinate were monitored in the three media at different time points during 24 h of incubation at 37°C in anaerobiosis (Fig. 1).

Whey proteins increased the bacterial growth rate by 23.2% (±3.9%) both in P2 and P20 compared to the GLM (Fig. 1A). The final biomass also increased but differently in P2 and P20 with significant differences between the three growth conditions after 7 h of incubation and beyond (*P* value < 0.05, one-way ANOVA on ranks). The final biomass was higher in P2 and P20 compared to GLM (*P* value = 3·10^−5^, *t* test) and significantly higher in P20 than in P2 (*P* value = 1·10^−3^, *t* test).

The concentrations of several metabolites were measured during the 24 h of growth to investigate the impact of the protein supplementation on the strain metabolism. As expected, the initial glucose concentration was similar in the three media, while the lactose concentration slightly varied with the whey protein supplementation (M&M). However, glucose remained the main sugar in the media (5.13 ± 0.5 g L^−1^ of glucose vs 0.36 ± 0.005 g L^−1^ of lactose in P20 medium). At 24 h, the glucose was not limiting in GLM and P2, while it was fully consumed in P20 (Fig. 1C). The consumption rate of proteins was similar in the three media although slightly increased in P20 compared to GLM (Fig. 1B). The measures of free amino acids at 0, 6, and 12 h of growth revealed an increase in glutamate, glycine, alanine, proline, and valine overtime in P2 and P20 compared to GLM and an upward trend for alanine, arginine, histidine, isoleucine, leucine, and lysine (Table S1). These observations corroborated that whey proteins were indeed catabolized and contributed to the changes in amino acids concentrations. The aspartate and cysteine were slightly more consumed in P2 and P20 than in GLM and cysteine was almost fully consumed in P20 (96.4% consumed compared to 87.5% in P2 and 71.4% in GLM).

In P20, the production of acetate, propionate, and succinate increased: 57.9% for acetate, 62% for succinate (*P* value < 10^−3^ for each, *t* test) and 55.9% for propionate (*P* value < 10^−5^, *t* test) compared to the values in GLM. In P2, the acetate and succinate productions also increased by 17.5% and 17.3% (*P* value ≤ 3·10^−4^, *t* test), respectively, while the amount of propionate decreased by 32.2% compared to GLM (*P* value < 0.05, *t* test) (Fig. 1D).

**Fig 2:**
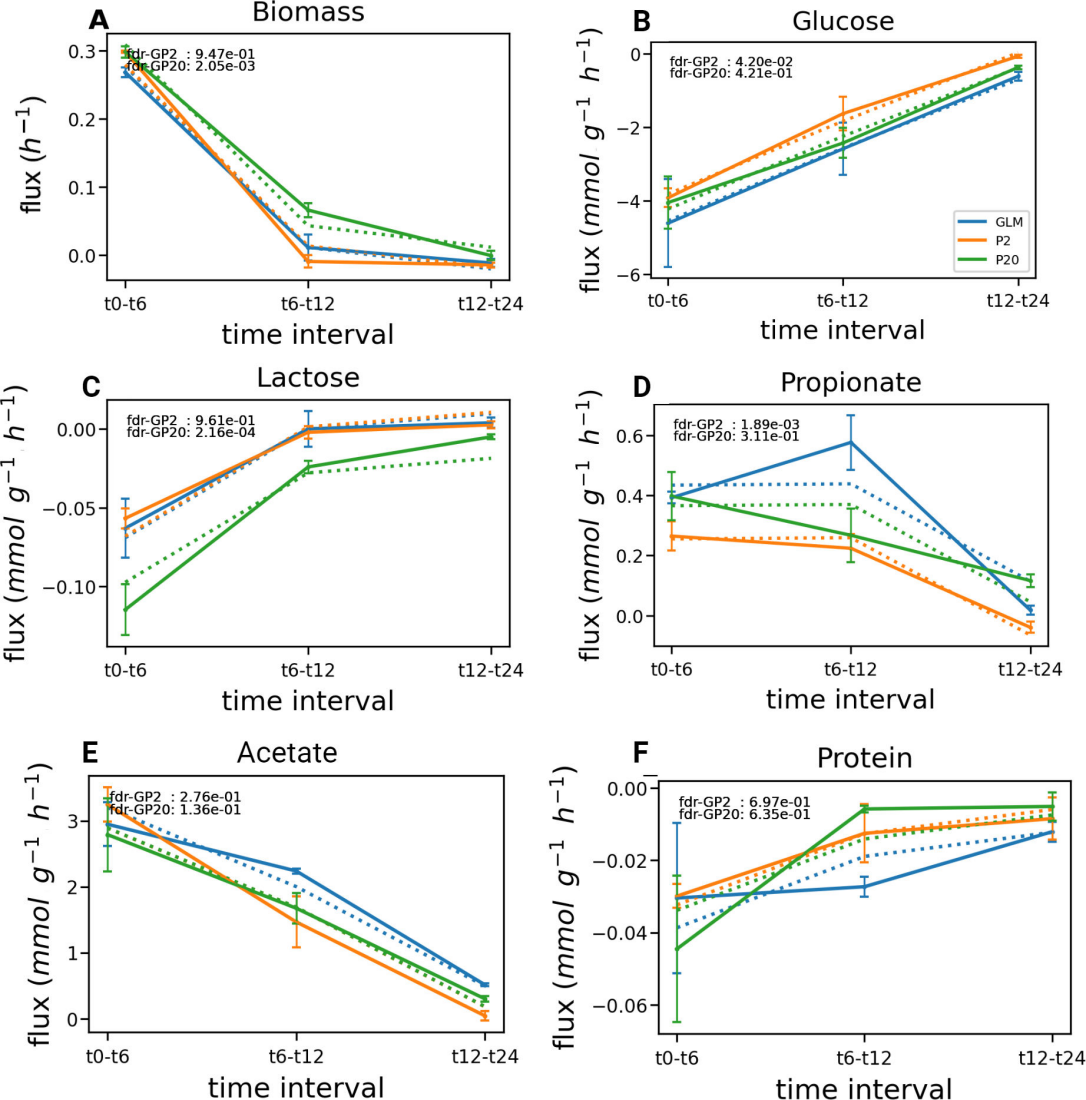
Flux differences between GLM, P2, and P20. (**A**) Biomass. (**B**) Glucose flux. (**C**) Lactate flux. (**D**) Propionate flux. The mean fluxes (plain lines) between two time points (0–6, 6–12, and 12–24 h) were calculated from the metabolite concentrations measured in three independent experiments for each medium. The estimated fluxes (dotted lines) were produced using a mixed model (M&M). Significant differences after multiple test corrections correspond to an FDR < 0.05. Positive and negative fluxes indicated metabolite production and consumption, respectively. Blue, GLM; orange, P2; and green, P20. The error bars represent the SD.

We evaluated the production and consumption fluxes (M&M) for each metabolite between two consecutive time points (Fig. 2). Overall, unlike final concentrations, the computed fluxes for production and consumption were mostly similar in the P2, P20, and GLM cultures with few differences concerning sugars consumption and lactate and propionate productions. No significant changes in protein consumption rates were observed in P2 and P20 compared to GLM despite enhanced protein availability (Fig. 2F), unlike biomass production which was significantly increased in P20 (Fig. 2A). Lactose consumption flux was higher in P20 but not in P2 (*P* value = 2.6·10^−4^, *t* test, BH correction Fig. 2C), while glucose flux (Fig. 2B) was reduced in P2 (*P* value = 4·10^−3^, *t* test, BH correction) compared to GLM. Of note, the lactate fluxes in P2 and P20 were slightly lower than in GLM (*P* value ≤ 4·10^−2^, *t* test, BH correction). Finally, the flux of propionate production is significantly decreased in P2 compared to GLM (Fig. 2D*, P* value = 1.89·10^−3^, *t* test, BH correction), while acetate production fluxes are similar in all three media (Fig. 2E). Altogether, these observations suggested that the differences observed in final metabolites concentrations between GLM, P2, and P20 (Fig. 1) were mostly due to an increased growth rate on protein-enriched media, rather than important changes in per-capita fluxes.

### Genome analysis for protein and amino acids metabolism

#### Proteases

**Fig 3:**
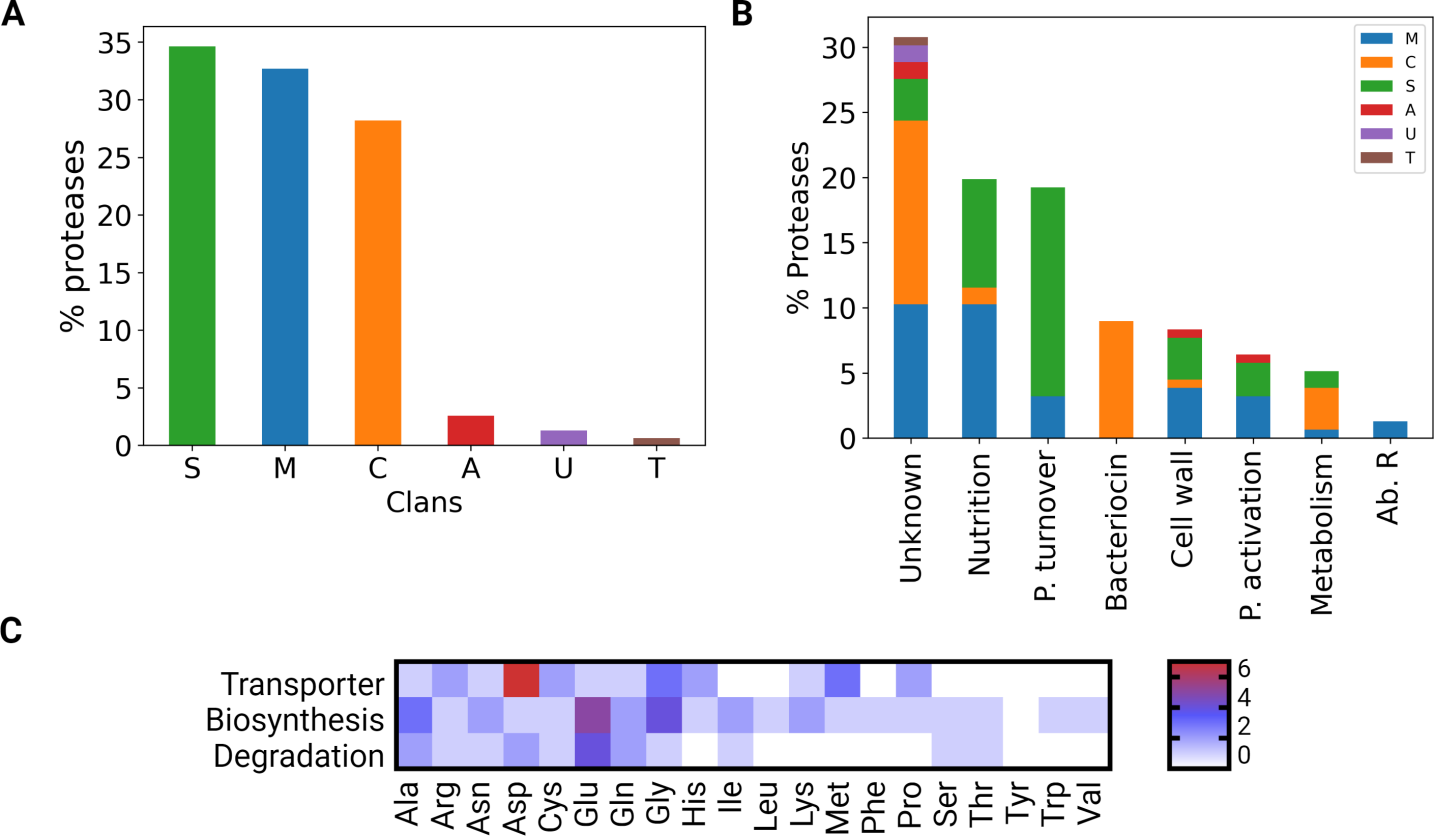
Features of the *B. caccae* ATCC 43185 proteases. (**A**) Distribution of the 156 predicted proteases within five clans: A, aspartate protease; C, cysteine protease; M, metalloprotease; S, serine protease; T, threonine protease; and U, unknown protease. (**B**) Functional categories for the 156 predicted proteases with clans displayed according to the figure color code. (**C**) Number of amino acid transporters, biosynthetic and degradative pathways identified with GAPSEQ and manually confirmed. P., protein and Ab. R, antibiotic resistance.

We retrieved 156 putative non-redundant proteases from a thorough analysis of the *B. caccae* ATCC 43185 reference genome (Table S2). Fifty-one of these putative proteases presented a signal peptide suggesting an extracellular localization either in the periplasm or outside the bacterial cell. The proteases can be classified in clans according to their catalytic sites and each clan can be subdivided into families based on the similarity of amino acid sequences (26). The ATCC 43185 proteases were mainly serine-proteases (54 proteases belonging to 15 families), metalloproteases (51 proteases distributed in 20 families), and cysteine proteases (44 proteases grouped in 11 families) (Fig. 3A). The four aspartate proteases grouped in a unique family, only one threonine protease was found and two putative proteases belonged to the unknown category. Among these 156 putative proteases, 48.7% (77) were unassigned meaning that no holotype was identified and 16% (25) were considered as non-peptidase homologs as one or more of the expected catalytic residues were lacking.

To elucidate the functional implications of proteases identified in the *B. caccae* genome, we devised a classification scheme based on the well-established MEROPS protease database (27) (Fig. 3B). Each identified protease was linked to its corresponding family within the database. From this approach, we distilled seven broad functional categories that provided a framework for understanding protease roles within the bacterium. The categories included Nutrition, Protein Turnover, Bacteriocin, Cell Wall, Protein Activation, Metabolism, and Antibiotic Resistance, and an “Unknown” category for proteases with unclear function. Besides the group of proteases of unknown function, the most represented categories in *B. caccae* were (i) the protein turnover possibly involved in quality control through the degradation of intracellular, misfolded, and regulated proteins with 30 proteins and (ii) the nutrition which included 31 proteins likely to be involved in bacterial feeding. Then four functional categories were less represented with 16 cysteine proteases grouped in the bacteriocin and bacteriocin-processing category grouping proteases that inhibit the growth or the extracellular bacteriocins from other bacteria, 13 were in the cell wall category comprising proteases involved in the metabolism of cell walls, 10 belonged to the protein activation category which remove the initiating methionine of proteins, and 8 assigned to the general metabolism group composed of various metabolic proteases. The antibiotic resistance cluster was the least represented with only two metalloproteases that may degrade antibiotics.

Thirty-one proteases were assigned to the nutrition category: 16 are metalloproteases, 13 serine proteases and 2 cysteine proteases, respectively. A signal peptide was detected in 13 proteases and only one in the remaining 18 presented a transmembrane domain. Altogether, these proteases present a diversity of substrates confirming that *B. caccae* is well-equipped for proteolysis (Table 1).

**TABLE 1:** Features of the proteases belonging to the nutrition category

Sub family	Homolog	Homolog origin	Putative peptide substrates	Localisation	Pan-genome
M03.005	Peptidyl-dipeptidase Dcp	*Chitinophaga pinensis*	Tripeptides and longer	EC	Core
S46.002	Dipeptidyl-peptidase 11	*Emericella nidulans*	Dipeptidyl-peptidase	EC	Core
S46.002	Dipeptidyl-peptidase 11	*B. thetaiotaomicron*	Dipeptidyl-peptidase	EC	Core
S46.001	Dipeptidyl-peptidase 7	*B. thetaiotaomicron*	Dipeptidyl-peptidase	EC	Core
C11.006	*NaN*	*B. thetaiotaomicron*	Unknown	EC	Cloud
C11.005	Thetapain	*Bacteroides fragilis*	Unknown	EC	Shell
S08.UPA	Unassigned	*B. thetaiotaomicron*	Gly-Gly endopeptidase	EC	Cloud
S08.UPA	Unassigned	*B. thetaiotaomicron*	Gly-Gly endopeptidase	EC	Core
S33.UPW	Unassigned	*B. thetaiotaomicron*	Prolyl aminopeptidase	EC	Shell
M13.UPW	Unassigned	*E. faecium*	Unknown	EC	Core
M23.UPB	Unassigned	*B. thetaiotaomicron*	Unknown	EC	Cloud
M23.UPB	Unassigned	*B. thetaiotaomicron*	Unknown	EC	Cloud
M20.UNB	Non-peptidase homolog	*B. thetaiotaomicron*	Tripeptides	EC	Cloud
M23.UPB	Unassigned	*Polaromonas* sp. JS666	Unknown	M	Shell
M03.005	Peptidyl-dipeptidase Dcp	*B. thetaiotaomicron*	Tripeptides and longer	IC	Core
M20.003	Peptidase T	*B. thetaiotaomicron*	Tripeptides	IC	Core
M20.012	Pep581 peptidase	*B. thetaiotaomicron*	Dipeptides and longer	IC	Core
M20.012	Pep581 peptidase	*B. thetaiotaomicron*	Dipeptides and longer	IC	Core
S33.990	Haloalkane dehalogenase	*B. thetaiotaomicron*	Prolyl aminopeptidase	IC	Core
M20.016	Putative peptidase	*E. faecium*	Dipeptides	IC	Core
S33.UNE	Non-peptidase homolog	*S. viridis*	Prolyl aminopeptidase	IC	Core
S15.UPW	Unassigned	*B. thetaiotaomicron*	X-Pro dipeptidyl-peptidases	IC	Core
S15.UPW	Unassigned	*B. thetaiotaomicron*	X-Pro dipeptidyl-peptidases	IC	Core
S33.UPW	Unassigned	*Emericella nidulans*	Prolyl aminopeptidase	IC	Core
S33.UPW	Unassigned	*Salinibacter ruber*	Prolyl aminopeptidase	IC	Core
S33.UPW	Unassigned	*T. erythraeum*	Prolyl aminopeptidase	IC	Core
M20.UPD	Unassigned	*O. anthropi*	Unknown	IC	Shell
M20.UPD	Unassigned	*S. viridis*	Unknown	IC	Core
M20.UPF	Unassigned	*S. viridis*	Unknown	IC	Core
M23.UPB	Unassigned	*B. thetaiotaomicron*	Unknown	IC	Cloud
M23.UPB	Unassigned	*B. thetaiotaomicron*	Unknown	IC	Core

#### Amino acids

The amino acids transporters, biosynthesis, and degradation pathways were also searched in the *B. caccae* ATCC 11458 genome. At least one transporter was identified for 12 amino acids (Ala, Arg, Asp, Asn, Cys, Glu, Gln, Gly, His, Lys, Met, and Pro) (Fig. 3C). Biosynthesis pathways were detected for all amino acids except tyrosine. It is noteworthy that five and four biosynthesis pathways were identified for glutamate and glycine, respectively. The absence of transporter and biosynthesis pathway for tyrosine suggests that *B. caccae* obtains its supply via intracellular degradation of peptides.

### The proteolytic activity of *B. caccae* is conserved within the species

In order to assess the conservation of genes coding for proteases between strains of this species, we established a species pan-genome using 47 *B. caccae* genomes downloaded from the NCBI database (28) (Table S3). These genomes represented 13,661 gene families. Based on our analysis, the core genome included 2636 (19.3%) gene families (present in at least 99% of the genomes), while the soft-core contained 135 genes (common to 95–99% of the genomes). The rest of the pan-genome is composed of 20.6% of shell genes (shared by 15–95% of the genomes), and 59.1% of cloud genes (shared by less than 15% of the genomes). According to our analysis, the pan-genome is open and the total gene repertoire could increase by adding newly sequenced genomes (Fig. S1). This pan-genome characterization allowed the assessment of the conservation of proteases: 56% (88) of the proteases belonged to the core genome (Table S2, column T) including 21 out of 31 proteases with a predicted role in nutrition (Table 1). Considering the conservation within the species strains of the proteases with a nutritional function, the design of a proteolytic reaction to be added in the *B. caccae* metabolic model was rational.

### Refinement of *B. caccae* metabolic model

The *B. caccae* metabolic model retrieved from the AGORA database (23) included 730 genes, 952 metabolites, and 1,225 reactions. We refined this model with the amino acids pathways and transporters detected in the ATCC 11453 genome; overall 35 reactions were added (Table S4). Briefly, the presence of uptake reaction for every amino acid present in the media was ensured and transporter types that were not considered in the AGORA model were added (M&M for details).

**Fig 4:**
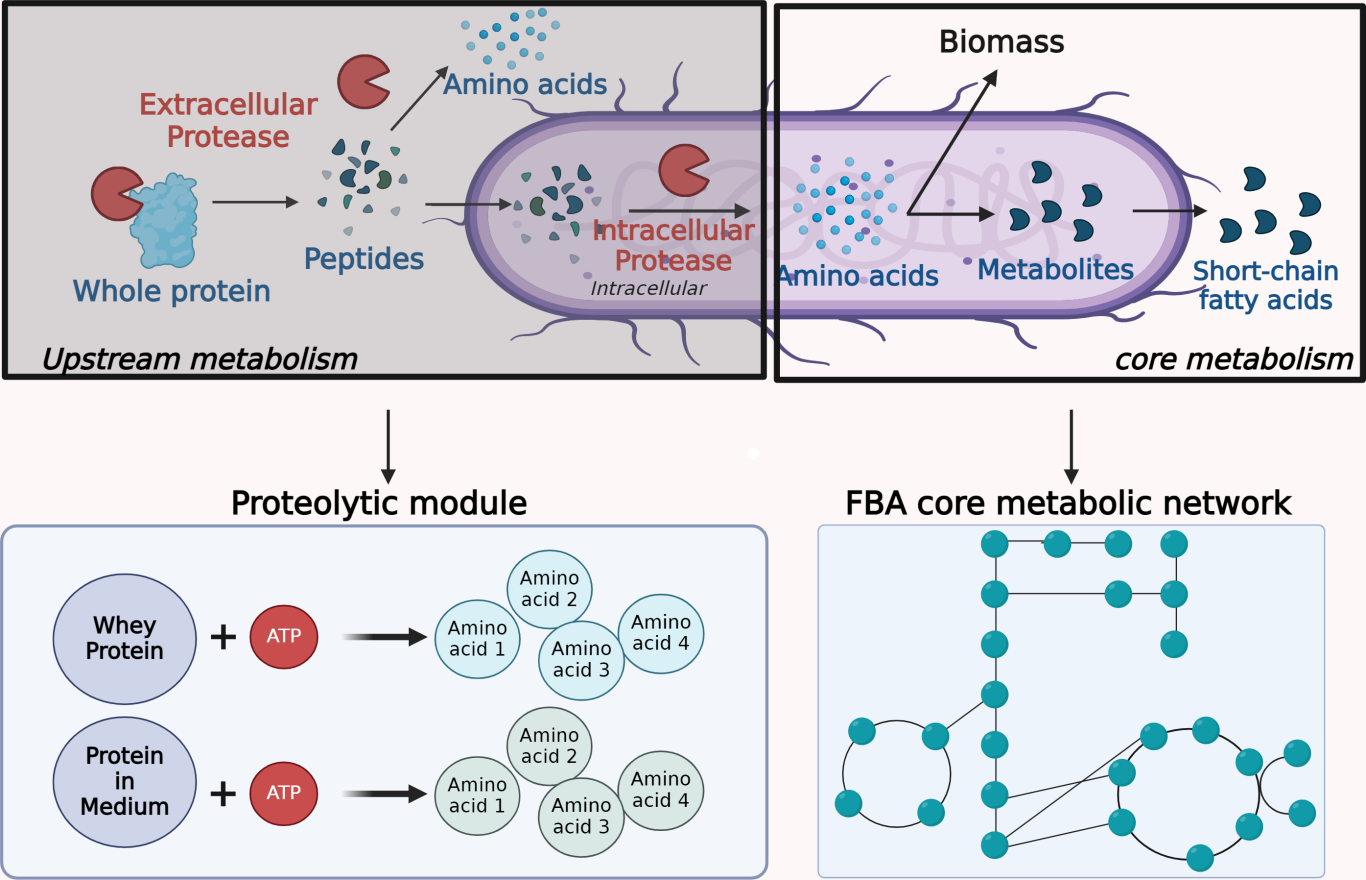
Schematic diagram of the core metabolism included in the FBA metabolic model (right panels) and the upstream metabolism of complex compounds such as proteins which are most often not included (left panels). During proteolysis, the proteins are broken down into peptides and amino acids which are transported in the bacteria and become available for the core metabolism. In this work, proteolysis was modeled with two reactions (lower left panel), one degrading the whey proteins in amino acids and the other one degrading the other type of proteins present in the medium. The production of proteases is taken into account via ATP consumption. The resulting amino acids are entering the FBA core metabolism (right lower panel).

Standard FBA metabolic models consider the intracellular core metabolic network (29), i.e., well-characterized metabolic reactions involving well-annotated enzymes. Typical FBA frameworks do not consider proteins that are directly described as free amino acids (Fig. 4). The complexity and variability of proteolysis explain why it is not included in FBA. First, the proteases and peptidases are barely characterized for numerous microorganisms and the prediction of their functions and cleavage specificities are challenging. Second, modeling proteolysis as a succession of unitary reactions is arduous due to the diversity of protein substrates and of their degradation products. Third it is challenging to model the multiplicity of proteins in a diet.

We designed two proteolytic modules based on a pseudo chemical reaction (M&M, equation 3) summarizing the breakdown of proteins into extracellular amino acids, that we included in the *B. caccae* FBA model (Fig. 4), one modeling the degradation of whey proteins for the P2 and P20 media, and the second one for general proteins included in GLM, hence present in the three media. The stoichiometric coefficients of the reaction accounted for the amino acid composition of the proteins, and for the energetic cost of protease synthesis, sum up as an ATP cost (M&M).

To capture the dynamics of the metabolism in proteolytic conditions, we included the FBA model in a dFBA model, which allows for the simulation of time-dependent changes in the extracellular environment and in the metabolism of microorganisms (30, 31). The experimental results suggested that some metabolites were under specific regulation. We therefore added Monod or Comtois kinetics on glucose, protein, and lactose uptake in the dFBA simulation (M&M, equations 4 and 5): with these regulations, the simulations were consistent with the experiments.

The experimental data did not allow the identification of the phenomenon responsible for the entry in the stationary phase. We then added a multiplicative carrying capacity that depended on the whey protein level in the media at T0 (MM, equation 4), and that progressively took over the FBA biomass prediction when the population levels rose towards the carrying capacity. However, the dFBA model kept predicting the metabolite inputs and outputs independently to this carrying capacity. We limited the number of inferred parameters to 5 to avoid over-fitting (list in M&M). GLM and P20 experimental data were used as training sets to calibrate the model through parameter inference, while P2 was used as a validating data set.

The experimental data did not allow the identification of the phenomenon responsible for the entry in the stationary phase. We then added a multiplicative carrying capacity that depended on the whey protein level in the media at T0 (M&M, equation 4), and that progressively took over the FBA biomass prediction when the population levels rose towards the carrying capacity. However, the dFBA model kept predicting the metabolite inputs and outputs independently to this carrying capacity. We limited the number of inferred parameters to 5 to avoid over-fitting (list in M&M). GLM and P20 experimental data were used as training sets to calibrate the model through parameter inference, while P2 was used as a validating data set. To guarantee the highest level of accuracy and reliability in our metabolic modeling, we employed MEMOTE, a comprehensive and standardized tool for metabolic model validation (32).

In the area of metabolite annotations, our model demonstrated a complete presence of annotations, achieving a 100% score. However, scores varied across different databases, culminating in a cumulative score of 55% (Fig. S5). This variation underscores the diverse nature of the databases and the challenges in aligning them. Regarding reaction annotations, our model continued to show its robustness, displaying a 100% presence of reaction annotations. The aggregate score across all databases reached 57%. In addition, our model achieved a 100% in System Biology Ontology (SBO) Terms Annotation.Collectively, these metrics from the MEMOTE analysis resulted in an overall score of 84% for our model, which is in the range of the MEMOTE score of the AGORA2 ATCC 43185 (83%), and outperforms the AGORA1 version (45%) (Fig. S5)

### *B. caccae* model can accurately predict biomass production and metabolites dynamics

In the three media, the model predicted that glucose, lactose, proteins, and various amino acids were consumed, while propionate, acetate, succinate, formate, and putrescine were produced, thus correctly reproducing the global functioning of *B. caccae*.

**Fig 5:**
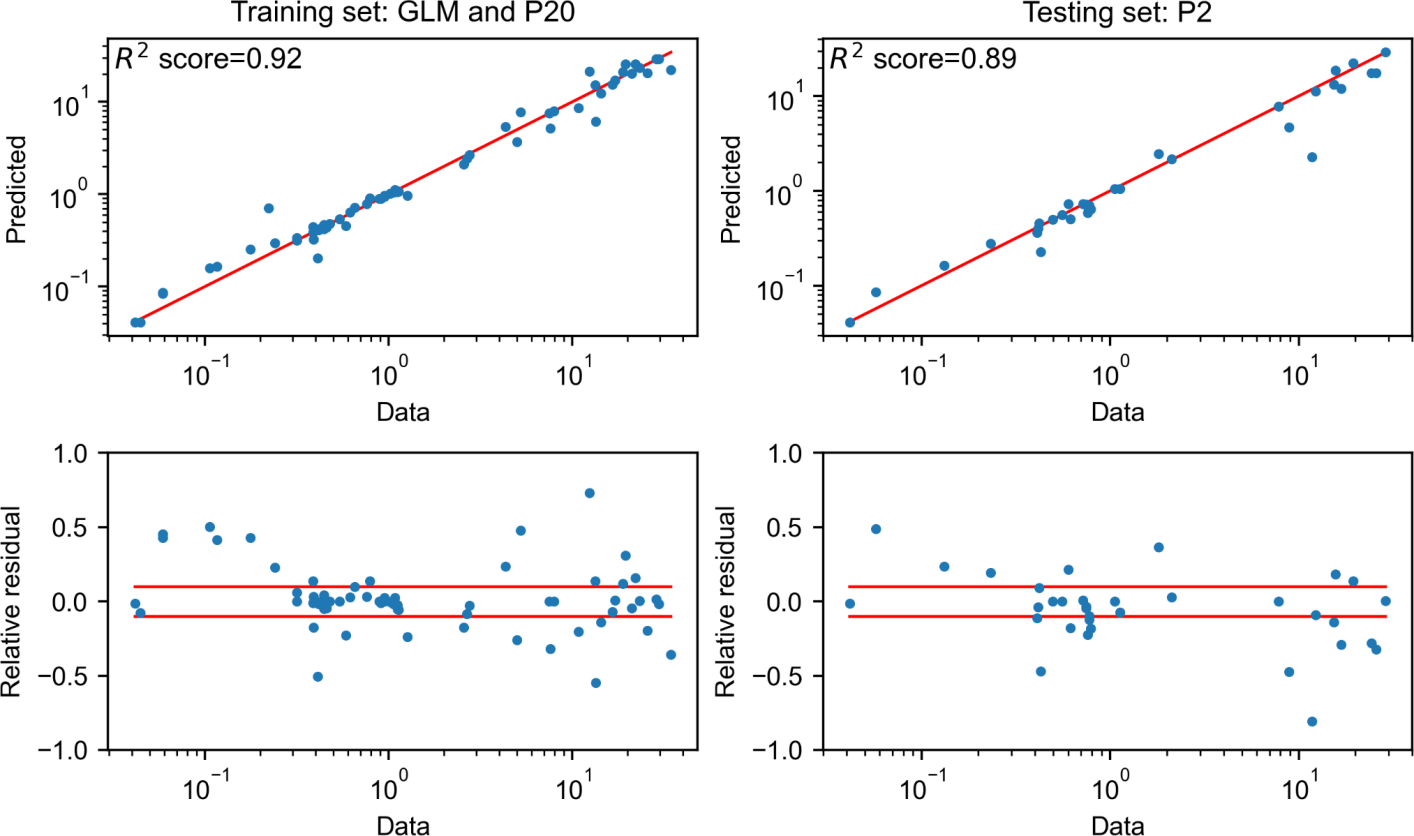
Goodness of fit of the model. Goodness of fit for the GLM and P20 media (left panel) and P2 medium (right panel), pooling six observed concentrations, i.e., biomass, glucose, lactose, propionate, acetate, and succinate. Each panel includes two subplots. The top subplot displays the experimental values versus the corresponding predicted values computed with the model in log-log scale, together with the bisector (red line). The whole data set is pooled in this graph (time point and data fields). The corresponding coefficient of determination *R*^2^ is indicated (top). The bottom subplot displays for each model prediction the corresponding relative residual error, and the horizontal red dotted lines represent a 10% error: a large majority of points are kept in the range of ±10% error.

To quantitatively assess the goodness of fit, we displayed the model prediction against their corresponding experimental values in the training and validation sets (Fig. 5). In the training set, a good correspondence between model prediction and data were observed (*R*^2^ = 0.93), validating the parameter inference. In the validation set, a similar accuracy was kept (*R*^2^ = 0.89), which validated the prediction capability of the model.

**Fig 6:**
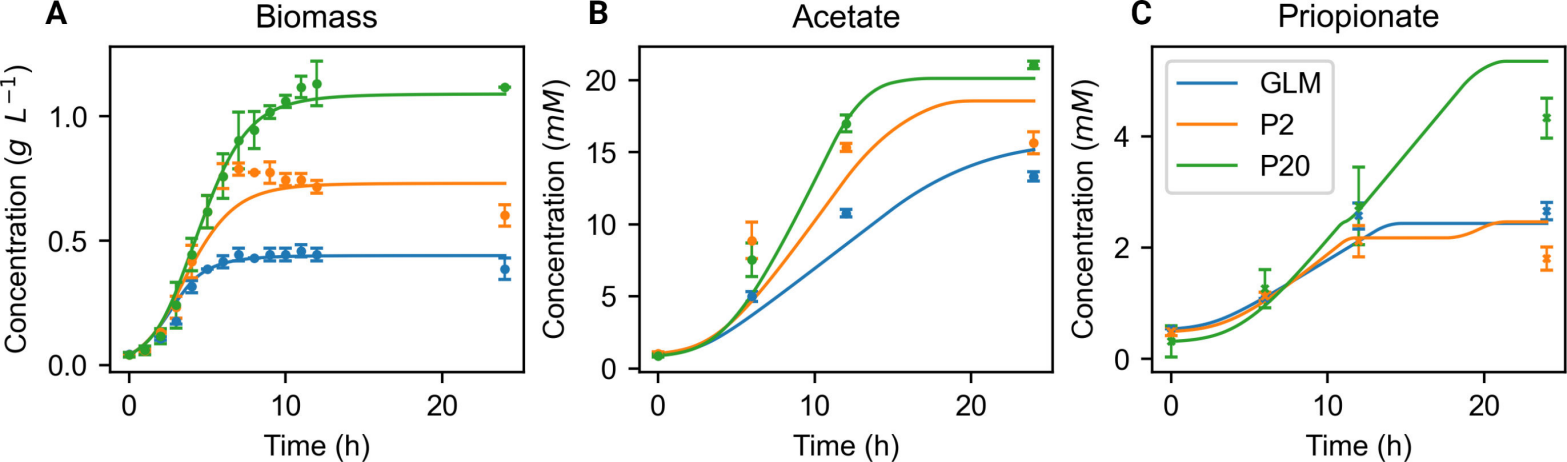
*B. caccae* dynamic flux balance analysis simulation in GLM, P2, and P20 *in silico* media. (**A**) *B. caccae* growth curve over 24 h in g·L^−1^. (**B**) Acetate concentration fate over 24 h (mM). (**C**) Propionate concentration fate over 24 h (mM). Values represented are mean ± SD from three independent experiments per medium (scatter plot) and model outputs (lines). Biomass concentration was converted from OD to g·L^−1^ to be compared to simulation results. Simulations were performed using the dFBA function of the CobraToolBox package.

We next compared the simulated and experimental biomass production curves (Fig. 6A). The model is able to properly predict *B. caccae* growth over 24 h in the three media as the predicted biomass is within biological variation for most time points (relative error of 4%, 8.7%, and 14% for the P20, GLM, and P2 media). Biomass prediction accuracy is slightly decreased at 24 h compared to 12 h. For the main produced metabolites (i.e., acetate and propionate), the concentrations are also better predicted at 12 h than at 24 h (Fig. 6B and C). Acetate prediction is also more accurate than propionate for the P20 medium, while propionate is better predicted in GLM and P2. Overall, glucose and lactose consumption are well reflected in GLM and P20 (*R*^2^ > 0.92; 0.82) as well as the production of acetate and propionate (*R*^2^ > 0.93; 0.80). In P2, produced metabolites are also well predicted (acetate: *R*^2^ > 0.79 and propionate: *R*^2^ > 0.70) (Fig. S2).

### Effect of the proteolytic modules and amino acids uptake

To assess the effect of the two proteolytic modules, we used dFBA simulation to compare the growth of *B. caccae* in four conditions with two (GLM and whey protein proteolytic modules), only one, or no proteolytic module in the P20 media. Note that the simulation with the two proteolytic modules corresponds to the P20 simulation, while the simulation with only GLM protein module corresponds to the GLM simulation. In the four conditions, the growth curves were similar, but the presence of the whey protein module was necessary to achieve the correct prediction of acetate and propionate production (Fig. S3), indicating that the whey protein amino acid content is key to activate these metabolic pathways.

To dig into the impact of amino acids on the growth, an *in silico* experiment was also conducted by limiting the uptake of individual or pairs of amino acids in the P20 model. Glutamate and asparagine were found to have the greatest impact on growth when access to these compounds was reduced, resulting in limited growth of *B. caccae* (Fig. S4).

## DISCUSSION

High-protein diets have become more frequent over the last 50 years in industrialized countries with recourse to the Western diet and the use of protein-enriched diets for specific indications or populations such as athletes or elderlies. Few studies showed a significant impact of high amounts of dietary proteins on the gut microbiota of adults (5, 33), but most of the reports on the effect of high amounts of whey proteins concerned infants showing that these proteins favored the development of *Bifidobacterium* and *Lactobacillus* genera (34–36). An *in vitro* study using stools from healthy 1–3 years old infants reported a significant increase in *Bacteroides*, *Proteobacteria*, and *Streptococcus* relative abundances and a rise in SCFA production (37). Using stools from normal-weight and obese individuals, Sanchez-Moya et al. (38) observed *in vitro* stimulation of *Bifidobacterium* and *Lactobacillus* growth and an enhanced SCFA production which could suggest an improvement of intestinal health. In adult mice, a protein supplementation resulted in an increased abundance of *Akkermansia muciniphila* and *Bacteroides uniformis,* two bacteria from the intestinal microbiota (39). Our experiments showed that the growth *B. caccae*, another intestinal microbiota member, increased with whey protein supplementation (i.e., in the P2 and P20 media compared to GLM). This in turn increased the metabolites consumption and production but had a limited impact on the per-capita metabolic fluxes.

The genome analysis revealed that *B. caccae* has an arsenal of 156 proteases including 31 potentially involved in nutrient purposes. Among these 31 proteases, 21 belonged to the core genome and are likely to be conserved in this species. In terms of protease content, *Bacteroides thetaioatomicron* genome was the closest to *B. caccae* with 98% of shared proteases while on average, other *Bacteroides* species genomes shared 81% of their protease content with *B. caccae*. We noted that the proteases involved in nutrition appeared to be the most variable within this genus, possibly reflecting different specificities and limiting competition for substrates. The proteases of the intestinal microbiota are still poorly characterized and the substrates and functional role of a protease are hardly predictable *in silico*. In *B. caccae*, 53% (16) of the proteases assigned to the protein turnover and 42% (13) of the proteases involved in bacterial feeding exhibited a signal peptide confirming that this bacterium actively degrades extracellular and periplasmic proteins prior to the internalization and subsequent catabolism of peptides or amino acids. This is consistent with the need to include a Contois kinetics in the model. This kinetic is indeed known to model the need for bacteria to be close or even attached to the substrate as well as the intracellular degradation (40).

We modeled the use of dietary proteins through proteolytic modules mimicking the degradation of proteins into free amino acids and the ATP cost associated with the biosynthesis of proteases. For the purpose of this study, we used two modules, the first one reflecting the proteolysis of proteins present in the three media (bactopeptone and meat extract) and the second one accounting for the proteolysis of whey protein. These modules were added to a metabolic model of *B. caccae* together with the amino acid transporters identified through genome mining. The model was inserted in a dFBA framework supplemented by dynamical regulations of the uptake of certain metabolites identified after an analysis of the flux dynamics. Finally, the model was calibrated and validated on independent data sets. Despite the simplified representation of the proteolytic activity, the resulting metabolic model accurately predicted the growth and the metabolites fate in the P2 validation set, unseen during fitting. Knocking out one or two proteolytic modules led to a less accurate prediction of acetate and propionate production while not affecting the growth prediction.

This study shows that ATP consumption associated with protease production is an important feature of the proteolytic model as it allowed a better fit with the experimental protein consumption and acetate, propionate and biomass productions. Without ATP consumption, the acetate and propionate productions exceeded the quantities measured experimentally by 30% (*R*^2^, 0.84–0.89) and 20% (*R*^2^, 0.8 to −0.24) in P20 and 50% for propionate in P2. Protein consumption was also too high without this ATP cost and a slight increase in predicted biomass production was observed during the exponential phase, suggesting the logistic regulation applied to the biomass reaction marginally affected the growth at low biomass levels. Overall, protease production (and therefore proteolysis) being inherently associated with ATP consumption*,* we established here that including this energy cost was necessary in the modeling of proteolysis to obtain an accurate metabolic prediction.

Regarding amino acids, glutamate and asparagine have been identified as having the largest impact on the growth model of *B. caccae* in P20. Glutamate is involved in the tricarboxylic acid (TCA) cycle, a central metabolic pathway for energy production (41). It can be converted to α-ketoglutarate, an intermediate in the TCA cycle. Limiting glutamate availability may affect the TCA cycle and the overall energy metabolism, thereby reducing the growth rate. Although asparagine is not directly involved in the TCA cycle, it can be converted to aspartate through the action of asparaginase. Aspartate can then be converted to oxaloacetate, another TCA cycle intermediate, by the aspartate aminotransferase enzyme. This reaction connects asparagine metabolism to the TCA cycle through the production of oxaloacetate, an essential TCA cycle component. In addition, asparagine and glutamate are precursors for the biosynthesis of other amino acids and secondary metabolites (18): asparagine can be converted to aspartate while glutamate is involved in the synthesis of glutamine, proline, and arginine. It is also possible that the glutamate catabolism leading into a variety of other amino acids and metabolites may make it particularly advantageous for the bacteria. Interestingly, the majority of amino acids can be either biosynthesized or catabolized by *B. caccae* (Fig. 4). Thus, it is rational that most amino acids can be readily substituted for one another. These findings, based on our model, provide valuable insights into the importance of specific amino acids for the growth and metabolism of *B. caccae*.

Our understanding of the interactions between diets and the microbiota has greatly advanced in recent years. However, a knowledge gap still exists in characterizing the proteolytic mechanisms of a number of gut bacteria, including the prevalent *Bacteroides* species. Specifically, the exact roles of proteases, including their substrate preferences and functional contributions, remain largely elusive. Interestingly, a contrasting degree of knowledge can be observed when we look at dietary fiber degradation in the human gut microbiota, where the function of glycoside hydrolases (GH) has been extensively studied and characterized (42). The substrate specificity and roles of these GH enzymes in fiber degradation are well-defined, providing a solid baseline to generate predictive models for understanding bacterial interactions with dietary fibers (43, 44). The lack of knowledge about proteases is a major obstacle to the development of accurate predictive models of bacterial proteolytic activity.

Our study took a step toward addressing this need, offering new insights into *B. caccae*’s proteolytic activity by demonstrating the growth and metabolite production prediction ability of our proteolytic model. The pan-genome analysis highlights the conservation of proteases across different strains of *B. caccae* and suggests the potential for our model to be generalized across this species. In addition, the conservation of proteases in other *Bacteroides* such as *Bacteroides thetaiotaomicron* opens the possibility of extending our model to other *Bacteroides* species. In the future, this proteolytic model could be applied to predict proteolytic activity in a range of bacterial species ultimately contributing to the development of more complete community models accurately representing the proteolytic functions within complex microbial ecosystems.

### Conclusion

Our study showed that the presence of whey protein improved the growth and production of fatty acids of *B. caccae*, a gut commensal bacterium. Coupling experiments, genome mining, and modeling provided valuable insights into the proteolysis pathway and nutritional requirements of *B. caccae*, which can deepen our understanding of the role of proteolysis in bacterial physiology and ecology. The mathematical model developed to predict *B. caccae* growth on whey proteins demonstrated good agreement with experimental data and can be easily generalized to other gut bacteria. Overall, this study highlights the importance of considering dietary proteins as a potential driver of the microbial populations in the gut.

## MATERIALS AND METHODS

### Strain and culture conditions

*B. caccae* ATCC 43185 also known as DSM 19024 was grown in Glucose Limited Medium [GLM; (25)] containing: D-glucose 5 g L^−1^, bactopeptone 5 g L^−1^, meat extract 5 g L^−1^, sodium pyruvate 1 g L^−1^, sodium succinate 1 g L^−1^, CaCl_2_ 0.01 g L^−1^, KH_2_PO_4_ 0.04 g L^−1^, K_2_HPO_4_ 0.04 g L^−1^, NaCl 0.08 g L^−1^, tween 80 0.1% (vol/vol), ZnSO_4_ 7H_2_O, 0.02 g L^−1^, NaHCO_3_ 0.4 g L^−1^, L-cysteine HCl 0.5 g L^−1^, phylloquinone 1·10^−6^ g L^−1^, thiamine 5·10^−5^ g L^−1^, riboflavin 5·10^−5^ g L^−1^, biotine 2·10^−5^ g L^−1^, and hemin 0.005 g L^−1^. The P2 and P20 media were GLM supplemented with 2 and 20 g L^−1^ of Prolacta 95, respectively (Lactalis ingredients, Bourgbarré, Fr). Although glucose remained the main sugar in all media, a small amount of lactose was detected in GLM and slightly increased with Prolacta 95 supplementation as follows: GLM 0.16 ± 0.006 g L^−1^, P2 0.19 ± 0 g L^−1^, and P20 0.36 ± 0.006 g L^−1^. To avoid the opaque white medium obtained after autoclaving P20, the three media were filtered (0.22 µM). This step modified the final protein concentration as follows: GLM: 7.28 ± 0.2 g L^−1^, P2: 8.51 ± 0.38 g L^−1^, and P20: 21.87 ± 0.9 g L^−1^. After inoculation, the cultures were incubated at 37°C in a cabinet (Jacomex, Dagneux, Fr) under anaerobiosis (BIO300: CO_2_ 5%, H_2_ 5% N_2_ 90%, Air Liquide, Les-Loges-en-Josas, Fr). Precultures in either GLM, P2 or P20, were inoculated from a fresh colony, incubated overnight and used to inoculate the cultures used for the growth kinetics. The bacterial growth was monitored over time through optical density measured at 600 nanometers (nm). Culture samples were harvested at different time points, centrifuged at 8,000 rpm for 11 min at room temperature, the supernatants were then collected and stored at −20°C prior to metabolites quantification.

### Quantification of substrates and fermentation products

Glucose, lactose, SCFAs, BCFAs, and succinate concentrations were measured in culture supernatants (500 µL) using reverse-phase high-performance chromatography (HPLC) instrument (Alliance HPLC Waters, e2695W, Guyancourt, France). Acquisition of spectra and data analysis were performed using Empower 3 (Waters, Guyancourt, France). The amounts of whey proteins were measured using the Pierce BCA protein assay kit (Thermo Scientific, Rockford, IL, USA). Amino acids were quantified from filtered samples (0.22 µm) and diluted when required in Formic acid 1% for analysis by UHPLC-MS (UHPLC Ultimate 3000 and HR-MS-Q exactive, Thermo Scientific). UHPLC conditions: metabolites were separated on Hypersil Gold phenyl (Length = 15 mm, Internal diameter = 2.1 mm, and Particles size = 3 µm, Thermo Scientific). The pressure at the beginning of the gradient was 130 bar and the column temperature was 25°C. The flow was 0.25 mL min^−1^ and the solvent was A, acetonitrile quality HPLC MS and B, water ultra-pure quality HPLC MS with nonafluoropentanoic acid (3 mM). The elution gradient was as follows: 4 min at 98% B + 2% A, then 98% to 2% B in A for 6 min, level at 2% B and 98% A for 3 min. The injection volume was 5 µL and the injector temperature was 7°C. The duration of one analysis was 14 min. Mass spectrometric detection was performed with a Hybrid Quadrupole-Orbitrap with a heated electrospray source HESI operated in the positive ionization mode. Full scans were acquired with a scan range of 3.7 scan/s and a mass range from 60 to 850 u.m.a. (unified atomic mass unit) or Dalton (Da) with a resolution of 70,000. Data were identified and quantified using Trace Finder software according to the calibration solution.

### Statistical analysis

A non-parametric Kruskal–Wallis test by ranks was used to assess the statistical significance of differences between measures. *P* value ≤ 0.05 was set up as the threshold of significant change.

#### Mixed models for metabolite flux prediction

To approximate metabolite flux in the data, for a metabolite *m*, a bacterial load *b* and two consecutive times *t*_0_ and *t*_1_, we computed the production rate *r* normalized by the bacterial load between *t*_0_ and *t*_1_ (defined as the mean of the bacterial loads at time *t*_0_ and *t*_1_), with equation (1).


(1) $$r_{t_{0}t_{1}}^{m}=\frac{m\left(t_{1}\right)-m\left(t_{0}\right)}{\left(t_{1}-t_{0}\right)\frac{b\left(t_{0}\right)+b\left(t_{1}\right)}{2}}$$

Bacterial OD measures were converted into biomass using a conversion coefficient of 0.43, a value derived and validated through assessments involving the measurement of dry weight in triplicate.

Next, for each metabolite, we modeled the production rate in a mixed model framework as follows:


(2) $$r_{t,s,i}^{m}∼R_{t}^{m}+D_{s}^{m}+\varepsilon_{t,s,r}^{m}$$

where ${r^{m}}_{t,s,r}$ is the observed production rate for metabolite *m*, time interval $t\in \{`t_{0}t_{6}`,`t_{6}t_{12}`,`t_{12}t_{24}`\}$, substrates s∈{GLM,P2,P20} and replicate i∈{1,2,3}. The terms ${R^{m}}_{t}$ and ${D^{m}}_{s}$ are the fixed effects for the time intervals *t* and substrate *s*. The random term ${\varepsilon^{m}}_{t,s,r}$ represents normal zero-mean independent errors. To avoid identifiability issues, the fixed effect ${D^{m}}_{s}$ was set to 0 for s=GLM. Next, we selected the metabolites with a significant estimate (fdr <0.05, *t* test, Benjamini Hochberg correction for multiple tests) of a fixed effect for substrate P2 and P20, indicating significant difference between production rates in different substrates. The mixed model was inferred using the Python package statsmodels with the mixedlm function.

### Genome analysis

*B. caccae* ATCC 43185 reference genome was downloaded from the NCBI database (28). The MEROPS database (27) and a tblastn following NCBI recommendations (45) using the default parameters were used on the *B. caccae* reference genome to identify putative proteases. Proteases with a percentage of identity >30%, an *e*-value <0.05, a bitscore >50, and a query coverage >75% were conserved in the data set. Enzymes corresponding to protease inhibitors were eliminated from the data set. SignalP V6 (46) and Phobius (47) were used to detect the presence of signal peptides; the obtained results with the methods were consistent. DeepTM HMM (48) as used to predict transmembrane proteins. All three prediction tools were used using default parameters. Transporters were identified using the metabolic pathway prediction tool box GAPSEQ (49) with default parameters. Further manual curation was performed using KEGG.

#### Pan-genomes

Forty-seven *B. caccae* genomes with less than 50 scaffolds allowing a coverage above 50-fold were retrieved from the NCBI database (28) (Table S3). Genome assemblies were improved in order to reduce the number of contigs using Ragtag (50) and the reference genome ASM222261v2. All the genomes were annotated using Prokka (51) with the default setting. *B. caccae* pan-genome was created using the Roary pipeline (52) with the default setting. The level of conservation between the various *B. caccae* genomes of the ATCC 43185 protease genes was determined using tblastn (45) on the pan-genome sequences.

### Modeling approach

#### Manual curation of the Seed model

The metabolic model for *B. caccae* [version AGORA 1.03 (23)] was sourced from the Virtual Human database (https://www.vmh.life/). The AGORA2 model was considered for performing the *in silico* experiments. However, certain aspects of its design proved incompatible with our experimental setup. A critical factor in this decision was the inclusion of a specific metabolite, KDO(2)-lipid IV(A), in the biomass reaction of AGORA2 (53). This metabolite is a component of the endotoxin synthesis pathway and depends on the presence of the metabolite acgam[c] (N-acetyl-D-glucosamine), which is derived from mucin degradation. This makes mucin or acgam[c] availability mandatory for AGORA2 model’s growth. Conversely, the AGORA1 model, despite containing acgam[c] and pathways for mucin degradation, does not require these components for growth. As mucin or acgam[c] was not present in our experimental media, we chose the AGORA1 model for our study.

In its initial form, the model incorporated 730 genes, 952 metabolites, and 1225 reactions. For subsequent analyses, the model was exported in MAT format, enabling its compatibility with the COBRA toolbox (31). To improve model completeness, a rigorous manual curation process was performed. This curation primarily centered on amino acid metabolism. First, the model was assessed to ascertain the presence of uptake reactions for each amino acid and other nutrients available in our specified experimental media. Absent import reactions were supplemented accordingly. Second, both biosynthesis and degradation pathways of amino acids were rigorously checked using gapseq (49) for the identification of potential pathway gaps. Verification was subsequently performed using KEGG maps (54). Finally, additional transporters identified with gapseq were verified with literature-based evidence to make informed decisions on the types of transporters utilized for specific amino acids. All the missing transporters identified through this process were integrated into the model.

Calibration and validation of carbon source utilization predicted by the model were performed with experimental data. We ensured the model could generate the secreted products that had been detected during the biological experiments. The media used as input to perform flux balance analysis growth simulations were defined based on the media characteristics (Table S5). Manual curation comprised the addition of several exchange reactions and transporters (Table S4). Upper and lower bounds on exchange reactions were assigned based on experimental data retrieved from the lab experiments.

#### Proteolytic module design

Two proteolytic modules were developed for this study, according to the following generic equation:


(3) $$Protein+\chi ATP\to \sum_{i=1}^{n}R_{i}.AA_{i}$$

In this equation (3), *R*_*i*_ is the amount of the ith amino acids (AA) in the list of the different amino acids present in the protein and χ corresponds to the equivalent-ATP energy cost of protease biosynthesis needed for proteolysis. The *R_i_* were computed from the mean amino acids content of the proteins present in the media. The first module was designed to degrade whey protein, and the second module was designed to degrade the other proteins present in the culture medium.

The whey protein module was developed by considering the two main proteins present in whey protein, beta-lactoglobulin (β-LG) and alpha-lactalbumin (α-LA). Our hypothesis was that one unit of whey protein would produce a number of amino acids corresponding to the mean of the stoichiometric composition of β-LG and α-LA. To account for other proteins in the medium that were not part of the whey protein, a second proteolytic module was designed. The number of amino acids produced from the degradation of these proteins was determined based on the amino acid composition of the protein-containing elements in the medium, such as bactopeptone and meat extract.

To estimate the energy cost associated with the proteolytic reactions, we used the method described by Smith and Chapman (55) to calculate the ATP cost of synthesizing an enzyme based on its amino acid composition. We determined the average ATP cost for producing the 31 proteases involved in nutrition present in *B*. *caccae* by taking into account the amino acid composition of these enzymes and found 23 moles of ATP for one mole of protease.

#### MEMOTE analysis

To ensure the robustness and accuracy of our metabolic model, we employed MEMOTE, a comprehensive tool for metabolic model validation (32). This systematic analysis allowed us to identify areas for improvement and validate our model integrity.

We integrated metabolite and reaction annotations from all relevant databases used in the AGORA2 model into our model. This process was crucial for enhancing the annotation coverage and depth of our model. Additionally, we undertook manual corrections of certain annotations, guided by insights and recommendations derived from MEMOTE’s analysis.

#### Kinetic regulations

Kinetic regulations were added on glucose, protein, and lactose, based on the rough evaluation of metabolic fluxes made during the experiments (Fig. 2). First, we assumed a Monod kinetic for the glucose consumption which led to the following regulation:


(4) $${lowerbound}_{i,t+1}=\max\left(\frac{{\left[metabolite\right]}_{i,t}}{\left[biomass\right]*timeStep},l_{int}\right)*\frac{{\left[metabolite\right]}_{i,t}}{K+{\left[metabolite\right]}_{i,t}}$$

In equation (4), the lower bound of the metabolite *i* (here glucose) at a time point *t* + 1 is recalculated at each time point, [metabolite]*_i,t_* being the concentration of the observed metabolite *i* at time point *t* and *Κ* is an estimated constant specific to the metabolite. The first factor in this formula is the classical lower bound for metabolic uptake in dFBA, the second one is the Monod kinetic.

A Contois kinetic was chosen for proteins and lactose consumption. This regulation is classical to model hydrolysis (56).


(5) $${lowerbound}_{i,t+1}=\max\left(\frac{{\left[metabolite\right]}_{i,t}}{\left[biomass\right]*timeStep},l_{int}\right)*\frac{{\left[metabolite\right]}_{i,t}}{K{\left[biomass\right]}_{t}\;+\;{\left[metabolite\right]}_{i,t}}$$

This equation (5) is similar to the Monod’s equation (4), in which the estimated constant *Κ* is further modulated by the biomass concentration at the time point *t*.

#### Carrying capacities

A carrying capacity has been added to the growth model that represents the species population size limitation depending on environmental factors (here the initial concentration in whey protein) in the dFBA biomass (equation (7)) and used GLM as a growth baseline. Namely, we defined the carrying capacity as follows:


(6) $$carryingcapacity=T_{GLM}+\frac{{\left[WP\right]}_{initial}^{HillCoef}}{K+{\left[WP\right]}_{initial}^{HillCoef}}$$

(7) $$\frac{d{\left[Biomass\right]}_{t}}{dt}=\;\lambda \left(1-\frac{{\left[Biomass\right]}_{t}}{carrying\;capacity}\right)\mu_{FBA}{\left[Biomass\right]}_{t}$$

The carrying capacity is described by the sum of T_GLM_, the maximum GLM biomass concentration during the plateau phase, and a Hill equation on [WP], the initial concentration in whey protein, with Hill exponent Hillcoef and a parameter *K*. Equation (7) models the biomass evolution with the global growth rate $\lambda \left(1-\frac{{\left[Biomass\right]}_{t}}{carrying\;capacity}\right)\mu_{FBA}$ that can be decomposed as the FBA growth rate $\mu_{FBA}$ computed according to the current nutritional environment, modulated by the logistic regulation $\lambda \left(1-\frac{{\left[Biomass\right]}_{t}}{carrying\;capacity}\right)$ with parameters λ and carryingcapacity. This modulation allows for the dFBA to predict both the exponential and stationary phases.

#### *In silico* growth simulations

The COBRA Toolbox (31) (https://github.com/opencobra/cobratoolbox.git) was run on MATLAB 2018b (The MathWorks Inc., Natick, MA, USA) and used for FBA and dynamicFBA simulation. A homemade MATLAB script was used for the dynamicFBA simulation to allow for the added regulations (i.e., Monod and Contois regulations) which are available on GitHub (https://github.com/apaulay/ProteolyticModule). This script is an adaptation of the dynamicFBA.m function from the CobraToolBox, augmented with a logistic regulation term linked to the biomass growth component.

Our approach integrated a semi-explicit Euler scheme for computing the evolution of biomass. Namely, the logistic regulation was made explicit, and an exact integration of the resultant linear ODE was applied to deduce the biomass at the subsequent time step. For the evolution of metabolite concentrations, we used a semi-implicit scheme. Here, metabolites with negative fluxes (indicative of consumption) were resolved using an implicit Euler scheme, while those with positive fluxes (indicating production) were calculated via an explicit Euler scheme. This semi-implicit approach ensures numeric positivity throughout the integration process, enabling the maintenance of a constant time step without instigating positivity issues. The biomass production rate was calculated using the exponential phase growth curve, and metabolite production rates were calculated on the first 12 h of growth.

### Model optimization

The model was calibrated from data following two independent steps. First the parameters of equations 4-6 were fitted with non-linear regression on the flux time-series for glucose, lactose and proteins (Fig. 2 and M&M section on flux prediction). Then, upper and lower bounds of the FBA model were fitted using least square inference with the dFBA simulations. Namely, setting *θ* the set of five parameters to be fitted (glycine upper bound, asparagine lower bound, a parameter common to the remaining amino acid upper and lower bounds, the logistic parameter *λ* in GLM and *λ* in P20), the parameters were inferred using the Matlab function *fmincon,* using the least-square observation function (equation 8).


(8) $$f\left(\theta \right)=\sum_{j\in optim}\frac{{\left\|m{\left(\theta \right)}_{j}-m_{exp,j}\right\|}^{2}}{{\left\|\sigma \right\|}_{exp,j}^{2}}$$

where $m(\theta {)}_{j}$ is the dFBA model output for the *j*-th time series for the parameter values θ, $m_{exp,j}$ is the corresponding observed time-serie in the data, and $\sigma_{exp,j}$ its standard deviation for three replicates. The set *optim* is the set of observables on which the optimisation is made. We took:


(9) optim={biomass,acetate,propionate,glucose,lactose}

a maximal number of 100,000 evaluations was set in *fmincom* parameters.

## SUPPLEMENTAL MATERIAL

Supplemental Figures msystems.00153-24-s0001.pdf Fig. S1 to S5.

Supplemental Tables msystems.00153-24-s0002.docx Tables S1 and S3 to S5.

Table S2 msystems.00153-24-s0003.docx Protease in *B. caccae* ATCC 43185 genome.

ASM does not own the copyrights to Supplemental Material that may be linked to, or accessed through, an article. The authors have granted ASM a non-exclusive, world-wide license to publish the Supplemental Material files. Please contact the corresponding author directly for reuse.
