# Supplementary material for: Design of a proteolytic module for improved metabolic modeling of Bacteroides caccae
Source: mSystems. 2024 Mar 22;9(4):e00153-24. doi: 10.1128/msystems.00153-24 (PMC11019848; doi:10.1128/msystems.00153-24)
Supplement: Supplemental Figures — Fig. S1 to S5. [file msystems.00153-24-s0001.pdf]

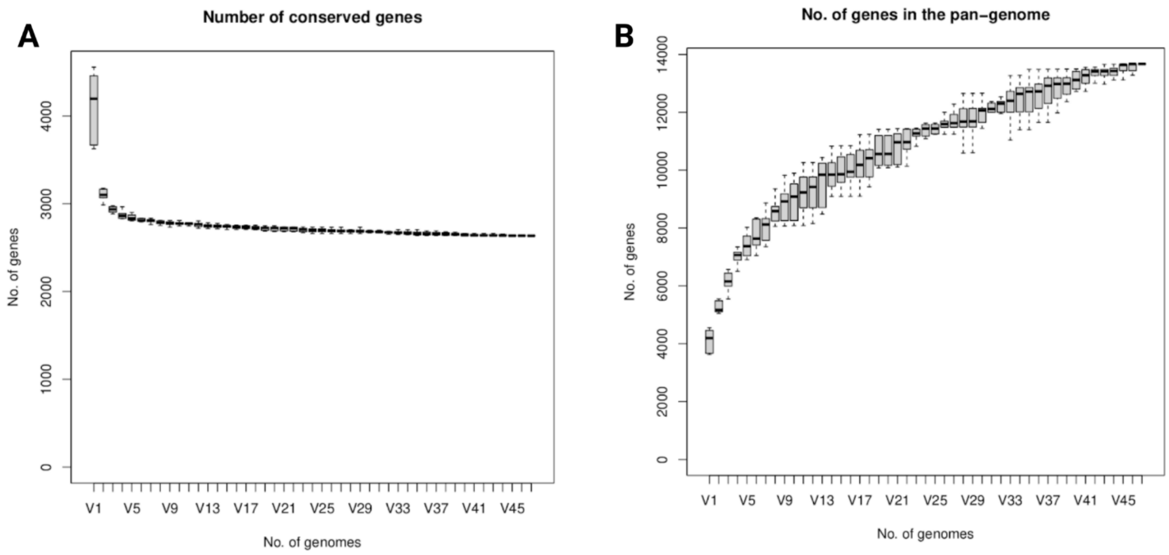

Figure S1. *B. caccae* pangenome analysis. A, Number of conserved genes among the 47 genomes. B, Number of genes in the pangenome.

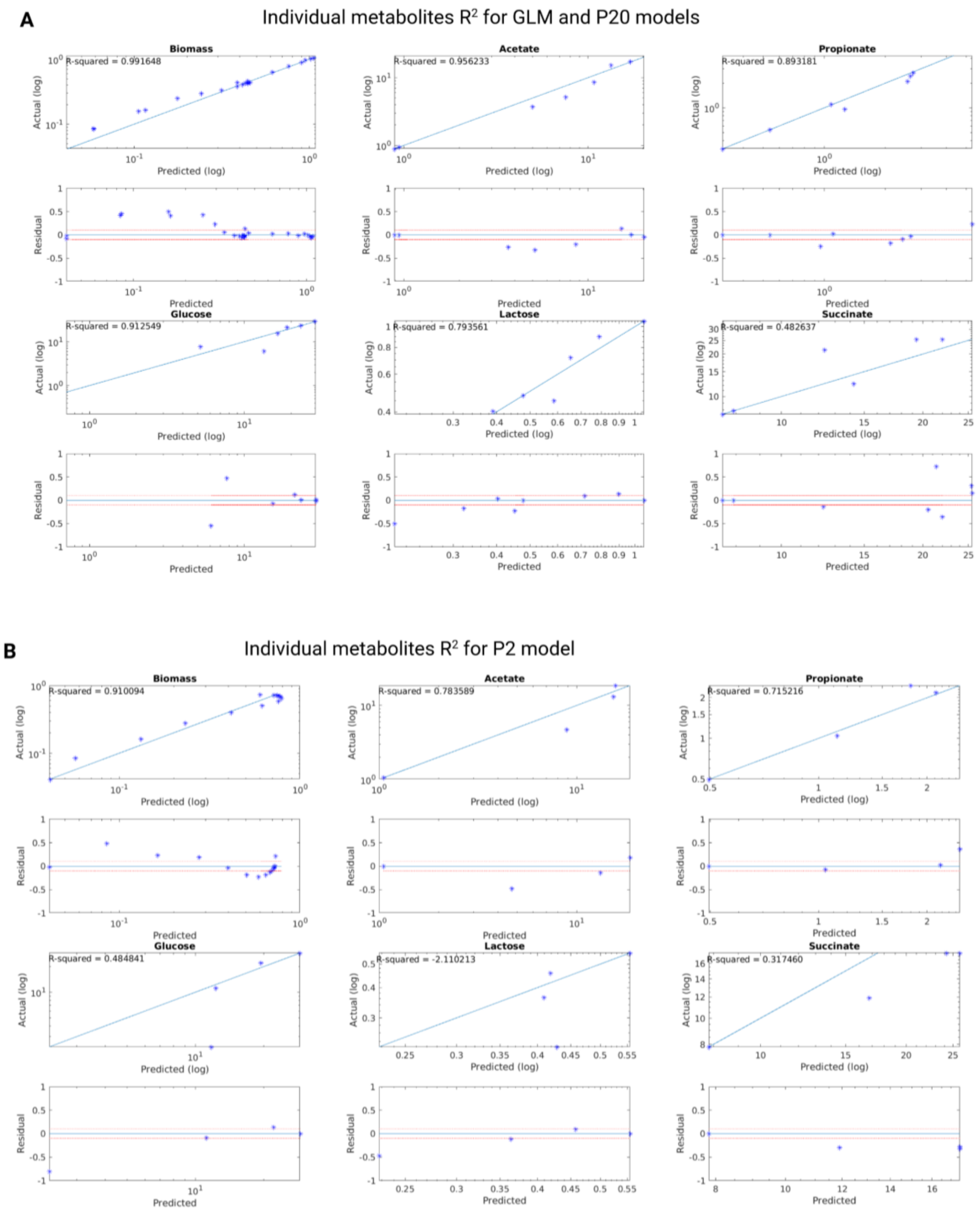

Figure S2. Goodness of fit of individual metabolites in GLM, P2 and P20 media. (a) The individual R^2 of biomass and selected metabolites for the GLM and P20 media. (b) The individual R^2 of biomass and selected metabolites for the P2 medium. For Biomass and each metabolite, the top plot displays the experimental (actual) values versus the corresponding predicted values computed with the model in log. log. scale, together with the bissector (blue line). The bottom plot displays for each model prediction the corresponding relative residual error. The red dotted lines represent a ± 5% error; a vast majority of points are kept in the range ± 10% error.

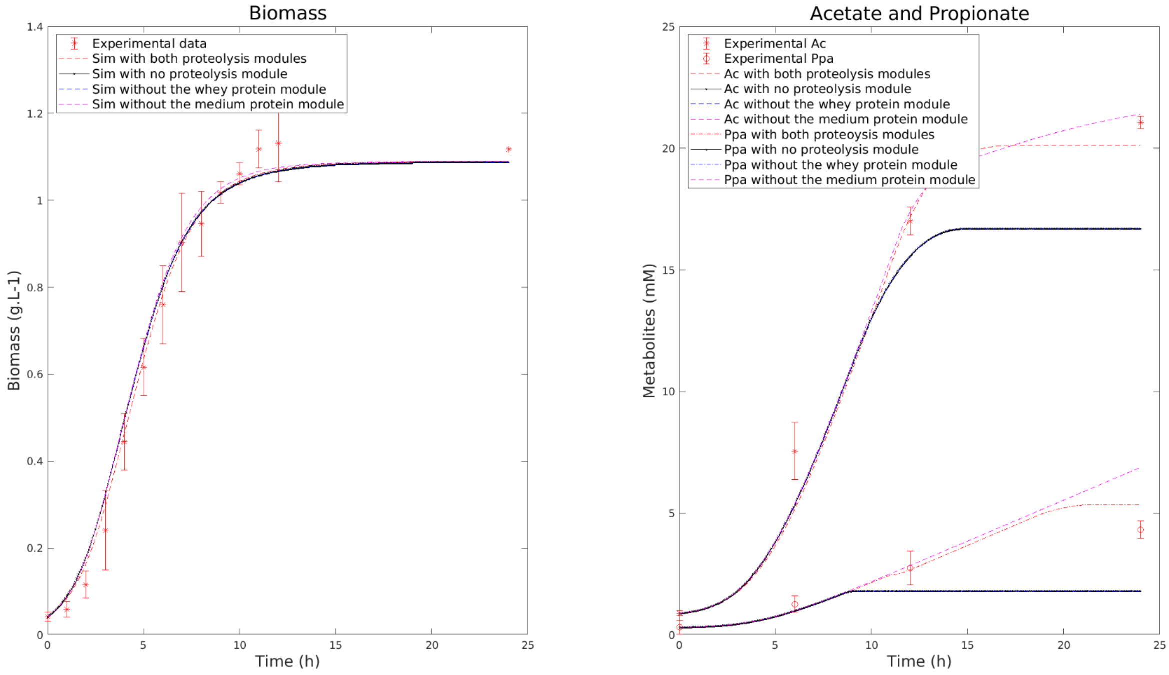

Figure S3. dFBA simulations of biomass, acetate and proprionate with or after knock out (KO) of the proteolysis modules in P20. Dotted red lines, simulations with the two proteolysis modules for whey and medium proteins. Dotted pink lines, simulations after KO of the whey proteins proteolysis module. Dotted blue lines, simulations after KO of the medium proteins. Black lines, simulations after knock out of both proteolytic modules. Experimental data are shown as red stars for biomass and acetate and red dots for propionate with error bars. In both graphs, the blue and black curves overlap each other. Ppa, propionate. Ac, acetate.

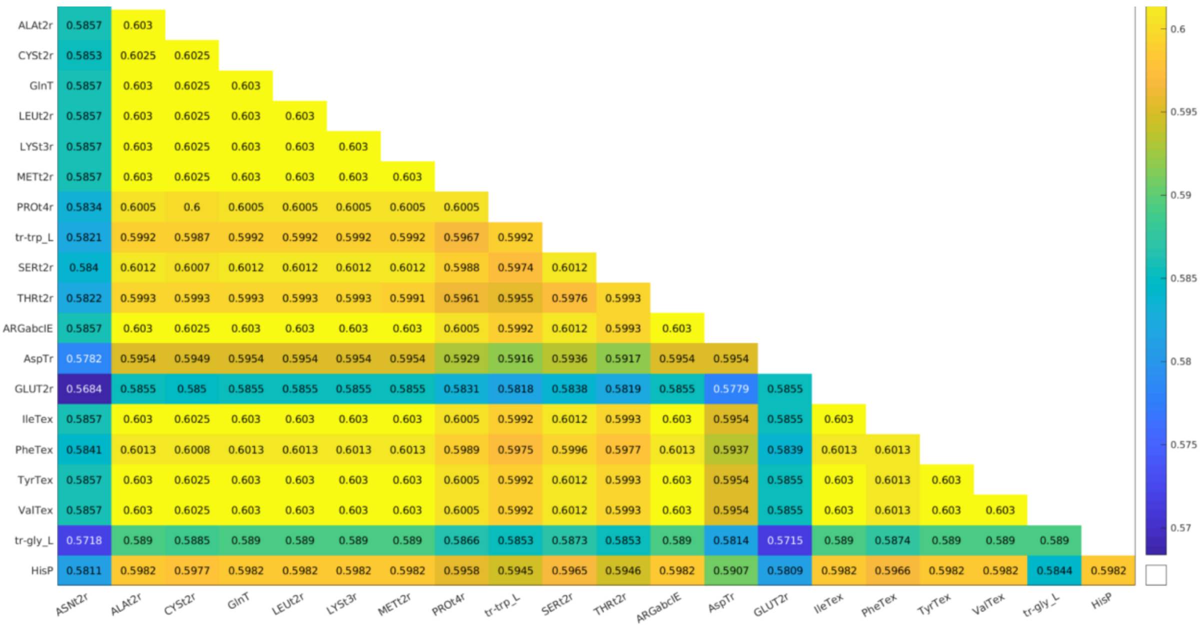

Figure S4. Heat map of the simulated growth rate in P20 after pairwise limitation of amino acids availability. We reduced the availability of 2 amino acids at the same time, in order to assess potential interactions between amino acids degradation pathways and then simulated the growth of *B. caccae* in P20 using our FBA model. The reference value with all amino acids was 0.60 h^{-1}.

Consistency

Compared Models	Bacteroides_caccae_ATCC_43185_AGORA2.xml	Bacteroides_caccae_ATCC_43185_AGORA1.xml	Bacteroides_caccae_ATCC_43185_P20.xml
Stoichiometric Consistency	100.0%	100.0%	100.0%
Mass Balance	99.8%	99.8%	99.8%
Charge Balance	99.7%	99.8%	99.7%
Metabolite Connectivity	100.0%	100.0%	100.0%
Unbounded Flux In Default Medium	0.0%	0.0%	0.0%
Sub Total	86%	86%	86%

Annotation - Metabolites

Compared Models	Bacteroides_caccae_ATCC_43185_AGORA2.xml	Bacteroides_caccae_ATCC_43185_AGORA1.xml	Bacteroides_caccae_ATCC_43185_P20.xml
Presence of Metabolite Annotation	100.0%	32.8%	100.0%
Metabolite Annotations Per Database			Info
pubchem.compound	56.1%	28.9%	45.0%
kegg.compound	49.1%	32.0%	50.8%
seed.compound	57.0%	0.0%	65.8%
inchikey	0.0%	0.0%	0.0%
inchi	63.9%	18.1%	53.2%
chebi	50.9%	0.0%	49.6%
hmdb	44.3%	30.1%	42.9%
reactome	0.0%	0.0%	0.0%
metanetx.chemical	50.3%	0.0%	55.8%
bigg.metabolite	100.0%	0.0%	71.5%
biocyc	99.9%	0.0%	71.4%

Metabolite Annotation Conformity Per Database				Info
pubchem.compound	100.0%	100.0%	100.0%	
kegg.compound	100.0%	100.0%	100.0%	
seed.compound	100.0%	0.0%	100.0%	
inchikey	0.0%	0.0%	0.0%	
inchi	100.0%	100.0%	100.0%	
chebi	100.0%	0.0%	100.0%	
hmdb	6.5%	100.0%	6.6%	
reactome	0.0%	0.0%	0.0%	
metanetx.chemical	100.0%	0.0%	100.0%	
bigg.metabolite	100.0%	0.0%	100.0%	
biocyc	99.9%	0.0%	99.8%	
Uniform Metabolite Identifier Namespace	0.0%	0.0%	0.0%	
Sub Total	56%	20%	55%	

Annotation - SBO Terms

Compared Models	Bacteroides_caccae_ATCC_43185_AGORA2.xml	Bacteroides_caccae_ATCC_43185_AGORA1.xml	Bacteroides_caccae_ATCC_43185_P20.xml
Metabolite General SBO Presence	100.0%	0.0%	100.0%
Metabolite SBO:0000247 Presence	100.0%	0.0%	100.0%
Reaction General SBO Presence	100.0%	0.0%	100.0%
Metabolic Reaction SBO:0000176 Presence	99.6%	0.0%	99.9%
Transport Reaction SBO:0000185 Presence	100.0%	0.0%	100.0%
Exchange Reaction SBO:0000627 Presence	100.0%	0.0%	100.0%
Demand Reaction SBO:0000628 Presence	76.9%	0.0%	100.0%
Sink Reactions SBO:0000632 Presence	100.0%	0.0%	100.0%
Gene General SBO Presence	100.0%	100.0%	100.0%
Gene SBO:0000243 Presence	100.0%	100.0%	100.0%
Biomass Reactions SBO:0000629 Presence	100.0%	0.0%	100.0%
Sub Total	98%	18%	100%
Total Score	83%	45%	84%

Figure S5. MEMOTE analysis comparing AGORA2 (here noted Bacteroides_caccae_ATCC_43185_AGORA2.xml), AGORA1 (here noted Bacteroides_caccae_ATCC_43185_AGORA1.xml), and our version version (here noted Bacteroides_caccae_ATCC_43185_P20.xml).
