# Supplementary material for: Design of a proteolytic module for improved metabolic modeling of Bacteroides caccae
Source: mSystems. 2024 Mar 22;9(4):e00153-24. doi: 10.1128/msystems.00153-24 (PMC11019848; doi:10.1128/msystems.00153-24)
Supplement: Supplemental Tables — Tables S1 and S3 to S5. [file msystems.00153-24-s0002.docx]

| **Table S1.** Measures of amino acids during the growth of *B. caccae* in GLM, P2, and P20. | | | | | | | | |  |
| --- | --- | --- | --- | --- | --- | --- | --- | --- | --- |
|  |  |  |  |  |  |  |  |  |  |
|  |  | **G0** | | **G2** | | **G20** | |  |  |
|  | ***Time*** | ***Mean (g L^-1^)*** | ***SD*** | ***Mean (g L^-1^)*** | ***SD*** | ***Mean (g L^-1^)*** | ***SD*** | ***p-value*** |  |
| **Alanine** | 0 | 0.2800 | 0.0000 | 0.2600 | 0.0082 | 0.2600 | 0.0141 | 0.11 |  |
|  | 12 | 0.3200 | 0.0082 | 0.3167 | 0.0403 | 0.3667 | 0.0170 | 0.07 |  |
| **Arginine** | 0 | 0.4433 | 0.0094 | 0.4367 | 0.0047 | 0.4233 | 0.0125 | 0.21 |  |
|  | 12 | 0.4633 | 0.0125 | 0.4867 | 0.0125 | 0.4967 | 0.0125 | 0.12 |  |
| **Glycine** | 0 | 0.1100 | 0.0000 | 0.1100 | 0.0000 | 0.1100 | 0.0000 | *NaN* |  |
|  | 12 | 0.1833 | 0.0047 | 0.2633 | 0.0047 | 0.3100 | 0.0163 | 0.02 |  |
| **Isoleucine** | 0 | 0.1300 | 0.0000 | 0.1300 | 0.0000 | 0.1233 | 0.0047 | 0.10 |  |
|  | 12 | 0.1233 | 0.0047 | 0.1367 | 0.0047 | 0.1500 | 0.0082 | 0.04 |  |
| **Aspartic acid** | 0 | 0.1133 | 0.0047 | 0.1100 | 0.0000 | 0.1133 | 0.0047 | 0.56 |  |
|  | 12 | 0.1333 | 0.0047 | 0.1067 | 0.0047 | 0.1033 | 0.0047 | 0.05 |  |
| **Cystine** | 0 | 0.0700 | 0.0000 | 0.0800 | 0.0000 | 0.0933 | 0.0047 | 0.02 |  |
|  | 12 | 0.0200 | 0.0000 | 0.0100 | 0.0000 | 0.0033 | 0.0047 | 0.03 |  |
| **Glutamic acid** | 0 | 0.2933 | 0.0047 | 0.2933 | 0.0047 | 0.2967 | 0.0125 | 0.86 |  |
|  | 12 | 0.3233 | 0.0125 | 0.3733 | 0.0125 | 0.4533 | 0.0403 | 0.03 |  |
| **Histidine** | 0 | 0.0600 | 0.0000 | 0.0600 | 0.0000 | 0.0600 | 0.0000 | *NaN* |  |
|  | 12 | 0.0733 | 0.0047 | 0.0833 | 0.0047 | 0.0867 | 0.0047 | 0.10 |  |
| **Serine** | 0 | 0.1300 | 0.0000 | 0.1233 | 0.0047 | 0.1167 | 0.0047 | 0.06 |  |
|  | 12 | 0.1300 | 0.0082 | 0.1300 | 0.0000 | 0.1267 | 0.0047 | 0.73 |  |
| **Leucine** | 0 | 0.3067 | 0.0047 | 0.3033 | 0.0047 | 0.2933 | 0.0125 | 0.40 |  |
|  | 12 | 0.2933 | 0.0125 | 0.3033 | 0.0047 | 0.3233 | 0.0125 | 0.09 |  |
| **Threonine** | 0 | 0.1000 | 0.0000 | 0.1000 | 0.0000 | 0.0867 | 0.0047 | 0.02 |  |
|  | 12 | 0.1033 | 0.0047 | 0.1067 | 0.0047 | 0.1033 | 0.0047 | 0.67 |  |
| **Tyrosine** | 0 | 0.0733 | 0.0047 | 0.0700 | 0.0000 | 0.0700 | 0.0000 | 0.36 |  |
|  | 12 | 0.0767 | 0.0047 | 0.0800 | 0.0000 | 0.0733 | 0.0047 | 0.26 |  |
| **Valine** | 0 | 0.1533 | 0.0047 | 0.1533 | 0.0047 | 0.1467 | 0.0047 | 0.30 |  |
|  | 12 | 0.1767 | 0.0047 | 0.2000 | 0.0000 | 0.2167 | 0.0125 | 0.03 |  |
| **Lysine** | 0 | 0.2433 | 0.0047 | 0.2433 | 0.0047 | 0.2333 | 0.0047 | 0.13 |  |
|  | 12 | 0.2733 | 0.0047 | 0.2867 | 0.0047 | 0.3000 | 0.0163 | 0.08 |  |
| **Methionine** | 0 | 0.0700 | 0.0000 | 0.0700 | 0.0000 | 0.0667 | 0.0047 | 0.36 |  |
|  | 12 | 0.0733 | 0.0047 | 0.0733 | 0.0047 | 0.0733 | 0.0047 | 1 |  |
| **Phenylalanine** | 0 | 0.2000 | 0.0082 | 0.1933 | 0.0047 | 0.1900 | 0.0163 | 0.63 |  |
|  | 12 | 0.2000 | 0.0082 | 0.2100 | 0.0082 | 0.2133 | 0.0125 | 0.40 |  |
| **Proline** | 0 | 0.0600 | 0.0000 | 0.0600 | 0.0000 | 0.0600 | 0.0000 | *NaN* |  |
|  | 12 | 0.1167 | 0.0047 | 0.1900 | 0.0000 | 0.2267 | 0.0125 | 0.02 |  |
| The amino-acids were measured in cultures supernatants. Mean values ± SD from 3 independent experiments are shown. A one-way ANOVA on ranks was performed to compare the 3 media. *NaN*, no one-way ANOVA p-value prediction due to perfectly identifical dosage measures. Green, highlights p-value ≤ 0.05. | | | | | | | | |  |
|  |  |  |  |  |  |  |  |  |  |
|  |  |  |  |  |  |  |  |  |  |
|  |  |  |  |  |  |  |  |  |  |

| **Table S3.** List of *Bacteroides caccae* genomes used for the pan genome analysis. | | | | |
| --- | --- | --- | --- | --- |
| **Strain** | **BioSample** | **BioProject** | **Assembly** | **State** |
| ATCC 43185 | SAMN00627055 | PRJNA18163 | GCA_000169015.1 | Contig |
| CL03T12C61 | SAMN02463916 | PRJNA64801 | GCA_000273725.1 | Scaffold |
| 2789STDY5834946 | SAMEA3545409 | PRJEB10915 | GCA_001405515.1 | Scaffold |
| 2789STDY5834880 | SAMEA3545345 | PRJEB10915 | GCA_001405955.1 | Scaffold |
| ATCC 43185 | SAMN07340701 | PRJNA393727 | GCA_002222615.2 | Complete |
| ATCC 43185 | SAMN08570051 | PRJNA343143 | GCA_002959715.1 | Contig |
| AF24-29LB | SAMN09734473 | PRJNA482748 | GCA_003459065.1 | Scaffold |
| OF02-8 | SAMN09736685 | PRJNA482748 | GCA_003463115.1 | Scaffold |
| OF02-6LB | SAMN09736683 | PRJNA482748 | GCA_003463165.1 | Scaffold |
| AM31-16AC | SAMN09736428 | PRJNA482748 | GCA_003468785.1 | Scaffold |
| AM22-13LB | SAMN09734901 | PRJNA482748 | GCA_003470465.1 | Scaffold |
| AM26-6LB | SAMN09736340 | PRJNA482748 | GCA_003508775.1 | Scaffold |
| bj_0095 | SAMN10239563 | PRJNA496358 | GCA_004167345.1 | Contig |
| BSD2780061689st1_A4 | SAMN10863268 | PRJNA518912 | GCA_005844255.1 | Scaffold |
| BIOML-A14 | SAMN11943400 | PRJNA544527 | GCA_008416065.1 | Contig |
| BIOML-A10 | SAMN11943396 | PRJNA544527 | GCA_008416105.1 | Contig |
| BIOML-A7 | SAMN11943393 | PRJNA544527 | GCA_008416185.1 | Contig |
| BIOML-A6 | SAMN11943392 | PRJNA544527 | GCA_008416195.1 | Contig |
| BIOML-A5 | SAMN11943391 | PRJNA544527 | GCA_008416215.1 | Contig |
| BIOML-A4 | SAMN11943390 | PRJNA544527 | GCA_008416235.1 | Contig |
| BIOML-A3 | SAMN11943389 | PRJNA544527 | GCA_008416255.1 | Contig |
| BIOML-A2 | SAMN11943388 | PRJNA544527 | GCA_008416295.1 | Contig |
| BIOML-A1 | SAMN11943387 | PRJNA544527 | GCA_008416305.1 | Contig |
| BIOML-A32 | SAMN11943418 | PRJNA544527 | GCA_008572305.1 | Contig |
| BIOML-A30 | SAMN11943416 | PRJNA544527 | GCA_008572365.1 | Contig |
| BIOML-A29 | SAMN11943415 | PRJNA544527 | GCA_008572405.1 | Contig |
| BIOML-A28 | SAMN11943414 | PRJNA544527 | GCA_008572425.1 | Contig |
| BIOML-A27 | SAMN11943413 | PRJNA544527 | GCA_008572445.1 | Contig |
| BIOML-A26 | SAMN11943412 | PRJNA544527 | GCA_008572465.1 | Contig |
| BIOML-A25 | SAMN11943411 | PRJNA544527 | GCA_008572485.1 | Contig |
| BIOML-A24 | SAMN11943410 | PRJNA544527 | GCA_008572505.1 | Contig |
| BIOML-A23 | SAMN11943409 | PRJNA544527 | GCA_008572535.1 | Scaffold |
| BIOML-A22 | SAMN11943408 | PRJNA544527 | GCA_008572545.1 | Scaffold |
| BIOML-A21 | SAMN11943407 | PRJNA544527 | GCA_008572575.1 | Contig |
| BIOML-A19 | SAMN11943405 | PRJNA544527 | GCA_008572585.1 | Contig |
| BIOML-A18 | SAMN11943404 | PRJNA544527 | GCA_008572635.1 | Contig |
| BIOML-A16 | SAMN11943402 | PRJNA544527 | GCA_008572685.1 | Contig |
| 1001270H_150608_E1 | SAMN15532983 | PRJNA637878 | GCA_015546885.1 | Scaffold |
| BSD2780061689_150309_E2 | SAMN15532625 | PRJNA637878 | GCA_015549345.1 | Scaffold |
| D6t1_180914_E5 | SAMN15532734 | PRJNA637878 | GCA_015551865.1 | Scaffold |
| D43t1_170807_D2 | SAMN15533254 | PRJNA637878 | GCA_015553135.1 | Scaffold |
| J1101437_171009_H5 | SAMN15533324 | PRJNA637878 | GCA_015556775.1 | Scaffold |
| BSD2780120875b_170604_B11 | SAMN15532523 | PRJNA637878 | GCA_015557245.1 | Scaffold |
| D31t1_170403_E5 | SAMN15532371 | PRJNA637878 | GCA_015560545.1 | Scaffold |
| FDAARGOS_1097 | SAMN16357266 | PRJNA231221 | GCA_016726305.1 | Complete |
| FDAARGOS_1219 | SAMN16357361 | PRJNA231221 | GCA_016889745.1 | Complete |
| MGYG-HGUT-02549 | SAMEA5852054 | PRJEB33885 | GCA_902388185.1 | Contig |

**Table S4.** Metabolic reactions added to the *B. caccae* model.

| **Reaction Name** | **Reaction** | **Function** |
| --- | --- | --- |
| ARGabcIE | 'h2o[c] + atp[c] + arg_L[c] -> h[c] + pi[c] + adp[c] + arg_L[e]' | Arginine ABC* transporter (export) |
| EX_asp_L(e) | 'asp_L[e] <=>' | Aspartate Exchange reaction |
| 'AspTr' | 'ala_L[c] + asp_L[e] <=> asp_L[c] + ala_L[e]' | Alanine-Aspartate antitransporter (bidirectionnal) |
| 'EX_glu_L(e)' | 'glu_L[e] <=>' | Glutamate Exchange reaction |
| 'GLUT2r' | 'h[e] + glu_L[e] <=> h[c] + glu_L[c]' | Glutamate symport (bidirectionnal) |
| 'EX_gln_L(e)' | 'gln_L[e] <=>' | Glutamine Exchange reaction |
| 'GlnT' | 'h2o[c] + atp[c] + gln_L[e] -> h[c] + pi[c] + adp[c] + gln_L[c]' | Glutamine ABC* transporter (import) |
| 'GlnTex' | 'h2o[c] + atp[c] + gln_L[c] -> h[c] + pi[c] + adp[c] + gln_L[e]' | Glutamine ABC* transporter (export) |
| 'EX_gly_L(e)' | 'gly[e] <=>',' | Glycine Exchange reaction |
| 'tr-gly_L' | 'gly[e] -> gly[c]' | Glycine passive transporter (import) |
| 'GlyTex' | 'gly[c] -> gly_L[e]' | Glycine passive transporter (export) |
| 'EX_his_L(e)' | 'his_L[e] <=>' | Histidine Exchange reaction |
| 'HisP' | 'h2o[c] + atp[c] + his_L[e] -> h[c] + pi[c] + adp[c] + his_L[c]' | Histidine ABC* transporter (import) |
| 'EX_ile_L(e)' | 'ile_L[e] <=>' | Isoleucine Exchange reaction |
| 'LivI-isoleucine' | 'ile_L[e] -> ile_L[c]' | Isoleucine passive transporter (import) |
| 'IleTex' | 'ile_L[c] -> ile_L[e]' | Isoleucine passive transporter (export) |
| 'EX_phe_L(e)' | 'phe_L[e] <=>' | Phenylalanine Exchange reaction |
| 'PheT' | 'phe_L[e] -> phe_L[c]' | Phenylalanine passive transporter (import) |
| 'PheTex' | 'phe_L[c] -> phe_L[e]' | Phenylalanine passive transporter (export) |
| 'EX_trp_L(e)' | 'trp_L[e] <=>' | Tryptophane Exchange reaction |
| 'tr-trp_L' | 'trp_L[e] <=> trp_L[c]' | Tryptophane passive transporter (import) |
| 'EX_tyr_L(e)' | 'tyr_L[e] <=>' | Tyrosine Exchange reaction |
| 'TyrT' | 'tyr_L[e] -> tyr_L[c]' | Tyrosine passive transporter (import) |
| 'TyrTex' | 'tyr_L[c] -> tyr_L[e]' | Tyrosine passive transporter (export) |
| 'EX_val_L(e)' | 'val_L[e] <=>' | Valine Exchange reaction |
| 'LivI-valine' | 'val_L[e] -> val_L[c]' | Valine passive transporter (import) |
| 'ValTex' | 'val_L[c] -> val_L[e]' | Valine passive transporter (export) |
| 'GluT-4' | 'glc_D[e] + h[c] -> glc_D[c] + h[e]' | Glucose symport (import) |
| 'sglT' | 'na1[e] + glc_D[e] -> na1[c] + glc_D[c]' | Glucose secondary active transporter (import) |
| 'LacTex' | 'lac_D[c] -> lac_D[e]' | D-Lactate passive transporter (export) |
| 'PtrcTex' | 'ptrc[c] -> ptrc[e]' | Putrescine passive transporter (export) |
| 'EX_WheyProtein_e' | 'wheyprotein[e] <=>' | Whey Protein Exchange reaction |
| 'EX_MedProtein_e' | 'medprotein[e] <=>' | Medium Protein Exchange reaction |
| protease_rxn' | 1 wheyprotein[e] + 23 atp[c] -> 21 adp[c] + 8.3 ala_L[e] + 2.3 arg_L[e] + 13.1 asp_L[e] + 3.6 cys_L[e] + 18.4 glu_L[e] + 3.8 gly[e] + 2 his_L[e] + 6.5 ile_L[e] + 14.2 leu_L[e] + 10.3 lys_L[e] + 2.4 met_L[e] + 3.4 phe_L[e] + 5.9 pro_L[e] + 6.5 ser_L[e] + 6.5 thr_L[e] + 1.7 trp_L[e] + 3.1 tyr_L[e] + 7 val_L[e]' | Whey Protein Proteolysis reaction |
| med_protease_rxn' | 1 protein[e] + 23 atp[c] -> 21 adp[c] + 5.7 ala_L[e] + 0.8 arg_L[e] + 3.6 asp_L[e] + 6.6 glu_L[e] + 11.3 gly[e] + 0.7 his_L[e] + 2 ile_L[e] + 2.1 leu_L[e] + 0.9 lys_L[e] + 0.4 met_L[e] + 1.1 phe_L[e] + 6 pro_L[e] + 1.1 ser_L[e] + 0.8 thr_L[e] + 0.2 tyr_L[e] + 2.6 val_L[e]' | Medium Protein Proteolysis reaction |
| * : ATP-binding cassette | |  |

| **Table S5.** Description of the in silico media used to perform dynamicFBA | | | |
| --- | --- | --- | --- |
| **Metabolites** | **P20 (mM)** | **P2 (mM)** | **GLM (mM)** |
| 'EX_ala_L(e)' | 2.92134831500000 | 2.92134831500000 | 3.14606741600000 |
| 'EX_arg_L(e)' | 2.43295019200000 | 2.50957854400000 | 2.54789272000000 |
| 'EX_asn_L(e)' | 0.852130325800000 | 0.827067669200000 | 0.800000000000000 |
| 'EX_asp_L(e)' | 0.852130325800000 | 0.827067669200000 | 0.852130325800000 |
| 'EX_cys_L(e)' | 0.771349862300000 | 0.661157024800000 | 0.578512396700000 |
| 'EX_gln_L(e)' | 2 | 1.99546485300000 | 1.99546485300000 |
| 'EX_glu_L(e)' | 2.01814059000000 | 1.99546485300000 | 1.90000000000000 |
| 'EX_gly_L(e)' | 1.46666666700000 | 1.46666666700000 | 1.46666666700000 |
| 'EX_his_L(e)' | 0.387096774200000 | 0.387096774200000 | 0.387096774200000 |
| 'EX_ile_L(e)' | 0.941475827000000 | 0.992366412200000 | 0.992366412200000 |
| 'EX_leu_L(e)' | 2.23918575100000 | 2.31552162800000 | 2.34096692100000 |
| 'EX_lys_L(e)' | 1.59817351600000 | 1.66666666700000 | 1.66666666700000 |
| 'EX_met_L(e)' | 0.447427293100000 | 0.469798657700000 | 0.469798657700000 |
| 'EX_phe_L(e)' | 1.15151515200000 | 1.17171717200000 | 1.21212121200000 |
| 'EX_pro_L(e)' | 0.521739130400000 | 0.521739130400000 | 0.521739130400000 |
| 'EX_ser_L(e)' | 1.11111111100000 | 1.17460317500000 | 1.23809523800000 |
| 'EX_thr_L(e)' | 0.728291316500000 | 0.840336134500000 | 0.840336134500000 |
| 'EX_tyr_L(e)' | 0.386740331500000 | 0.386740331500000 | 0.405156537800000 |
| 'EX_val_L(e)' | 1.25356125400000 | 1.31054131100000 | 1.31054131100000 |
| 'EX_glc_D(e)' | 29 | 29 | 29 |
| 'EX_ac(e)' | 0.888888888900000 | 1.05555555600000 | 0.944444444400000 |
| 'EX_ppa(e)' | 0.315315315300000 | 0.495495495500000 | 0.540540540500000 |
| 'EX_lac_D(e)' | 0.481481481500000 | 0.555555555600000 | 0.592592592600000 |
| 'EX_lac_L(e)' | 0 | 0 | 0 |
| 'EX_lcts(e)' | 1.06237816800000 | 0.555555555600000 | 0.477582846000000 |
| 'EX_succ(e)' | 7.48587570600000 | 7.82485875700000 | 7.90960452000000 |
| 'EX_WheyProtein_e' | 1.02850000000000 | 0.0500000000000000 | 0 |
| 'EX_MedProtein_e' | 0.513000000000000 | 0.528988573800000 | 0.513000000000000 |
