# Supplementary material for: Design of a proteolytic module for improved metabolic modeling of Bacteroides caccae
Source: mSystems. 2024 Mar 22;9(4):e00153-24. doi: 10.1128/msystems.00153-24 (PMC11019848; doi:10.1128/msystems.00153-24)
Supplement: Table S2 — Protease in B. caccae ATCC 43185 genome. [file msystems.00153-24-s0003.docx]

**Table S2.** Protease in *B. caccae ATCC 43185* genome

| Sup.Fam. | Sub Fam. | Prot. Name | Orga. | MEROPS description | Sequence | Original Annotation | Signal peptide | | T domain | | Localisation prediction | | E-value  -≤ 0.05 | Bitscore  > 50 | Qcovs  > 75 % | % ID  > 30 % | Pangenome | |
| --- | --- | --- | --- | --- | --- | --- | --- | --- | --- | --- | --- | --- | --- | --- | --- | --- | --- | --- |
|  |  |  |  |  |  |  | SignalP V6 | Phobius | TM HMM | Phobius | PsortB | Pred.  Loc. |  |  |  |  | Nber of hits | class of gene |
| **Antibiotic Resistance** | | | | | | | | | | | | | | | | | | |
| M56 | M56.UPW | UP | *B. thetaiotaomicron* | The peptidases of family M56 allow bacteria to respond to the presence of beta-lactam antibiotics by the expression of beta-lactamases and penicillin-binding proteins. | MGDLLFYLLKSGCYLIIFYLLFKLLMSGTTFFRFNRVTILAGIIGCMFLPLIEFTTQEETFLTVPLQTIQEIFVEQADGIFWDTMFWVRPQVDETGNMQAINWCPIALGYVYLAGGLFVLCRILLSFYRMFQLIRNGKRRSYGKYKLIVVSEPISSFCWGKYIVVSVSDYSQQSTDGILLHETMHLRYRHTLDLLCMQCLLILYWFNPAIWLLKRELQEVHEFEADNGVLNTGIDATRYQLLLVKKAVGTRLYSMANGFNHSKLKKRITMMLKERTNRWARLKLLFVVPVMGGALYAFAQPEVKEDLGLNTVREVLQSKTGEDQDIFKFVDDEMEAYYNRSPKNAELKYKIRERQTHIVKVDANGQLTLNNKTIAKEVLKEVLKKTMVEHKRLAKEKYNSAEDQVGGILFSPDTPKEQVKDLLSVMRDAFVEIRADIAQTSGNNSKEYLDKEFPILIGYGDISIKDRKKNPYNNSTTMKITGIHLKLLSAAGKVEKEITNFTLAELERDLKDFCTLQGDKKNEITVSMKVDKGCEMGIVTDVKTVLRNNYLLKLNYQ | Hyp. prot. | N | N | 4 | 6 | Cyto. Membr. | M | 4.2E-170 | 494 | 99 | 47.857 | 1 | Cloud |
| M56 | M56.UPW | UP | *B. thetaiotaomicron* |  | MTPELAYFLKINVAIALFYAFYRLFFHKDTFFHWRRIALLCFFAISLLYPLLNIEGWIKAHEPMVAMADLYATIILPEQVVTIPQETEINWQELIKLLTGIIYWSGVLLLTARFFLQLGSIMRLHFQCSKSQLKGVRVHLLKKEAGPFSFFHWIFIHPQSHTDSEISEIITHEETHARQYHSIDVLISEIMCIFCWFNPFSWLMKREVRGNLEYMADSRVLETGHDSKSYQYHLLGLAHHKAAANLSNSFNVLPLKNRIKMMNKRRTKEIGRTKYIMFLPLAALLMIVSNIEVVARTTERFAKEVMEQVNTPQPEIANIPELQSEEIQQTVALPAKKEIAKSTPIAQSKSIPDSVVFEVVEVMPEFPGGQQALMQYLAKNIKYPVTAHENGKQGRVIVSFVVKKDGNISDIKVARSVDPYLDKEAVRVIAAMPQWKPGKQRGENVNVRFSVPVMFRLQGPAPSKGKEIKQADLEEVVVVAYGPKEDSNANATDAKSGTALKVAEVMPKFPGGIPGLMQYLARNIKYPTIAQKNKEQGRVILQMIVGKDGSISNIKVLRSISPLLDAEAIRVVSTMPKWEPGQQGGQAIAVEYTLPIVFKLQ | cell envelope biogenesis protein TonB | N | N | 4 | 4 | Cyto. Membr. | M | 0 | 930 | 100 | 73.214 | 7 | Cloud |
| **Bacteriocin** | | | | | | | | | | | | | | | | | | |
| C39 | C39.001 | bacteriocin-processing peptidase | *Clostridium perfringens* | Cleavage of leader peptide of bacteriocins during export from the organism. | MIELKELTLGYGQRTLLKTVNARITGGQLVALLGRNGTGKSTLLRAIMGLESPQSGEINLQGKDIASLKPEKLALSISFVTTDKVRIANLRCKDVVALGRAPYTNWLGQLQPEDRKIVDNAIQLVGMSGYAEKTMDKMSDGECQRIMIARALAQDTPVILLDEPTAFLDLPNRYELCLLLKKLAQEEGKCILFSTHDLDIALSLCDSIMLIDNPYMYTLPTSEMITSGHIERLFRNESVTFDAQEMRVRIKSV | iron ABC transporter ATP-binding protein | N | N | 0 | 0 | Cyto. | Ic | 1.39E-18 | 85,5 | 92 | 30.769 | 47 | Core |
| C39 | C39.001 | bacteriocin-processing peptidase | *Spirochaeta africana* |  | MIKLVGINKIYRTNEIETVALEDVNLTVNKGEFLSIMGPSGCGKSTLLNIMGLLDTPTGGSIEVAGTRIESMKDKELAAFRNKKLGFVFQSFHLINSLNVLDNVELPLLYRRMSSSERKRLAQDVLAKVGLSHRMRHFPSQLSGGQCQRVAVARAIIGNPEIILADEPTGNLDSRMGAEVMELLHRLNKEDGRTIVMVTHNEEQAKQTSRTVRFFDGHQVQ | macrolide ABC transporter ATP-binding protein | N | N | 0 | 0 | Cyto. Membr. | Ic | 1.47E-24 | 102 | 98 | 30.769 | / | Cloud* |
| C39 | C39.001 | bacteriocin-processing peptidase | *Spirochaeta africana* |  | MEESKMVLRTEDLVKKYGKRTVVSHVSIDVKQGEIVGLLGPNGAGKTTSFYMTVGLITPNEGRIFLDDLEITKYPVYKRAQTGIGYLAQEASVFRQMSVEDNIASVLEMTNKPKEYQKEKLESLIAEFRLQKVRKNKGNQLSGGERRRTEIARCLAIDPKFIMLDEPFAGVDPIAVEDIQQIVWKLKDRNIGILITDHNVQETLSITDRAYLLFEGKILFQGTPEELSENQIVREKYLSNSFVLRRKDFQLEK | ABC transporter ATP-binding protein | N | N | 0 | 0 | Cyto. | Ic | 3.36E-23 | 99 | 84 | 31.193 | 47 | Core |
| C39 | C39.UNW | Non-peptidase homologues | *Mannheimia cf. haemolytica* | Bacteriocins are antibiotic proteins secreted by some species of bacteria that inhibit the growth of other bacterial species. The bacteriocin is synthesized as a precursor with an N-terminal leader peptide, and processing involves removal of the leader peptide by cleavage at a Gly-Gly bond, followed by translocation of the mature bacteriocin across the cytoplasmic membrane. The endopeptidases of family C39 serve both functions, and the proteins are known as ATP-binding cassette transporters (ABC transporters). | MEEALIQYKNVEIHQQELCVLSGVNLELHKGEFVYLIGKVGSGKTSLLKTLYGELDVIDGDAEVLGYNMRSIKRKHIPQLRRKLGIVFQDFQLLTDRTVYNNLEFVLRATGWKNKQEIKERIEEVLDLVGMSNKGYKIPNELSGGEQQRIVIARAVLNSPAIILADEPTGNLDVETGKSIVELLHNICETGSSVVMTTHNLQLLKEYPGRVYRCAEHHITDVTDEYMPRQRTIEIDLNIDN | phosphonate ABC transporter ATP-binding protein | N | N | 0 | 0 | Cyto. Membr. | Ic | 2.89E-20 | 90,1 | 83 | 32.212 | 47 | Core |
| C39 | C39.UNW | Non-peptidase homologues | *Nitrobacter hamburgensis* |  | MDTVKIDARDVNFWYGDFHALKGISMQIEEKSVVAFIGPSGCGKSTFLRLFNRMNDLIPATRLEGEIRIDGQNIYAKGVKVDELRKNVGMVFQRPNPFPKSIFENVAYGLRVNGVKDNAFIRQRVEETLKGAALWDEVKDKLKESAYALSGGQQQRLCIARAMAVSPSVLLMDEPASALDPISTAKVEELIHELKKDYTIVIVTHNMQQAARVSDKTAFFYMGEMVEFDNTKKIFTNPEKEATQNYITGRFG | phosphate ABC transporter ATP-binding protein | N | N | 0 | 0 | Cyto. Membr. | Ic | 2.74E-24 | 102 | 86 | 32.759 | 26 | Shell |
| C39 | C39.UNW | Non-peptidase homologues | *Saccharophagus degradans* |  | MIKLEGITKSFGSLQVLKGIDLEINKGEIVSIVGPSGAGKTTLLQIMGTLDEPDAGNVQIDGTVVSRMKEKELSAFRNKNIGFVFQFHQLLPEFTALENVMIPAFIAGVSSKEANEHAIRILDFMGLAERASHKPNELSGGEKQRVAVARALINDPAVILADEPSGSLDTHNKEDLHQLFFDLRERLGQTFVIVTHDEGLSKITDRTIHMVDGMIKKD | ABC transporter ATP-binding protein | N | N | 0 | 0 | Cyto. Membr. | Ic | 4.7E-24 | 100 | 93 | 31.776 | 47 | Core |
| C39 | C39.UNW | Non-peptidase homologues | *Synechococcus elongatus* |  | MKKVIELQNIKRDFQVGEETVHALRGVSFTINEGEFVTIMGTSGSGKSTLLNTLGCLDTPTSGEYLLDGISVRTMSKPQRAVLRNRKIGFVFQSYNLLPKTTAVENVELPLMYNSAVSASERRRRAIESLQAVGLGDRLEHKSNQMSGGQMQRVAIARALVNNPAVILADEATGNLDTRTSFEILVLFQKLHAEGRTIIFVTHNPEIAQYSSRNIRLRDGHITEDTVNTKILSAAEALAALPKNEED | macrolide ABC transporter ATP-binding protein | N | N | 0 | 0 | Cyto. Membr. | Ic | 8.53E-28 | 112 | 89 | 35.268 | 5 | Cloud |
| C39 | C39.004 | Lantibiotic processing peptidase | *Bacillus_halodurans* |  | MIKTEKLTKLFATEEVQTKALNEVTLQVEQGEFVAIMGPSGCGKSTLLNILGTLDSPTSGSYFFEGKQVDKMNENQLTALRKGNLGFIFQSFNLIDELTVCENVELPLIYIGVKANERKERVKKVLEKVNLLHRANHYPQQLSGGQQQRVAIARAVVTDCKLLLADEPTGNLDSVNGIEVMELLSELNAQGTTIIIVTHSQRDAMYAHRVIQLLDGQIVAENVNRPLGKNMSAKNETV | phosphonate ABC transporter ATP-binding protein | N | N | 0 | 0 | Cyto. Membr. | Ic | 4.39E-21 | 92,4 | 88 | 32.258 | 12 | Shell |
| C39 | C39.005 | colicin V processing peptidase | *Chromobacterium violaceum* |  | MIKTINLQKIFKTEEVETWALNNVSIEVKQGEFVAIMGPSGCGKSTLLNILGLLDNPTGGEYYLNGMEVSKYTESQRTNLRKGVIGFVFQSFNLIDELNVYENIELPLLYMGISASERKKRVETAMERMAITHRSKHFPQQLSGGQQQRVAIARAVVANPKLILADEPTGNLDSKNGKEVMGLLSELNKEGTTIVMVTHSQHDAGYADRVINLFDGQVVTEVSM | phosphonate ABC transporter ATP-binding protein | N | N | 0 | 0 | Cyto. Membr. | Ic | 8.25E-23 | 97,1 | 93 | 31.628 | 47 | Core |
| C39 | C39.004 | Lantibiotic processing peptidase | *Bacillus licheniformis* |  | MIEVKGLYKSFDDKTVLSDINATFENGKTNLIIGQSGSGKTVLMKCIVGLLTPEKGEVLYDGRNLVMMGKKEKKMLRKEMGMIFQSAALFDSLSVLDNVMFPLNMFSNDTLRERTKRAMFCLDRVNLTEAKDKFPGEISGGMQKRVAIARAIALNPQYLFCDEPNSGLDPKTSLVIDDLIHDITQEYNMTTIINTHDMNSVLGIGEKVIYIYEGHKEWEGTKDDIFTSTNERLNNFIFASDLLRKVKDLEVQSMEG | ABC transporter ATP-binding protein | N | N | 0 | 0 | Cyto. Membr. | Ic | 4.43E-23 | 98,6 | 94 | 32.258 | 47 | Core |
| C39 | C39.004 | Lantibiotic processing peptidase | *Streptococcus salivarius* |  | MIQLTDINKTYNNGAPLHVLKGINLTIGRGEFVSIMGASGSGKSTLLNILGILDNYDTGDYYLNNVLIKDLSETKAAEYRNRMIGFIFQSFNLISFKDAVENVALPLFYQGISRKKRNALALEYLDRLGLKDWAHHMPNEMSGGQKQRVAIARALITQPQIILADEPTGALDSKTSVEVMQILKDLHKMGMTIVVVTHESGVANQTDKIIHIKDGIIERIEDNIDHDASPFGKNGIMK | ABC transporter ATP-binding protein | N | N | 0 | 0 | Cyto. Membr. | Ic | 1.07E-24 | 102 | 91 | 35.088 | 47 | Core |
| C39 | C39.005 | colicin V processing peptidase | *Shewanella oneidensis* |  | MMIELQNVGFAYNNKHTVFSNLNLYIDRGDIVSIVGNSGCGKTTLLNLIAGVLLPSKGEIKKSDKNIAYLMQDVTLLPYRTAWENTFLACELRGIPVDNIQKQAAKEILDLFNIETEALNKFPKELSGGMKQRIGLLQTLLTDASLFLLDEPFNAIDINALNSIKLYIWEYLIKSNKTMIFITHNIDQALLLSDRILIMRSPTELLEIKPTKEYCSLSPDERIDTSEYKKLFFEIVENLKYEK | Hyp. prot. | N | N | 0 | 0 | Cyto. Membr. | Ic | 5.7E-19 | 86,3 | 81 | 33.641 | 44 | SoftCore |
| C39 | C39.005 | colicin V processing peptidase | *Chromobacterium violaceum* |  | MINTEIINFLSEVDTNVFLFFNGIHSPFWDYFMSAFTGKIIWVPMYATILYILLINFHWKVVLCYVAAIALTITFADQMCSSIIRPVVARLRPANPENPIVDLVHIVNGYRGGSYGFPSCHAANSFGLAMYVVFLFRKRWLSVFIITWAVLNCYTRIYLGVHYPGDLIVGGIIGGFGGWLFCTIAHKIASYLQKPSRVRRNTIRQTSVTMYVGFLTVLGIIIYSTIKSW | phospholipid phosphatase | N | N | 6 | 6 | Cyto. Membr. | M | 8.25E-23 | 97,1 | * | 31.628 | 1 | Cloud |
| C39 | C39.UNW | Non-peptidase homologues | *Pseudomonas syringae* |  | MKEFLQLMRRFVSPYKKYIGWAVLLNILSAVFNVFSFTFLIPILSILFKTEGADKVYHFMEWGSGDLADVAKNNFYYYISQMIIDNGPTMALIFLGLFLMIMTLFKTGCYFASSAVMIPLRTGVVRDIRIMVYAKVMRLPMSFFSEERKGDIIARMSGDVGEVENSITSSLDMLMKSPIMIILYFATLVITSWQLTLFTIVVLPGMGWLMGVVGRKLKRQSLEAQSKWSDTMSQLEETLGGLRIIKAFIAEDKMINRFTKCSNELRDATNKVAIRQAMAHPMSEFLGTILIVAVLWFGGTLILGQNASIDAPTFIFYMVILYSVINPLKDFAKAGYNIPKGLASMERVDKILKAENKIKEISNPKPLKGLNDKIEFKDITFSYDGKREVLKHVNLTVPKGQTIALVGQSGSGKSTLVDLLPRYHDVQEGDITIDGTSIRDVRIADLRSLIGNVNQEAILFNDTFFNNIAFGVENATMEQVIEAAKIANAHDFIMEKPEGYNMNIGDRGGKLSGGQRQRISIARAILKNPPILILDEATSALDTESERLVQEALERLMKTRTTIAIAHRLSTIKNADEICVLYEGEIVERGKHEELIELNGYYKRLHDMQQL | antibiotic ABC transporter ATP-binding protein | N | N | 6 | 6 | Cyto. Membr. | M | 1.57E-89 | 293 | 88 | 33.883 | 47 | Core |
| **Cell Wall** | | | | | | | | | | | | | | | | | | |
| M23B | M23.UNB | Non-peptidase homologues | *B. thetaiotaomicron* | Peptidases of family M23 are used by certain bacteria to lyse cell walls of other bacteria, either as a defensive or feeding mechanism. The soil bacterium Lysobacter enzymogenes is also capable of lysing soil nematodes. The peptidases are synthesized as precursors and are activated extracellularly. Lysostaphin is bound to the cell wall at its C-terminus (Baba & Schneewind, 1996) | MMKHFFVILIGCLWLAIPLFAQSNKLIRELESKRGALQKQISETESILKDTKKDVGSQLNSLAVLTGQIEERKRYIIAINNDVEAIERELTSLQRQLNGLQKDLKDKKKKYEASVQYLYKNKSIEEKLMFIFSAKNLGQTYRRMRYVREYATYQRLQGEEILKKQEQIRKKKVEREQVKAAKESLLKEREGEKTKLEAQEKEKRTLVANLQKKQRGLQGEINKKRREANQLNARIDKLIAEEIERARKRAEEEARREAAARKKAEGKESQTAGTGTTVKTNSKPLETYTMSKADRELSGNFAANRGKLPMPISGAYIITSHYGQYAVEGLRNVKLDNKGIDIQGKPGAQARAIFDGKVAAVFQLNGLFNVLIRHGNYISVYCNLSSASVKSGDMVKTKQSIGQVFSDGTDNGRTVLHFQLRREKEKLNPEPWLNR | peptidase M23 | Y (SP) | Y | 0 | 0 | Unknown | Exp. | 0 | 706 | 100 | 87.126 | 47 | Core |
| M23B | M23.UPB | UP | *B. thetaiotaomicron* |  | MRRYITTLMLACCIGGYGQEKKQATFVPPFDFPLTLSGNFGEIRSNHFHGGLDFKTGGVIGKPVRALAEGYISRIRVTNGSGYVLDVCYHNGYSTINRHLSGFISPIAERVEKLQYENENWEVEIIPEPDEYPVKAGQQIAWSGNTGYSFGPHLHLDVFETETGDYVDPMPFFKTKIKDTRAPKADGIMFFPQLGKGVVDGKQENKMILPNTGRPVEAWGVIGTGIKAYDYMDGVSNHYGVYSVVLTVDEKEVFRSTVDRFSQEENRMINSWTCGQYMKSFIEPGNTLRLLKASNTNRGLVTIDEERDYRFLYTLKDVFGNTSKYSFTVRGRKQPLEPLQHREKYFFAWDKVNYLQEPGLNLVVPKGMLYDDAPLNYQVKADSGAVAFTYQLNDKPIPLHASCELRIGLRRKPITDTTKYYVVCVTPRGGKYSVGGKYEDGYMKASIRELGTYTVAIDTIPPEIIPVNKKQWGRTGKIVYRLKDKGTGIASYRGTIDGEYALFGRPNIVKSYWECVLDPKRVKKGGKHIVEFTVTDYCGNETVSRESFIW | peptidase M23 | Y (SP) | Y | 0 | 0 | Cyto. | Exp. | 0 | 1021 | 100 | 87.455 | 1 | Cloud |
| M15D | M15.011 | vanX D-Ala-D-Ala dipeptidase | *B. thetaiotaomicron* | The peptidases of family M15 are involved in bacterial cell wall biosynthesis and metabolism. They include zinc-dependent D-Ala-D-Ala carboxypeptidases and dipeptidases, and bacteriophage endolysins. The structure of the peptidoglycan polymer that constitutes the Streptomyces cell wall is stabilised by a cross-linking peptide that contains D-amino acids. The cross-linking peptide is synthesized as a precursor with an additional C-terminal D-Ala residue. The removal of the C-terminal D-Ala by the carboxypeptidase prepares the precursor for incorporation into the cell wall (a transpeptidation reaction catalysed by a serine-type D-Ala-D-Ala transpeptidase, S11.004). Vancomycin-resistant enterococci are pathogenic bacteria that attenuate antibiotic sensitivity by producing peptidoglycan precursors that terminate in D-Ala-D-lactate rather than D-Ala-D-Ala. A key enzyme in antibiotic resistance is the metallodipeptidase VanX that reduces the cellular pool of the D-Ala-D-Ala dipeptide so that only the resistant D-Ala-D-lactate is incorporated into the cell wall (Lessard & Walsh, 1999). | MRFLKYSLPVLCLLLAECTSVSGHKEKERLTMAEYKHPSDDMQPREECSPAPQPKKSAMALYMDSLGLVNIAELDNSITVKLMYTQADNFTGEVLYDDLSEAYLHPDAAYALVKAQEALKQLHPSYNLVVYDAARPMSVQKKMWNVVKGTPKYKYVSNPNRGGGLHNYGLAVDINIQDSLGQPLPMGTKVDHLGIEAHITQESELVRNGKISEAERQNRILLRRVMKEAGFRALPSEWWHFNFCSRDVARQKYKVIP | D-alanyl-D-alanine dipeptidase | Y (Lipo) | Y | 0 | 0 | Cyto. | Exp. | 2.2E-138 | 391 | 100 | 66.438 | 47 | Core |
| M15A | M15.UNA | Non-peptidase homologues | *B. fragilis* |  | MGKYFTIAEMVKSETADRCGIDNRLPKSLICNVNGLIDNVLDPLREAYGKPVTVTSGYRCEVLNKAVGGSKTSEHMKGMAADIVGTPNTKEENKRLFNLIQELEIPFTQLIDEKNFSWVHVSYDSCNVKKQVLKL | peptidase M15 | N | N | 0 | 0 | Unknown | Ic | 1.57E-47 | 150 | 100 | 54.815 | 1 | Cloud |
| S66 | S66.UPW | UP | *B. thetaiotaomicron* | LD-carboxypeptidase substrates are produced when bacterial cell walls are degraded, and it is thought that the LD-carboxypeptidase is required to allow murein tetrapeptide fragments to be reincorporated into murein. The tetrapeptides are truncated to the tripeptides, which can then be reconverted into peptidoglycan building blocks by the attachment of preformed D-Ala-D-Ala dipeptides (Korza & Bochtler, 2005). A second member of family S66 in Escherichia coli is the mccF gene product (S66.003) that contributes to self-immunity to microcin C7 (Gonzalez-Pastor et al., 1995). | MDIQFPPFLQKGDKVVIVSPSSKIDKQFLKGAKKRIESWGVKVVMGKHAGGSSGRYAGTISQRLKDLQDAMDDPKVKAILSSRGGYGAVHLIDKIDFTAFREYPKWLLGFSDITALHNLFQKNGYASLHSLMARHLAVEPEDDPCTNYLKDILFGNLPSYTCEKHKLNKQGSTQGVLRGGNMAVAYGLRGTPYDIPAEGTVLFIEDVSERPHAIERMMYNLKLGGVLEKLSGLIVGQFTEYEEDCSLGKELYAALADLVKEYDYPVCFNFPVGHVTHNLPLINGAKVEFTVGKKNVELKFIC | peptidase U61 | N | N | 0 | 0 | Cyto. | Ic | 0 | 573 | 100 | 89.404 | 47 | Core |
| M50B | M50.004 | RseP peptidase | B. thetaiotaomicron | RseP peptidase also cleaves transmembrane sequences (Akiyama et al., 2004), and it now seems very likely that intramembrane proteolysis (reviewed by Weihofen & Martoglio, 2003) is a general activity of peptidases in family M50. | METFLIRALQLIMSLSLLVIIHEGGHFLFARLFKVRVEKFCLFFDPWFTLFKFKPKKSETEYAVGWLPLGGYVKIAGMIDESMDTEQMKQPEQPWEFRSKPAWQRLLIMVGGVLFNFLLALFIYSMILFAWGDQYIKVQEAPLGMDFNETAKSVGFKDGDVLLSADGVPFERYDGDMLSQIADAREVSVLRDGAKASVYIPDDMMQRLLADSIRFASFRFPYVVDSVMVNSPAAQAGIQPGDSIIALNGTPISFSDFKQAMAERKKNAATLLKDSIDPRFITLAYVRGGVTDTLSMRVDSAYLMGVTACLVTDRLLPMVKKQYAFLESFPAGVSLGVKTLKGYVGNMKYLFSKEGAKQLGGFGTIGSIFPATWDWHQFWYMTAFLSIILAFMNILPIPALDGGHVLFLIYEMIARRKPSDKFMEYAQMTGMILLFGLLIWANFNDILRFFF | RIP metalloprotease RseP | N | N | 4 | 4 | Cyto. Membr. | M | 0 | 867 | 100 | 93.126 | 2 | Cloud |
| A08 | A08.001 | signal peptidase II | *B. ovatus* | Signal peptidase II removes the signal peptide from the N-terminus of the murein prolipoprotein, an essential step in the production of the bacterial cell wall. Homologues are known from nearly every bacterial genome so far completely sequenced. A few bacteria, including Pseudomonas fluorescens and Staphylococcus epidermidis, contain two family A8 homologues. | MKKLFTKGTIALIVIFSVLIIDQIIKIWIKTHMYWHESIRVTDWFYIYFTENNGMAFGMEIFGKLFLTTFRIVAVALIGWYLYKIVKKGFKTGYIVCVALILTGALGNIIDSVFYGVIFNESTHSQIASFMPEGGGYSTWFYGKVVDMFYFPIIDTNWPAWLPFVGGEHFIFFSPIFNFADAAISCGIIALLLFYSKYLNESYHSLDKGDKKEVENHEK | lipoprotein signal peptidase | N | N | 4 | 4 | Cyto. Membr. | M | 3,00E-153 | 424 | 100 | 95.434 | 47 | Core |
| M41 | M41.UPW | UP | *B. thetaiotaomicron* | The physiological substrates of peptidases in family M41 are generally membrane proteins. Substrates of FtsH peptidase in Escherichia coli include the proteins sigma32 (Okuno et al., 2004), YccA and SecY (Kihara et al., 1999). | MDNNSSNKNNSNKPNNKVNMPKFNLNWMYMIIALMLLGLWWGSDSRGAGNKAVTYSEFQDYVKNGYVSKVLGYEDKSIEAYLKPNSVGAVFGADSTKVGRNPIITSRAPSTDKLEEFLQAEKEAGHFDGTSDYPPKSDIFPAILIQVLPLVLLIALWIFFMRRMSGGGSGGPGGVFNVGKSKAQLFEKGGAIKITFKDVAGLAEAKQEVEEIVEFLKEPQKYTDLGGKIPKGALLVGPPGTGKTLLAKAVAGEANVPFFSLAGSDFVEMFVGVGASRVRDLFKQAKEKAPCIVFIDEIDAVGRARGKNPAMGGNDERENTLNQLLTEMDGFGSNSGVIILAATNRVDVLDKALLRAGRFDRQIHVDLPDLNERKEVFGVHLRPIKIDDTVDVDLLARQTPGFSGADIANVCNEAALIAARHGKKFVGKQDFLDAVDRIIGGLEKKTKITTEAERRSIALHEAGHASISWLLEYANPLIKVTIVPRGRALGAAWYLPEERQITTKEQMLDEMCATLGGRAAEDLFLNRISTGAMNDLERVTKQAYGMIAYLGMSDKLPNLCYYNNEEYSFNRPYSEKTAELIDTEVKNMVNEQYERAKKILSEHREGHNQLAQLLIDKEVIFAEDVERIFGKRPWASRSEEIMAAKESQEAAEAEKRLAEEVKEKEKEIKEEEAENSTEEKEATEAKITVEGKVSIEKKVISAEGGKKTT | diaminopimelate epimerase | N | N | 2 | 2 | Cyto. Membr. | M | 0 | 1235 | 96 | 90.896 | 47 | Core |
| S13 | S13.UPW | UP | *B. thetaiotaomicron* | PBP4 is Per.ic, loosely attached to the membrane. It is important both in the biosynthesis and turnover of the bacterial cell wall crosslinks; it is, however, not essential and deletion mutants are able to grow normally (Wilkin, 2004). | MKKVLLLVALSVCLLPLWGQRDFSQIDSLIKKMLPEASEVGISVYDLTAKRPLYSYRADKLSRPASTMKLLMAITALSRPDADEPFRTEVWHDGVIEHDTLQGNLYVVGGFDPEFDSQAMDSLIEEVMTFPFSVINGQVYGDVSMKDSLYWGHGWAWDDTPAGYQPYLSPLMFCKGTVQISVFPSAVQGDTASISCKPVSSYYTLTNRTKTRTSSAGKFSFSRDWLRNGNNLVVSGNVTSIRKDDINIYDSSAFFMHTFLERLRGKGITAPQSYGFAELPRDSVQVERIACWNTSVQEVLNQLMKESDNLNAEAFLCRLGAQATGKKQVAAEDGIVEIMQLIRQLGHNPKEYKIADGCGLSNYNYLSPALLVDFLKYAYSRTEVFRMLYKSLPVGGIDGTLKNRMKSAATFRNVHAKTGSFTAINTLAGYLKMKNGHEVAFAIMNQNVLSAAKARAFQDKVCEVIIGR | peptidase M15 | Y (SP) | Y | 0 | 0 | Per.ic | Exp. | 0 | 810 | 100 | 81.064 | 47 | Core |
| S12 | S12.UPW | UP | *B. thetaiotaomicron* | D-Ala-D-Ala carboxypeptidase B is involved in the synthesis and remodelling of bacterial cell walls. | MRIFPFILVVLFFILAGSVSSPAQNAASVEPLLLYKAQQDKNCRHWVDSVMDKLSFKEKVGQLFIYTIAPVNTKRNLELLREAIDTYKVGGLLFSGGKMQNQVELTNRAQRQAKVPVMITFDGEWGLAMRLRGTPVFPRNMVLGCIRDNRLIYEYGREVARQCRQIGVQVNFAPVADVNINPKNPVINTRSFGEDPIQVADKVIAYASGLEGGGVLSVCKHFPGHGDTDVDSHKALPVLPFTRERLDSVELYPFKEAIRAGVGGMMVGHLQVPVIEPIGGLPSSLSRNVVYDLLTDELAFKGLIFTDALAMRGVSGNGNVSLQALQAGNDMVLAPRNLKAEVPAVLAAVEKGELSREDIESKCRKVLTYKYALGLSKKKYVQLSGLEQRVNSPQARDLVRRLNLAAITVLNNKDHVLPLHTDKDQKIALLEVGDPGETEALAKQMTRYASLVRFRLRPKQTEEENRRLYDSLAIYKRVVVAVSEQRLASYQPFFTKFAPDAPVVYLFFTPGKMMLQIQRAVSHASAVVLAHSHNTDIQRQVADVLFAKATADGRLSASLGGLFHTGAGVTITPKTPLHFVPEEYGLSSVSLKRIDSVALDGIRQGAYPGCQVVVMKNGHVMVDKAFGTHTGKGSARVESSDIYDLASLTKTTATLLAVMKLYDKGRFNLTDKISDYLPFLQRTNKKDITIQEILYHQSGLPSWLPFYQEVIDKDSYKGRLFSARRDAQHPVNIGINTWANPNFKFKEEYVSPTKTGDYTIQICDNLWLNRSFRKVIEEKIVEAPLGQKRYVYSDIGFILLGMLVEQLAGMPMEVYLQSEFYEPLGLERTGYLPLRRLAKSEVIPSNNDRFLRKDTLQGFVHDEASAFFGGLAGNAGLFSTAREVARVYQMLLNGGEIDGRRYLSKETCQLFTTSVSKISRRGLGFDKPDREDSKKGNCAPDAPAEVYGHTGFTGTCAWVDPVNELVYVFLSNRIYPDVTNRKLIRLHIRERIQEAIYDAMKKK | beta-N-acetylglucosaminidase | Y (Lipo) | Y | 0 | 0 | Per.ic | Exp. | 0 | 1657 | 100 | 77.656 | 47 | Core |
| S12 | S12.UPW | UP | *Koribacter versatilis* |  | MRNRTSLILFFLFVTVFPECISGKVVAGAGRFEQYLPFIQGKRVGMVVNHTSVVGTGQTHLLDTLLKQHINVVKVFAPEHGFRGNADAGETVKDGKDSRTGVSIVSLYGNNKKPTAAQLKDIDVILFDIQDVGARFYTYISTMYYVMEACAENNKEMIVLDRPNPCDYVEGPILKPAYRSFVGMLPIPVLHGCTIGELARMINGEGWIAHKKNPCSLKVIPATGWIHGEPYSLPIKPSPNLPNDQSIRLYASLCPFEATSVSVGRGTTFPFQVLGAPNKKYGDFTFTPRSLPGFDKNPMHKNVVCYGEDLRNADDVNGFTLRYFLHFYRLSGEGAAFFSRARWFDLLMGTDSVRKAILRGDSEETIRNSWQKELQDYKKMRNKYLLYE | Hyp. prot. | Y (SP) | Y | 0 | 0 | Cyto. | Exp. | 7.1E-52 | 185 | 89 | 35.04 | 1 | Cloud |
| S12 | S12.UPW | UP | *B. fragilis* |  | MKKIFFLVVILTLSLLCRAQERKYSTFYYQRATLFEELPVTSSDIIFLGNSITNGAEWAELFKNKHVKNRGISGDICMGVYDRLDAILKGKPAKIFLLIGINDVSRGTPADTIVSRIEMIVRKIKADSPKTKLYLQSVLPVTDHYNMFKGHTSRWQVIPEINKGLVGLAEKEGATYIDLYSHFIDKQTGKMNTTYTNDGLHLLGKGYLKWVEIVKPYIGKK | sialate O-acetylesterase | Y (SP) | Y | 0 | 0 | Unknown | Exp. | 7.34E-47 | 164 | 88 | 40.609 | 4 | Cloud |
| C40 | C40.004 | spr peptidase | *B. thetaiotaomicron* | DPP VI is a cytoplasmic enzyme best characterised from Bacillus sphaericus that is expressed during sporulation, and is responsible for the degradation of bacterial cell wall components (Guinand, 2004). LytF (C40.002) and LytE (C40.003) of B. subtilis hydrolyse the bacterial cell wall peptidoglycan, and are cell-separating enzymes (Yamamoto et al., 2003). | MKINFKLFFILVGLTVIFSSCRTSAPRLDYQALARASVLLGIDINLEDDHKLYLEAADWIGAPYRGGGDSKRGTDCSGMVYQIYRKVYRIQVPRNTEELKNKSNKVAKRNLKEGDLVFFSSNRSRKRVAHVGIYLKSGKFIHSSTSRGVIVSRLNEDYYLRHWISGGRIR | Hyp. prot. | Y (Lipo) | Y | 0 | 0 | Unknown | Exp. | 1.3E-106 | 302 | 100 | 82.353 | 16 | Shell |
| **Metabolism** | | | | | | | | | | | | | | | | | | |
| C44 | C44.003 | glutamate synthase (NADPH) large chain | *B. thetaiotaomicron* | glutamate synthase (NADPH) large chain | MKKQELFNNEAKGFPYQRHPKQPGLYDAAYEHDACGVGMLVNIHGEKSHDIVESALKVLENMRHRGAEGADNKTGDGAGIMLQIPHEFILLQGIPVPEKGRYGTGLLFLPKKEKDQATILSIIIEEIEKEGLTLMHLRNVPTCPEILGEAALANEPDIKQVFITGFTETETADRKLYLIRKRIENKVRMSAIPAKEDFYIVSLSTKSIIYKGMLSSLQLRNYYPDLTNNYFTSGLALVHSRFSTNTFPTWGLAQPFRLLAHNGEINTIRGNRGWMEARESVLSSPTLGDIKEIRPIIQPGMSDSASLDNVLEFLVMSGLSLPHAMAMLVPESFNEKNPISEDLKSFYEYHSILMEPWDGPAALLFSDGRFAGGMLDRNGLRPARYLITKNDMMVVASEVGVMDFEPGDIKEKGRLQPGKILLVDTEKGEIFYDGELKKQLAEAKPYRIWLSTNRIELDELKSGRKMPHHVENYDRMLRTFGYSKEDIEKLIIPMASTGAEPIHSMGNDTPLAVLSDKPQLLYNYFRQQFAQVTNPPIDPLREELVMSLTEYIGAVGMNILTPSESHCKMVRLNHPILSNTQLDILCNIRYKGFKTVKLSMLFEVAKGKAGLQEALTELCKQAEASVTEGVNYIVLTDRNVDATHAVIPSLLAVSAVHHHLISVGKRVQTALVVESGEIREVMHAALLLGFGASALNPYMAFAILDKLVKNKDIQLDYATAEKNYIKSICKGLFKIMSKMGISTIRSYRGAKIFEAIGLSEELSKAYFGGLGSPIGGIRLEEVARDAIAFHNEGFAAEAGGLLPNKGLYSFRKDGEKHAWNPETISTLQLATRLGSYKKFKEYTHLVDEKEKPIFLRDFLNFRRNPISIEQVEPVESILRRFVTGAMSFGSISKEAHEAIAIAMNRIHGRSNTGEGGEDAARFQPLPDGNSMRSAIKQVASGRFGVTTEYLVNADEIQIKIAQGAKPGEGGQLPGFKVNDVIAKTRHSIPGISLISPPPHHDIYSIEDLAQLIFDLKNINPQAKISVKLVAESGVGTIAAGVAKAKADLIVISGAEGGTGASPASSIRYAGISPELGLSETQQTLVLNGLRGQVVLQADGQLKTGRDIILMALMGAEEYGFATSALIVLGCVMMRKCHQNTCPVGVATQNEELRKRFHGRSEYLVNFFTFLAQEVREYLAEMGFTKMDDIIGRTDLIERKSGTDDPNPKHALIDFTRLLTRIDNSAAIRHVIDQDHAISTVKDVTIIDAAQAAIEHEKEISLEYTIANTDRAIGAMLSGVIAKKYGERGLPEHTLNVKFKGSAGQSFGAFLVPGVNFKLEGEANDYLGKGLSGGRIAVLPPIRSNFEAEKNTIAGNTLLYGATSGEVYINGRVGERFAVRNSGAVAVVEGVGDHCCEYMTGGRVVVLGQTGRNFAAGMSGGVAYVWNKEGNFDYFCNMEMVELSLIEEASYRKELHELIRQHYLYTGSKLARTMLDDWNHYVDQFIQVVPIEYKKVLQEEQMRKLQQKIADMQRDY | glutamate synthase | N | N | 0 | 0 | Cyto. Membr. | Ic | 0 | 2950 | 100 | 93.359 | 47 | Core |
| C26 | C26.957 | GMP synthase | *B. thetaiotaomicron* | GMP synthase | MQEKIIILDFGSQTTQLIGRRVRELDTYCEIVPYNKFPKEDTTIKGVILSGSPFSVYDKDAFKVDLSEIRGKYPILGICYGAQFMSYTNGGKVEPAGTREYGRAHLTSFCKDNVLFKGVRENTQVWMSHGDTITAIPANFKKIASTDKVDIAAYQIEGENVWGVQFHPEVFHSEDGTQILKNFVVGVCGCKQDWSPASFIESTVAELKAQLGDDKVVLGLSGGVDSSVAAVLLNRAIGKNLTCIFVDHGMLRKNEFKNVMNDYECLGLNVIGVDASEQFFAELAGVTEPEGKRKIIGKGFIDVFDVEAHKIKDVKWLAQGTIYPDCIESLSITGTVIKSHHNVGGLPEKMNLKLCEPLRLLFKDEVRRVGRELGMPEHLITRHPFPGPGLAVRILGDITPEKVRILQDADDIFIQGLRDWGLYDQVWQAGVILLPVQSVGVMGDERTYERAVALRAVTSTDAMTADWAHLPYEFLGKISNDIINKVKGVNRVTYDISSKPPATIEWE | GMP synthetase | N | N | 0 | 0 | Cyto. | Ic | 0 | 1020 | 100 | 97.436 | 47 | Core |
| C26 | C26.957 | GMP synthase | *Lactobacillus sakei* |  | MKQDMIVILDLGSHENTVLARAIRALGVYSEIYPHDITVEELKALPNVKGIIINGGPNNVIDGVAIDVNPAIYAMGIPVMAAGHDKAACEVKLAEFTDDIEAIKNAVKAFVFDTCKAEANWNMTNFVNDQIELIKRQVGDKKVLLALSGGVDSSVVAALLLKAIGDNLVCVHVNHGLMRKGESEDVVEVFSNQLKANLIYLDVTDRFLDKLAGVEDPEQKRKIIGSEFIRVFEEEARKLDGIDFLGQGTIYPDIVESGTKTAKMVKSHHNVGGLPEDLKFQLVEPLRQLFKDEVRACGLELGLPYEMVYRQPFPGPGLGVRCLGAITRDRLEAVRESDAILREEFQIAGLDKKVWQYFTVVPDFKSVGVRDNARSFDWPVIIRAVNTVDAMTATIEPIDWPVLMKITDRILKEVKNVNRVCYDMSPKPNATIEWE | GMP synthase | N | N | 0 | 0 | Cyto. | Ic | 8.46E-152 | 441 | 80 | 59.942 | 47 | Core |
| C26 | C26.965 | imidazole glycerol phosphate synthase subunit HisH | *B. thetaiotaomicron* | imidazole glycerol phosphate synthase subunit HisH | MKVAVVKYNAGNIRSVDYALKRLGVEAVITADKEELQSADKVIFPGVGEAETTMNHLKATGLDELIKNLRQPVLGICLGMQLMCRYSEEGGVDCLNIFDVDVKRFVPQRHEDKVPHMGWNTIGKTNSKLFEGFTEEEFVYFVHSFYVPTCNFTAATTDYIHPFSAALHKDNFYATQFHPEKSGKTGEKILTNFLNL | imidazole glycerol phosphate synthase, glutamine amidotransferase subunit | N | N | 0 | 0 | Cyto. | Ic | 1.13E-138 | 385 | 100 | 93.367 | 47 | Core |
| S16 | S16.A04 | DNA repair protein RadA | *B. thetaiotaomicron* | DNA repair protein RadA | MAKEKTVYVCSNCGQDSPKWVGKCPSCGEWNTYVEEIVRKEPVNRRPVSGIETQKPKPVILSEIIADDEPRINMHDNELNRVLGGGLVPGSLVLIGGEPGIGKSTLVMQTVLRMPEKKILYVSGEESARQLKLRADRLSEVSSDCFIVCETSLEQIYVHIKNTNPDLVIIDSIQTISTETIESSPGSIAQVRECSASILRFAKETHTPVLLIGHINKEGSIAGPKVLEHIVDTVLQFEGDQHYMYRILRSIKNRFGSTAELGIYEMRQDGLRQVSNPSELLLSQDHEGMSGVAIASAIEGVRPFLIETQALVSSAVYGNPQRSATGFDIRRMNMLLAVLEKRVGFKLAQKDVFLNIAGGLKVNDPAIDLAVISAILSSNMDAAIEPEVCMAGEIGLSGEIRPVNRIEQRIGEAEKLGFKRFLLPKYNLQGIDTKKLKIELVPVRKVEEAFRALFG | DNA repair protein RadA | N | N | 0 | 0 | Cyto. | Ic | 0 | 905 | 100 | 97.363 | 47 | Core |
| C14B | C14.UPB | UP | *Koribacter versatilis* | The apoptosis cascade, known mainly in animal cells, is primarily controlled by the caspases. It is thought that the caspases with long prodomains are responsible for the initiation of the apoptotic response whereas those with shorter prodomains are "effector" caspases (Earnshaw et al., 1999). The effector caspases are activated by the initiator caspases and are directly responsible for cell death (Thornberry & Lazebnik, 1998). Caspase-1, the type example of family C14, is involved in the processing of interleukin 1 beta precursor and does not have a role in apoptosis. | MANFESSVKVIPYSQERVYNKLSDLSNLEAIKDRLPQDKVEDLSFDSDTLSFTVSPVGQLTLQIVERDPCKSIKLATTNSPLPFNMWIQLVETAEEECNVKVTIEIDINPFMKAMVQKPLQEGLEKMVEMLAVINY | polyketide cyclase | N | N | 0 | 0 | Unknown | Ic | 0.0000000000000000201 | 80,9 | * | 31.1 | 4 | Cloud |
| M64 | M64.UPW | UP | *B. thetaiotaomicron* | The activity of the enzyme in degrading IgA may help the organism to exist as a commensal organism in the human intestine. | MKQFCCTLLFFLATLTSYAQNFADFFQDKTLRVDYIFTGDANRQAIYLDELSQLPSWAGRQHHLSELPLAGNGQIIVKDVATGQCIYKTSFSSLFQEWLSTDEARTTAKGFENTFLLPFPKKTVEVEITLYSPRKDVMANFKHIVSPEDILIHKRGTSHVTPHRYMLQSGSEKDCIDVAILAEGYTEKEMDVFYQDAQRTCESLFSHEPFRSMKQKFNIVAVASPSTDSGVSVPRADQWKQTAVHSHFDTFYSERYLTTSRVKSIHNALAGIPYEHIIILANTDVYGGGGIYNSYTLTTAHHPMFKPVVVHEFGHSFGGLADEYFYEDDTMTDTYPLDVEPWEQNITTRVNFASKWEDMLPPHIPVPTPVAQQKNYPVGVYEGGGYSFKGIYRPAYNCRMKTNEHPEFCPVCQRAIRRIIEFYVP | Hyp. prot. | Y (SP) | Y | 0 | 0 | Mul. loc. | Exp. | 0 | 794 | 100 | 86.824 | 13 | Shell |
| S01C | S01.453 | MucD peptidase | *B. thetaiotaomicron* | MucD peptidase (heat shock protein, stress reponse?) | MKQTTKNILGVGAIVLLSSGVAGLTTYKLLQSNEAATETSFNEMFKQNPNVKLAAFDAVNAQPVDLTQAAENSLHAVVHIRSTQEAKTRTVQQAPDIFDFFFGDGRGQQRQVQTQPRVGFGSGVIISKDGYIVTNNHVIDGADEISVKLNDNREFKGRVIGTDPSTDLALVKIEGDDFPTIPVGDSEALKVGEWVLAVGNPFNLNSTVTAGIVSAKARSLGVYNNGIESFIQTDAAINQGNSGGALVNAKGELVGINSVLSSPTGAYAGYGFAIPTSIMTKVIADLKQYGTVQRALLGIRGGSIGSSLMDDRQPIDNSGKTLADKAKELGVVEGVWVSEIVENGSASGADIKVDDVIIGLDNKKVSNMADLQEAIAKHRPGDKVKVKLIRDKKEKTVEVTLKNEQGTTKIVKDAGMEILGAAFKELPDDLKKQLNLGYGLQVTGVSSGKMSDAGVRKGFIILKANDQPMRKVSDLEEVMKAAVKSPNQVLFLTGVFPSGKRGYFAVDLTQE | type I deoxyribonuclease HsdR | N | Y | 0 | 0 | Per. | Exp. | 0 | 996 | 100 | 96.477 | 47 | Core |
| **Nutrition** | | | | | | | | | | | | | | | | | | |
| C11 | C11.006 | *NaN* | *B. thetaiotaomicron* | Clostripain may well contribute to the nutrition of Clostridium sp. by breaking down proteins extracellularly. | MKTKQKYYHLIKPIIFVLFLSFSMASCEKEDIIPESPDIEDTENPTDTDPNEIVPPIVRANNEQTIFMYLPWSNNLTSNFYQNISDLESVIEKNILKNERVIVFMCTEATEATLFELVYENGKSVRKTYKQYTDPVYTTAEGITSILNDVRECTPAQRYSMIIGCHGMGWIPVSNAQSRSNLCVNKMHWEYENVPMTRYFGGLYPQYQTDITTLAQGISNAGLKMEYILFDDCYMSTVEVAYDLKNVTNHLIASTCEIMAYGMPYAKIGQYLIGEINYEKICNGFDDFYSNYEMPCGTIAVTDCAELDHLATIMKDINSQYTFDTTLTGSLQRLDGYSPVIFFDCGDYVSKLCSNQELLAQFNEQLNRTVPFKRNTDYFYSMSRGKVKIETFSGITISDPSTNDLASAKEKTAWYAATH | Hyp. prot. | Y (Lipo) | Y | 0 | 0 | Mul. loc. | Exp. | 0 | 642 | 100 | 73.747 | 7 | Cloud |
| M13 | M13.UPW | UP | *B. fragilis* | The enzymes act outside animal cells to degrade or convert polypeptide substrates. In bacteria they are presumed to have a nutritional role. | MKVTKYLPILAVCLMTTGCNSKKEAVLTSGIDLANLDTMAMPGTSFYQYACGGWVAAHPLTDEYSRFGTFDMLRENSREQLKTLIAELAAKKDNAPGSAAQKVGDLYNIAMDSVKLNQEGVAPIKAELEAIDALQDKGEIYTYIAEIQKKGINPYFSLYIGADDMNSSMNLVQTYQGGIGMGQRDYYLENDEQTKNIRDKYQEHIAKMFQLAGYDEAAAQKAVKAVMNIETRLAKSARSQVELRDPHANYNKMDMETLKKNFPTFAWDVYFTTSGLNDLKEVNVGQPAAMKEVADVINTVSLDEQKLYLQWNLINSAASYLSDDFVAQDFDFYGKTMSGKKEMQPRWKRAVSTVDGALGEVVGQMYVEKYFPAAAKERMVTLVKNLQTSLGERINALEWMSEPTKAKAQEKLATFHVKIGYPDTWKDYSALDIKNDSYWANIERANEWGYAEMISKAGKPVDKDEWLMTPQTVNAYYNPTTNEICFPAAILQPPFFDMNADDAMNYGAIGVVIGHEMTHGFDDQGRQYDKDGNLKDWWTEEDAKKFEERAQVMVNFFDSIEVAPGVHANGSLTLGENIADHGGLQVSYHAFKKAMETAPLEVVDGFTPEQRFFLAYANVWAGNIRPEEILRLTKLDPHSLGKWRVDGALPHIANWYEAFNITEQDSMFVPKEKRVSIW | peptidase M13 | Y (Lipo) | Y | 0 | 0 | Cyto. | Exp. | 0 | 1157 | 100 | 80.236 | 47 | Core |
| S08A | S08.UPA | UPs | *Chitinophaga pinensis* | In bacteria, archaea and fungi, family members are probably involved in nutrition. Most are secreted, but lactocepin (S08.018) is attached to the outer surface of the cell wall, and lantibiotic leader peptidases are intracellular. Lantibiotic leader peptidases, such as the CylP leader peptidase (S08.086), are required for the activation of lantibiotics and cytolysins. Subfamily S8A peptidases from Dichelobacter have been implicated in the pathogenesis of ovine foot-rot (Kortt & Stewart, 2004) and C5a peptidase (S08.020) from pathogenic streptococci destroys the complement chemotaxin C5a (Cleary & Matsuka, 2004). Site-1 protease (S08.063) is a mammalian member of subfamily S8A which catalyzes the first step in the proteolytic activation of sterol regulatory element-binding protein (Sakai et al., 1998). TPP-II (S08.090) is a mammalian cytoplasmic enzyme that degrades peptides and has a role in the processing of antigenic peptides and the degradation of cholecystokinin (Warburton & Bernardini, 2002). In subfamily S8B, kexin (S08.070) processes the yeast alpha-mating factor and killer toxin precursors, and furin (S08.071) is located in the mammalian trans-Golgi network and endosome membranes where is processes a variety of proproteins. | MKKLISLILCCLICGITSAQLIKQKVEKQKKQSELDWYNCSFDRDSVYGAEVNKAYEYLNANKKKLKKRPIVALIGTGMDVEHEDLRQAIWINPKEKLNQKDDDRNGLIDDINGWNFLGGKDAQVVESLTREGEREFFRLKDKYADYIFDGKKYYKIINGTRQEVAAPENMEEYNYYRYKVMPESRIGSTYSGLQLAYVIEEYVEKFNRDMKQRFPRKELTVEEFQSCYDPKAERDSLSEVAFVCTAYYFSLYNTDKWEPVYQNMGKKSVETAKASYEEALRKYGTDQRKEITGDNPMDINDNNYGNNILLTSDAATNIMKAGIIAAKRDNKIGSDGIADQAEIMTLRICTGEGEPYLKDMALAIHYAVSHGADVIVLPEQNMLYPEEQKQWIIHELKEAEKKGAIVIVPAWNTSIDMDKVEFFPNRKMSKDKELTNLMIVASSDKKGNPVMDTNYGANTLDIYAPGTDIYSAYMGDTYRTGTGEGLAAATVAGVATLIKSYFPKLTGSQIRDILLKSVTSRKGVEVEKGIRVDDRPSQDLFLFDDLCISGGIVNAYQAILEAEKMNSQKK | Hyp. prot. | Y (SP) | Y | 0 | 0 | Extracel | Exp. | 3.23E-68 | 231 | 98 | 33.107 | 4 | Cloud |
| S08A | S08.UPA | UPs | *B. thetaiotaomicron* |  | MKKLVLLVFALNICLGTFAQFTPGDTLKYRISLKDKAATDYSLQKPEMYLSKKSIERRKRQGLEIDSTDLPVCKKYVDAIRKKGVHVLVTGKWDNFVTVSCNDSMLIAEIAGLPFVRSTERVWRGVAKRASERDSLINKPLRTDSLYGPAITQIKMSHADRLHEAGFKGQGMTIAVIDAGFHNVDKIEAMKNINILGTRDFVNPEADIYAESSHGMSVLSCMAMNQPNVMIGTAPEASYWLLRSEDEYSENLVEQDYWAAAIEFADSVGVDLVNTSLGYYSFDDPTKNYRYRDLNGHYALMSREAAKAADKGIVVVCSAGNSGSGSWKKITPPGDAENVITVGAVNKYGVLAPFSSVGNTADGRVKPDVVAVGLGSDVMGTDGNLRHANGTSFSSPIMCGMVACLWQACPELTAKEIIELVRRSGDRAVFPDNIYGYGIPDLWKAYQSTVNTRR | serine protease | Y (SP) | Y | 0 | 0 | Cyto. | Exp. | 0 | 792 | 98 | 83.893 | 47 | Core |
| M20A | M20.016 | Putative peptidase | *B. thetaiotaomicron* | In general, the peptidases hydrolyse the late products of protein degradation so as to complete the conversion of proteins to free amino acids. The Pseudomonas glutamate carboxypeptidase is a periplasmic enzyme that is synthesised with a signal peptide. Gly-X carboxypeptidase is a yeast enzyme that has a vacuolar localisation, and is synthesised with an N-terminal propeptide. In the cytosolic fractions of mammalian cells there are the non-specific dipeptidase and carnosinase. | MKYDISYMTTEAVSLLKSLISIPSISREETQAADFLQNYIEMAGMQTGRKGNNVWCLSPMFDLKKPTILLNSHIDTVKPVNGWRKDPFTPREENGKLYGLGSNDAGASVVSLLQVFLQLCRTSQKYNLIYLASCEEEVSGKDGIESVLPGLPPVSFAIVGEPTEMQPAIAEKGLMVLDVTATGKAGHAARNEGDNAIYKVLDDIAWFRDYRFEKESPLLGPVKMSVTVINAGTQHNVIPDKCSFVVDIRSNELYSNEELFAEIKKHISCDAKARSYRLNSSQIDEKHPFVQKAVKLGRVPFGSPTLSDQALMSFPSVKIGPGRSSRSHTAEEYIMLKEIEEAVGIYLELLDGLLI | acetylornithine deacetylase | N | N | 0 | 0 | Cyto. | Ic | 0 | 699 | 99 | 94.051 | 47 | Core |
| M20D | M20.UPD | UP | *Enterococcus faecium* |  | MNQLSDRLNSLSPSATLAMSQKSNELKAQGVDVINLSVGEPDFNTPDHIKEAAKKAVDDNFSRYSPVPGYPALRNAIVEKLKKENGLEYTAAQISCANGAKQSVCNAILVLVNPGDEVIVPAPYWVSYPEMVKLAEGTPVIVSAGIEQDFKITPEQLEAAITPKTKALILCSPSNPTGSVYSKEELAGLAAVLAKHPQVIVLADEIYEHINYIGAHQSIAQFPEMKERTVIVNGVSKAYAMTGWRIGFIAGPEWIVKACNKLQGQYTSGPCSVSQKAAEAAYTGTQEPVKEMQKAFERRRDLIVKLAKEVPGFEVNVPQGAFYLFPKCSYFFGKSNGKRKIENSDDLAMYLLEDAHVACVGGTSFGAPECIRMSYATSDENIVEAIRRIKEALAKLK | aspartate aminotransferase | N | N | 0 | 0 | Cyto. | Ic | 2.79E-68 | 229 | 99 | 35.323 | 13 | Shell |
| M20D | M20.UPD | UP | *Enterococcus faecium* |  | MPTISIRGNEMPASPIRKLAPLADAAKQRGVHVFHLNIGQPDLPTPQVAIDAIRNIDRKVLEYSPSAGYRSYREKLVGYYAKFNINLTADDIIITSGGSEAVLFSFLSCLNPGDEIIVPEPAYANYMAFAISAGAKIRTIATTIEEGFSLPKVEKFEELINERTKAILICNPNNPTGYLYTRREMNQIRDLVKKYDLFLFSDEVYREFIYTGSPYISACHLEGIENNVVLIDSVSKRYSECGIRIGALITKNKEIRDAVMKFCQARLSPPLIGQIAAEASLDAPEEYSRETYDEYVERRKCLIDGLNRIPGVYSPIPMGAFYTVAKLPVDDSDKFCAWCLSDFEYEGQTVFMAPASGFYTTPGSGINEVRIAYVLKKEDLTRALFVLQKALEAYPGRTE | aspartate aminotransferase | N | N | 0 | 0 | Cyto. | Ic | 1.61E-33 | 133 | 88 | 30.163 | 47 | Core |
| M20C | M20.012 | Pep581 peptidase | *B. thetaiotaomicron* |  | MSTILSLAPQNVWKHFYSLTQIPRPSGHMEKVTEFLINFGKGLGLESFVDEAGNVIIRKPATPGMENRKGVILQAHMDMVPQKNNDTVHDFEKDPIETYIDGDWVKAKGTTLGADNGLGVAAIMAVLEAKDLKHGPLEALITKDEETGMYGAFGLKPGTVNGEILLNLDSEDEGELYIGCAGGMDVTATLEYKEVAPEEGDIAVKVTLKGLRGGHSGLEINEGRANANKLLVRFIREAVASYEARLASWEGGNMRNAIPREAHAVVTIPAENEEELLGLVKYCEDLFNEEYSTIETPISFTAERVEVPAGQVPEEIQDNLIDAIFACQNGVTRMIPTVPDTVETSSNLAIITIAGGKAEIKILARSSSDSMKEYLTTSLESCFSMAGMKVEMTGGYSGWQPDVNSPILHAMKASYKQQFGVEPAVKVIHAGLECGIIGAIIPGLDMISFGPTLRSPHSPDERALIPTVQKFYDFLVATLEQTPMK | aminoacyl-histidine dipeptidase | N | N | 0 | 0 | Cyto. | Ic | 0 | 963 | 100 | 96.082 | 47 | Core |
| M20C | M20.012 | Pep581 peptidase | *B. thetaiotaomicron* |  | MEKKDLKPAGVFKYFEEICQVPRPSKKEEKMIAYLKAFGAKHNLETKVDEAGNVLIKKPATPGKENLQTVVLQSHIDMVCEKNNDVQHDFLTDPIETEIDGEWLKAKGTTLGADNGIGVATELAILADDSIEHGPLECLFTVDEETGLTGAFALKEGFMSGDILLNLDSEDEGEIFIGCAGGIDSVAEFTYKEVEVPAGYFFFKVEVKGLKGGHSGGDIHLGRGNANKILNRFLTRMATRHDLYLCEINGGNLRNAIPREAYAICAVPEDAKHDVRTELNIFTSEVENELSVTEPDLRLVLESETPRKTAIDQDTTARLLKALYAAPHGVYAMSQDIPGLVETSTNLASVKMKPNNVIRIETSQRSSILSARDDMANTVRSAFQLAGANVTFGEGYPGWKPNPHSAILEVAAESYKRLFGVDAKVKAIHAGLECGLFLDKYPTLDMISFGPTLTGVHSPDERMLIPTVEKFWKHLLDILAHVPAKK | aminoacyl-histidine dipeptidase | N | N | 0 | 0 | Cyto. | Ic | 0 | 966 | 100 | 95.473 | 47 | Core |
| M20B | M20.003 | peptidase T | *B. thetaiotaomicron* |  | MTLVDRFLKYVSFDTQSDESTGITPSTPKQMVFAEYLKTELESLGLEDITLDEHGYLFATLPANIDKDVPTIGFIAHMDTSPDMSGKDVTPRIVEKYDGSDIMLCAEDNIILSPVQFPELLDHKGEDLIVTNGKTLLGADDKAGIAEIVSAVVYLKEHPEIKHGKIRIGFNPDEEIGEGAHKFDVEKFGCEWGYTMDGGEVGELEFENFNAAAAKILFKGRNVHPGYAKNKMINSIYLANQFITLLPSIERPEHTTGYEGFYHLIGIQGEVEQCTVSYIIRDHDRAKFEERKKEIERLVARMNTEYGEGTVTLELRDQYYNMREKIEPVMHIIDIAFAAMEAVGVKPNVKPIRGGTDGAQLSFKGLPCPNIFAGGLNFHGRYEFVPIQNMEKAMNVIVKIAELVASR | peptidase T | N | N | 0 | 0 | Cyto. | Ic | 0 | 780 | 100 | 92.138 | 47 | Core |
| M20F | M20.UPF | UP | *B. thetaiotaomicron* |  | MNEIQKYIAANEPQIMEDLFSLIRIPSISALPEHHDDMLACAERWAQLLLEAGVDEALVMPSQGNPIVFAQKIVDPDAKTVLVYAHYDVMPAEPLELWKSQPFEPEIRDGYIWARGADDDKGQSFIQVKAFEYLLKNGLLRNNVKFIFEGEEEIGSPSLEAFCEEHKELLKADVILVSDTSMLGADLPSLTTGLRGLAYWEIEVTGPNRDLHSGHFGGAVANPINVLCRIISKVTDTDGRITVPGFYDDVEEVPQAEREMIAHIPFDEKKYKEAIGVKELFGEKGYSTLERNSCRPSFDVCGIWGGYTGEGSKTVLPSKAYAKVSCRLVPHQDHHKISQMFTDYILSIAPETVQVKVTPMHGGQGYVCPISLAAYQAAEKGFEIAFGKKPLAVRRGGSIPIISTFEQVLGVKTVLMGFGLESDAIHSPNENFSLDIFRKGIEAVAEFHQEYAGR | Hyp. prot. | N | N | 0 | 0 | Cyto. | Ic | 0 | 903 | 100 | 95.595 | 47 | Core |
| M20B | M20.UNB | Non-peptidase homologues | *Emericella nidulans* |  | MKRINFKTTLMTGLFVLLGMVAKADNNPIVVGNSRFTFITENLVRMEYANHQKFLDDSTLFAVNRQAGNVDVKVEKKDGKYIFSTSMMSVEFENDGFPFGQNNVRVSFEMDGKRKSWCMTDEQRNNLGGAITTLDAIGSPIPLQEGLLSRDGWYLINDTGKDVYKNGWLSVRDRDHVQDLYLFVYGNDYKSALRSLKAISGAVPMTRKYVHGSWYCRWWQYTADDYRDLVKGYHEHDFPLDIMVFDMDWHRKDGKIGTGHAFTRGWTGYSWNRKLIPDPGALIREFHDQQIYVTINEHPHDGIRPHEDVYPEFIRALGIDADKDPVPIFDAGDRKYMDAFMRYAHQESDSMGVAFWWLDWQQDYAYPVVRGTTTKHLPWLNEIYYNYSKQGELRGTGFSRWGGWGDHRHPIQFSGDAVGNWNMLNFEVKLTTTSGNAGCFFWAHDIGGFYDGLDSELYTRWTQFGLLNSSLRIHSVVGDKMDRRPWLWGEREEKAMRRIYHMRSELMPYIYSSVRQCHTDMLPLNRGLYIEYPADKESYKHEEEFLFGDLILASPITQAGSGKDKIVSQSVWFPRGDDWYSLFTGSKYEGGKTSVVSCPLEEFPVFVKGGWPLPMQPYTERMASTPLTTLVIRCYPGSEGSDNTYSLYEDDGLTMQYANGNYATTDMTYKKENGCVTVHVHPVKGNYKGQPQKRAYRIELPGVDVKSKVKVNGKTVKPIVDSSVNGIIISVKATDIRKAVEITVR | alpha-xylosidase | Y (SP) | Y | 0 | 0 | Unknown | Exp. | 8.67E-104 | 345 | 79 | 32.998 | 2 | Cloud |
| M23B | M23.UPB | UP | *B. thetaiotaomicron* | Peptidases of family M23 are used by certain bacteria to lyse cell walls of other bacteria, either as a defensive or feeding mechanism. The soil bacterium Lysobacter enzymogenes is also capable of lysing soil nematodes. The peptidases are synthesized as precursors and are activated extracellularly. Lysostaphin is bound to the cell wall at its C-terminus (Baba & Schneewind, 1996). | MPKKRRSRAFWKNFKFKYKLTVVNENTLEEIVGLRVSKLNGLSVLLSVLAVLFLIAACIIAFTPLQNYLPGYMNSEVRTQIVDNALRVDSLQQILNKQNLYIMNIQDIFSGKVAIDSVQTLDSLTAAREDTLMERTKREEEFRRQYEENEKYNLTTVTSQPDVNGLILYRPTRGMVSDHFNAEKKHFGTDIAANPNESVLATMDGTVILSTYTAETGYLIGVQHNQDLISIYKHCGSLLKKEGERVKGGEAIALVGNSGTLSTGPHLHFELWYKGHPINPEKYIVF | peptidase M23 | N | N | 1 | 1 | Outer Memb. | M | 0 | 559 | 100 | 92.308 | 13 | Shell |
| M23B | M23.UPB | UP | *B. thetaiotaomicron* |  | MNFNCIIKTGLVAVAAMVSLSSFSQDLIARQAPIDKKLKTVDSLALQKQIRAEQSEYPALSLYPNWNNQYVHAYGNAIIPDTYTIDLTGFHMPTPSTKITSPFGPRWRRMHNGLDLKVNIGDTIVAAFDGKVRIVKYERRGYGKYVVIRHDNGLETVYGHLSKQLVEENQLVKAGEVIGLGGNTGRSTGSHLHFETRFLGIAI | Hyp. prot. | Y (Lipo) | Y | 0 | 0 | Outer  Memb. | Exp. | 2.09E-150 | 419 | 100 | 98.522 | 4 | Cloud |
| M23B | M23.UPB | UP | *B. thetaiotaomicron* |  | MKDILILLFVLFFPMIVNAQDKSSFSFREVNHIRVATPGLFAKGNHIYLHLDSLKEHEYAFPLPGGKVISAYGTRGGHSGADIKTCANDTIRVAFDGVVRMSKPYYAYGNLVVIRHANGLETIYSHNCKNLVRSGEVVKAGQPIGLTGRTGRATTEHVHFETRINGQHFNPNLIFDLKERKLRKECIKCSKNGSGVIVKSQAGNNRIAQNKK | peptidase M23 | Y (SP) | Y | 0 | 0 | Outer Membr. | Exp. | 2.26E-128 | 361 | 92 | 88.144 | 2 | Cloud |
| M23B | M23.UPB | UP | *B. thetaiotaomicron* |  | MRKVYYIYNPQTQTYDRIYPTVRQRALSILRRLFYGMGLGAGCFIVLLFIFGSPSEKELRIENSRLQAQYNVLSRRLDDAMGVLQDIQQRDDNLYRVILQADPVSPAIRQAGYGGTNRYEELMDLANAKLVVNTTQKLDVLSKRLYIQSKSFDDVVDMCKNHDEMLKCIPAIQPISNKDLRQTASGYGTRIDPIYGTTKFHSGMDFSAHPGTDVYATGDGTVVKVGWETGYGNTIEIDHGFGYMTRYAHLQGFNTKVGKRVVRGEVIGKVGSTGKSTGPHLHYEVHVKGQVVNPVNYYFMDLSAEDYEKMIQLAANHGKVFD | peptidase M23 | N | N | 1 | 1 | Cyto. | Ic | 0 | 646 | 100 | 95.652 | 1 | Cloud |
| M23B | M23.UPB | UP | *Ochrobactrum anthropi* |  | MKLFDVYPLYNINIVKGKGCKVWDENGTEYLDLYGGHAVISIGHAHPHYVEMISNQVANLGFYSNSVINKLQQQVAERLGKISGYEDYSLFLINSGAEANENALKLASFHNGRTKVVSFSKAFHGRTSLAVEATNNPSIIAPINNNGHVVYLPLNDVEAMKQELSRGDICAVIIEGIQGVGGIKIPTTEFMQELRKACTETGTILILDEIQSGYGRSGKFFAHQYADIKPDIITVAKGIGNGFPMAGVLISPMFKPVYGQLGTTFGGNHLACSAALAVMDVIEQENLVENAAKVGNYLLEELKKFPQIKEVRGRGLMIGLEFDEPIKELRSRLIYDEHVFTGASGTNVLRLLPPLCLSMEEADEFLARFKKVL | acetylornithine aminotransferase | N | N | 0 | 0 | Cyto. | Ic | 6.99E-43 | 160 | 95 | 30.769 | 47 | Core |
| S15 | S15.UPW | UP | *Saccharomonospora viridis* | Zevaco et al. (1990) and Lloyd & Pritchard (1991) have demonstrated that X-Pro dipeptidyl-peptidases are able to hydrolyze sequentially peptides derived from beta-casein. Therefore, it is believed that their role is in the degradation of caseins. It has been suggested that X-Pro dipeptidyl-peptidases have contrasting roles in the proteolytic systems of lactobacilli and lactococci (Chich, 2004). In lactobacilli X-Pro dipeptidyl-peptidase is involved in the casein-degradation pathway, providing essential amino acids to the lactobacilli (Y�ksel & Steele, 1996). | MGEQVIVLTDLTKQYGNFTAVDHIRLHIQKGEIFGLLGPNGAGKSTTILMMLGLTEPTSGSVEICGINSTTHPIEVKRKIGYLPEDVGFYDDMTGLENLIYTARLNGIPDKEAREKAMELMKRTGLEDQLKKKAGKYSRGMRQRLGLADVLIKNPEIIILDEPTSGIDPAGVQEFIELIRWLSKEKGLTVLFSSHHLDQVQKVCDRVGLFSNGKILALIDMTELKDKKQELSDIYNHYFEEGGEQHE | multidrug ABC transporter ATP-binding protein | N | N | 0 | 0 | Cyto. | Ic | 2.62E-41 | 151 | 89 | 37.557 | 47 | Core |
| S15 | S15.UPW | UP | *Saccharomonospora viridis* |  | MSITIKNLNKIYPNGNHALKDVNLEIPTGMFGLLGPNGAGKSTLMRILVALMEPTSGQVEICGYDLMKQRKEIRGILGYLPQDFRFFAKYKTYEFLDYAARLSGMTQNRQRKQAVDEMLENVGLFDVRERYANKLSGGMKRRLGIAQALIHHPKVIIVDEPTTGLDPEERIRFRNLLSEVSENDVTIILSTHIVGDISSTCNNMALMNRGEVSFYGSPQDMLKKAEGKVWRIRAGGDQLHEIDKKYPVISTIPSGTAWEVQVVADKVEGYEAEPFPPNLEHAYVYYMENQLNLWTND | multidrug ABC transporter ATP-binding protein | N | N | 0 | 0 | Cyto. | Ic | 1.55E-34 | 133 | 77 | 33.19 | 47 | Core |
| M03A | M03.005 | peptidyl-dipeptidase Dcp | *B. thetaiotaomicron* | Most of the peptidases are synthesised without signal peptides or propeptides, and function intracellularly. Mitochondrial intermediate peptidase is an exception, having a typical mitochondrial leader peptide. One likely function of peptidases in family M3 is the intracellular degradation of oligopeptides. These could include cleaved signal peptides, and products of protein degradation. In vertebrate organisms, some of these peptides might otherwise be bound by MHC class I (York et al., 2003). Many mammalian, biologically-active peptides are excellent substrates for the oligopeptidase actions of thimet oligopeptidase and neurolysin, and some authors have proposed important roles for the enzymes in the catabolism of such peptides. In support of this, evidence has been offered that the predominantly intracellular enzymes are to some extent membrane-bound or secreted. | MMIKKTLTILAASCMMYSCTTKTESNPFFTEFQTEYGVPSFDKIKLEHYEPAFLKGIEEQNQNIEAIIESPEVPTFENTIVALDNSAPILDRVSAIFFNMTDAETTDSLTELSIKLAPVLSEHEDNISLNQTLFKRINDVYQQKDSLNLTIEQQRLLDKTYKSFVRSGANLDTKQQARLREINKELSTLGITFSNNVLNENNAFQLFVDKQEDLAGLPEWFCQSAAEEAKAAGQPGKWLFTLHNASRLPFLQYSENRPLREKMYKAYINRGNNNDKNDNKEVISKIISLRLEKANLLGFDCYANFVLDETMAKNANNVMDLLNNLWSYALPKAKSEAAELQQLMDKEGKGEKLEAWDWWYYTEKLRKEKYNLSEEDTKPYFKLENVREGAFAVANKLYGITLSKLEDIPTYHPDVEVFEVKDVDGSQLGIFYVDYFPRPGKSGGAWMSNYREQSGPIRPLVCNVCSFTKPIGDTPSLLTMDEVETLFHEFGHALHGLLTQCKYKGTSGTNVVRDFVELPSQINEHWATEPEVLKMYAKHYATGKVIPDEIIEKILQQKTFNQGFMTTELLAAAILDMNLHMLKDVKNLDVVSYEKEAMDKLNLISEIAPRYRVTYFNHIIGGYAAGYYSYLWANVLDNDAFEAFKEHGIFDKNTAELFRRNVLEKGDSEDPMTLYKNFRGTEPSMEPLLKNRGMK | peptidase M3 | Y (Lipo) | Y | 0 | 0 | Cyto. | Exp. | 0 | 1317 | 99 | 91.499 | 47 | Core |
| M03A | M03.005 | peptidyl-dipeptidase Dcp | *B. thetaiotaomicron* |  | MNNITNAQNPFFGQYQTPHATVPFDRIKTEHYEPAILEGIKQQNAEIDAIILNPEKANFNNTIEAFEESGELLDRVVSVFGNMLSAETNDDLQELAQKIMPLLSEHSNNITLNEKLFARVKEVYDQKETLQLTQEQSQLLENAYNSFVRHGANLEGEAREEYRKLTTELSKLTLDFSENNLKETNSYQMLLTKKESLAGLPEIIVEAAAETAKSEGKEGWAFTLHAPSYVPFMTYADNRDLRHRLYMAYNTKCTHDNEFNNIDIVKKIANTRMKIAQLLGYKDYAEYTLKRRMAENSRAVYKLLNQLLEAYTPTAEAEYKDVQELARLEQGNDFIIMPWDWSYYSNKLKDKKFNINEEMLRPYFELEQVKKGVFGLAEKLYGITFRKNTEIPVYHKEVEAYEVFDKDGQFLAILYTDFHPRLGKRAGAWMTSYKEQWIDKKTGENSRPHISVVMNFTKPTENKPALLTFNEVETFLHEFGHSLHGMFANSTYKSLSGTNVYWDFVELPSQIMENFAIEKDFLNTFARHYQTGEVLPDELIQRLVDASNFNVAYACLRQISFGLLDMAWYTRNVPFEGDVKVYEQEAWKKAQILPVVKETCMSTQFSHIFAGGYSAGYYSYKWAEVLDADAFSLFKQKGIFNQDVAESFRNNILSKGGTEHPMVLYKRFRGQEPTIDALLIRNGIKN | peptidase M3 | N | N | 0 | 0 | Cyto. | Ic | 0 | 1304 | 99 | 89.489 | 47 | Core |
| S46 | S46.002 | dipeptidyl-peptidase 11 | *B. thetaiotaomicron* | DPP-7 probably has a nutrional role, perhaps required for the further digestion of peptides generated by proteinases such as the gingipains (see family C25). | MRKQILFAIFSLATLIIHADEGMWMLPDLKTQNEIAMRELGLEIPIEEVYNANGLSLKDAVVHFGGGCTGEVISSEGLVLTNHHCGYGAIQQHSNVEHDYLTEGFWAMNRDAELPTPGLKVTFIDRILDVTDYVNEQLKKDKDPEGTNYLSPTYLNKVAERFAKAENIEITPTTKLELKAFYGGNKYYLFVKTVYSDIRMVGAPPSSIGKFGADTDNWMWPRHTGDFSLFRIYADKNGKPAEYSKDNVPLHVKKHLTISLAGVQEGDFTFVMGFPGRNWRYMISDEVEERMQTTNFMRQHVRGARQKVLMEQMLKDPAVRIHYASKYASSANYWKNAIGMNEGLVRLKVLDTKRAQQEELLARGREKGDNSYQKAFDEIRSIVAHRRDALYHQQAINEALVTALDFMRIPSTTALATALKSKDKEKIKEAAQNLKQEGEKYFASVPFPEVERMVAKEMLKTYANYIPAEQRINIFEIIDSRFKGNIDAFVDACFDNSIFGNQKNFEKFIKKPSLYKIGYDWMILFKYSITDGILKTAIAMKEANENYDAAYKVWVKGMMNMRQEKGIPIYPDANSTLRLTYGQVLPYEPADGVVYDAHTTLKGVMEKEDPGNWEFVVPQKLKELYNSKDYGQYGKNGEMPVCFIVNTDNTGGNSGSPVFNSKGQLIGTAFDRNFEGLTGDIAFRPSSQRAACVDIRYTLFIIDKFAGASHIIDELTICEQ | Hyp. prot. | Y (SP) | Y | 0 | 0 | Cyto. | Exp. | 0 | 1331 | 95 | 91.63 | 47 | Core |
| S46 | S46.002 | dipeptidyl-peptidase 11 | *B. thetaiotaomicron* |  | MNRLRLYLLALTALIVCSAKADEGMWLLQLMQQQHSIDMMKKQGLKLEAQDLYNPNGVSLKDAVGIFGGGCTGEIISPEGLILTNHHCGYASIQQHSSVEHDYLTDGFWATSRDKELPTPGLKFTFIERIEDITDIVNAKIAAKEITESESFSSAFLQKLANELYNKSDLKDKKGIVPQALPFYAGNKFYLFYKKIYPDVRMVAAPPSSIGKFGGETDNWMWPRHTGDFSMFRIYADANGEPAEYSANNTPLKTKKHLSISLKGLKEGDYAMIMGFPGSTSRYLTVSEVKERMESENDPRIRIRGARLAVLKEVMNASDKIRIQYANKYAGSSNYWKNSIGMNRAIIDNDVLGTKAAQEAKFAEFAKEKNNADYATVVKKIDDLVAKTAPLNYQFTCLRETFFGAIEFGSVMLAKTREALIEKNDSLIKVRIEALKDTYESIHNKDYDHEVDRKVAKVLFPLYAEMIPADQRLSIYKVIEQKYKGNYDKFVDDMYDNSIFSNRENFEKFVKKPSVKAIDNDLALQYCQSKYDQFEKIVSQLEDMDKELTLLHKTYIRGLGEMKQPVPSYPDANFTIRLTYGNVKPYDPKDGVHYNYYTTTKGILEKENPEDREFVVPAKLKELIEKKDYGRYALPNGDMPVCFLSTNDITGGNSGSPVLNENGELIGCAFDGNWESLSGDINFDNNLQRCINLDIRYVLFILEKLGNCGHLINEMTIVE | Hyp. prot. | Y (SP) | Y | 0 | 0 | Mul. loc. | Exp. | 0 | 1332 | 97 | 90.647 | 47 | Core |
| S46 | S46.001 | dipeptidyl-peptidase 7 | *B. thetaiotaomicron* |  | MKFRLTAVIVLALCLSNAFADEGMWLLGNLKKNKQTDRVMRELGLQMPVNKLYNPKKPCLADAVVSFGGFCSGVVVSEDGLVFTNHHCGFSSIQQHSSVEHDYLKDGFVARSLEEELPNPELYVRFLLRTENVTKRVLSAAKHAKTESERRVVVDSVMNVIGMEVSEKDSTLTGIVDAYYAGNEFWLSVYRDYNDVRLVFAPPSSVGKFGWDTDNWMWPRHTGDFSVFRIYANTQNGPADYSSDNVPYHPEYVAPVSLEGYKEGSFCMTLGYPGSTERYLSSYGIEEMMNGINQAMIDVRGVKQAIWKREMDRRPDIRIKYASKYDESSNYWKNSIGTNKAIQHLKVLEKKRAAEAALREWIQAHPEEREKLIRLFSSLELNYGNRREINRALAYFGEAFINGPELVQLALEILNFDFEAEEKQVVSRMKKLLEKYDNLDTAIDKEVFAAMLKEYQTKVDKKYLPAMYDKIDTLYNGNIQAYVDSLYATSNITSPKGLKRFLERDTTYNLIEDPAVSLSLDLIVKYYEMNQSISEASEQIEQGERLFNDAMRRMYADRNFYPDANSTMRLSFGTVSGYSPFDGATYGYYTTVKGIFEKVKEHAGDIDFAVQPELLSLLSSRDFGRYANEQGDMNVCFISNNDITGGNSGSAMFNGKGELLGLAFDGNWEAMSSDIVFEPDLQRCIGVDVRYMLFIIEKYGKAGNLIKELKIR | Hyp. prot. | Y (SP) | Y | 0 | 0 | Mul. loc. | Exp. | 0 | 1375 | 99 | 91.842 | 47 | Core |
| S33 | S33.UNW | Non-peptidase homologues | Saccharomonospora viridis | Most members of the family are synthesized with signal peptides and are thus either secreted or periplasmic enzymes. Prolyl aminopeptidase activity is not essential for bacterial growth, but may confer a selective advantage allowing a bacterium to utilize proline-rich substrates. | MGLLDGKTAIVTGAARGIGKAIALKFAAEGANIAFTDLVIDENAENTTKELEAMGVKAKGYASNAANFEDTAKVVEEIHKDFGRIDILVNNAGITRDGLMMRMSEQQWDMVINVNLKSAFNFIHACTPVMMRQKAGSIINMASVVGVHGNAGQANYAASKAGMIALAKSIAQELGSRGIRANAIAPGFILTDMTAALSDEVRAEWAKKIPLRRGGTPEDVANIATFLASDMSSYVSGQVIQVDGGMNM | beta-ketoacyl-ACP reductase | N | N | 0 | 0 | Cyto. Cyto. | Ic | 2.67E-26 | 107 | 77 | 32.487 | 47 | Core |
| S33 | S33.UPW | UP | *B. thetaiotaomicron* |  | MNTLKTVYIATIITGLFTGLFLSSSASAQDISGTWHGKLTVPTGSLTIVFHINQAAQGTYVTTLDSPDQGANGIKTQATSFSDSILTIQIPVIYASYKGKLKSDKTITGTFTQGMPIPLNLEKGEASRPKRPQEPQPPFPYKSEEVTVRNEQDGINLAGTLTLPEKGNKFPAVVLVTGSGAQNRDEEIMGHKPFLVIADYLTRNGIAVLRCDDRGTAASQGNHATATNEDFATDTEAAINYLRGRKEINTKKIGIIGHSAGGIIAFIVAAKDPAIAFIVSLAGAGVRGDSLMLKQVEMISKSQGMPDAFWQGTKPSIRNRYAILQQADKTTDELQKELYADVTKTMSSEQLKDLNTVQQISSQISSMTSPWYLHFMRYDPAKAMKKIKCPVLALNGEKDIQVEAAMNLTAIQQRISENGNKNVTVKAYPNLNHLFQTCEKGTLAEYGQLEETISPEVLKDITDWILKHSGKSN | alpha/beta hydrolase | Y (SP) | Y | 0 | 0 | Unknown | Exp. | 0 | 785 | 96 | 80.795 | / | Cloud |
| S33 | S33.UPW | UP | *Emericella nidulans* |  | MKIIAVGMNYARHNKELGHTQINTEPVIFMKPDSAILKDSKPFFIPDFSNEIHYETELVVRINRLGKNIAPRFANRYYDAVTVGIDFTARDLQRKFREQGNPWELCKGFDSSAAIGTFVPVDRYKDIQNLNFNLLIDGKEVQSGCTADMLFKIDDIIAYVSRFVTLKIGDLLFTGTPAGVGPVSIGQHLQGYLEGEKLLDFYIR | 2-hydroxyhepta-2,4-diene-1,7-dioate isomerase | N | N | 0 | 0 | Cyto. | Ic | 9.34E-19 | 84,3 | 88 | 30.159 | 47 | Core |
| S33 | S33.UPW | UP | *Salinibacter ruber* |  | MKISHIEHLGIAVKSIEEALPYYENVLGLKCYNIETVEDQKVRTAFLKVGETKIELLEPTCPESTIAKFIENKGAGVHHVAFAVEDGVANALAEAESKEIRLIDKAPRKGAEGLNIAFLHPKSTLGVLTELCEH | methylmalonyl-CoA epimerase | N | N | 0 | 0 | Cyto. | Ic | 6.86E-29 | 109 | 98 | 39.695 | 47 | Core |
| S33 | S33.UPW | UP | *Trichodesmium erythraeum* |  | MKAFVFPGQGAQFVGMGKDLYENSALAKELFEKANDILGYRITDIMFNGTDEDLRQTKVTQPAVFLHSVISALCMGDDFKPEMTAGHSLGEFSALVAAGALSFEDGLKLVYARAMAMQKACEATPSTMAAIIALPDEKVEEICTTVNAEGEVCVPANYNCPGQIVISGSVPGIEKACELMKAAGAKRALPLKVGGAFHSPLMDPAKVELEAAINATEIHTPKCPVYQNVDALPHTDPVEIKKNLVAQLTASVRWTQSVKNMIADGATDFTECGPGAVLQGLIKKIDGTVSAHGIV | malonyl CoA-acyl carrier protein transacylase | N | N | 0 | 0 | Cyto. | Ic | 1.41E-36 | 139 | 96 | 31.229 | 47 | Core |
| S33 | S33.990 | haloalkane dehalogenase | *Polaromonas sp. JS666* |  | MLDDTYFMKQALIEAGKAAERGEVPVGAVVVCKERIIARAHNLTETLNDVTAHAEMQAITAAANVLGGKYLSECTLYVTVEPCVMCAGAIAWAQTGKLVFGAEDEKRGYQRYAADALHPKTVVVKGILADECAALMREFFAAKRK | tRNA-specific adenosine deaminase | N | N | 0 | 0 | Cyto. | Ic | 1.61E-30 | 114 | 99 | 45.946 | 47 | Core |
| C11X | C11.005 | thetapain | *B. thetaiotaomicron* | Clostripain may well contribute to the nutrition of Clostridium sp. by breaking down proteins extracellularly. | MWFASLIFLYLSFVINLKLMKKIKILSLFVCLIALLAACEDEDITPSYGARTVLVYIAGNNSLGQSDFDSKDVSEMIEGMKGTEGTTNNLLVYFAGYKKTAKLIRLIKNGKGEVKQETVMSYDKHNSVSLDVMKDVFRTAFSNYPAKSYGIVFWSHGDGWLHYQNPSTRWWGQDTSDGDYRMNISDLHEALSVAPHFDFMLFDACYMQSVEVIYQLRDRTDYFIGSPTEIPGPGAPYEAVVPALFSQDKPEINIAESYYTVYAEKYNNGIGISNENWTGGVSVSVVKSSELPALATATKGVLQTAASMQQRANIDITGILCYDPLRSKNYHDLMGLMKKIQKNQQAFDNYAHAYQNAVVWKNTTDNNYCTYPSGYGEMVSMNGFEGLSTYILRGNDVKQDAYYRQSVEWYSAAGWADVYW | peptidase C11 | Y (Lipo) | Y | 0 | 0 | Unknown | Exp. | 0 | 588 | 96 | 70.72 | 26 | Shell |
| **Protein Activation** | | | | | | | | | | | | | | | | | | |
| A31 | A31.UPW | UPs | *Dechloromonas aromatica* | The functions of HycI and HybD are in the processing of the precursors of bacterial hydrogenases to their active forms. It is believed that the precursor has an open conformation facilitating coordination of the metal ligand, and that once the metal ion is in place, cleavage of the precursor causes a conformational change that locks the metal in place. | MESEQTSVLRPLILVAEDDDSNFKLIKAIIGKKCDIEWAKNGEEMVQLFQEHQHRASAILMDIKMPLMNGLEATKIIRESNTEIPIIMQTAYAFSSDKENAMKAGATEVLVKPITLSILRTTLSKYLPDLQW | two-component system response regulator | N | N | 0 | 0 | Cyto. | Ic | 0.0000000000491 | 59,7 | 87 | 32.203 | 47 | Core |
| M24A | M24.001 | methionyl aminopeptidase 1 | *B. thetaiotaomicron* | Probable role in cotranslational removal of N-terminal methionine. | MKNFIKGFRFTPSNYPAAVEDKIQKYRKQGYKLPPRKVLRTPEQLEGIRESAKINTALLDHIAENIREGMSTEEIDRLVYDFTTGHGAVPAPLNYEGFPKSVCTSINDVVCHGIPNKNEILKNGDIINVDVSTIYNGYFSDASRMFMIGDVSPEMRKLVQVTKECMEIGIAAAQPWKQLGDVGAAIQEHAEKNGFSVVRDLCGHGVGMKFHEEPDVEHFGRRGTGMMIVPGMTFTIEPMINMGTYEVFVDEADGWTVCTDDGLPSAQWENMILITETGNEILTY | type I methionyl aminopeptidase | N | N | 0 | 0 | Cyto. | Ic | 0 | 565 | 100 | 94.366 | 47 | Core |
| M24A | M24.UPA | UP | *B. thetaiotaomicron* | The methionyl aminopeptidases of subfamily M24A are essential for the removal of the initiating methionine of many proteins, acting co-translationally in association with the ribosomes (Chang et al., 1992). The X-Pro dipeptidase found in eukaryotes has a role in the cleavage of XaaPro linkages found in dipeptides associated with collagen recycling. Deficiency results in an increase of these dipeptides to toxic levels (Myara et al., 1994). | MIFLKTEDEIELLRQSNQLVGRTLAEVAKVVKPGVTTRELDKVAEEFIRDHGATPTFKGFPNQYGEPFPASICTSVNEQVVHGIPGDIVLKEGDIVSVDCGTYMNGFCGDSAYTFCVGEVDEEIRNLLKVTKEALYIGIQNAVQGKRIGDIGYAIQQYCESHSYGVVREFVGHGIGKNMHEDPQVPNYGKRGYGPLMKRGLCIAIEPMITLGDRQVIMERDGWTVRTRDRKCAAHFEHTVAVGAGEADILSSFKFIEEVLGDKAI | type I methionyl aminopeptidase | N | N | 0 | 0 | Cyto. | Ic | 0 | 528 | 100 | 96.981 | 47 | Core |
| M24B | M24.UNB | Non-peptidase homologues | *B. thetaiotaomicron* |  | MLQPELKLRRDKIRSLMALQGIDAALITCNANLIYTYGCVVSGYLYLPLHSPALLFFKRPNNITGEHSFSIRKPEQIVDLLKEKGLPMPAKLMLEGDELPYSEYCRLASLFPETEVVNGTPLIRQARSVKTAIEIEMFRRSGIAHAKAYEQIPSVYRPGMTDIEFSIEIERLMRLQGNLGIFRVFGRSMEIFMGSVLTGDNAGYPSPYDFALGGQGLDPALPGGANKTPLKEGQSVMIDLGGNFNGYMGDMSRVFSIGKLPEEAYAAHQVCLDIQEKIASIAKPGIPCETLYNTAIEMVTAAGFADKFMGTQQQAKFIGHGIGLEINEAPVLAPRMKQELEPGMVFALEPKIVLPGIGPVGIENSWVVTNERIEKLTNCNEEIIELK | peptidase M24 | N | N | 0 | 1 | Cyto. | Ic | 0 | 735 | 100 | 92.248 | 12 | Shell |
| M24B | M24.UPB | UP | *B. thetaiotaomicron* |  | MFAKETYMQRRALLKKNLGSGVLLFLGNDECGLNYEDNTFRYRQDSTFLYYFGLSCAGLSAIIDIDEDKEIIFGDELSIDAIVWMGSQPTLREKCERVGVKDIMPSADIVGYLHKCVQKGKAIHYLPPYRPEHKLKLMDWLGIPPARQEGSVPFIRAVIAQRNYKSAEEIVEIEKACDVTADMHITAMKVLRPGMYEYEVVAEMNRVAQANNCELSFATIATINGQTLHNHYHGNLVKPGDLFLIDAGAEIESGYAGDMSSTVPADKKFTTRQREVYEIQNAMHLESVKALRPGIPYMEVYELSARVMVEGMKALGLMKGNAEDAVREGAHALFYPHGLGHMMGLDVHDMENLGEIWVGYNGQPKSTQFGRKSQRLAIPLEPGFVHTVEPGIYFIPELIDMWKAEKKFADFINYEKVETYKDFGGIRNEEDYLITETGARRLGKKIPLTPDEVEALR | Xaa-Pro aminopeptidase | N | N | 0 | 0 | Cyto. | Ic | 0 | 924 | 100 | 95.842 | 47 | Core |
| M24B | M24.UPB | UP | *B. thetaiotaomicron* |  | MRQSIKERMHALRMTFPPNYIKAFIIPSTDPHLSEYVAPHWMSREWISGFTGSAGTVVVLMNEAGLWTDSRYFLQAAKELEGSGITLYKEMLPETPSITKYLSQKLKPGESVSIDGKMFSVQQVEQMKEELAAYSLQVDLFGDPLKRIWKDRPSIPNSPAFVYDIEYAGKSCEEKVAAIRAELTKKGAYALFLSALDEIAWTLNLRGNDVHCNPVVVSYLLITQDDVIYFISPEKVTKEVNEYLKEQHVKLKNYDEVETYLNTFTGRNILIDPKKTNFAIYSAINPKCNIIRGESPVALLKAIRNEQEIAGIHAAMQRDGVALVKFLKWLEEAVPSGKETELSVDRKLHEFRAAQPLYMGESFDTIAGYKEHGAIVHYSATPESDVPLQPKGFLLLDSGAQYLDGTTDITRTIALGELTEEEKTDYTLILKGHIALAMAKFPVGTRGAQLDVLARMPIWKYGMNFLHGTGHGVGHFLSVHEGPQSIRMNENPVVLQPGMVTSNEPGVYKAGSHGIRTENLTLVCKDKEGMFGDYLKFETITLCPICKKGIVKEMLTNEEIEWLNNYHQIVYEKLSPNLNEEEKVWLQEATASI | peptidase M24 | N | N | 0 | 0 | Cyto. | Ic | 0 | 1102 | 100 | 87.015 | 13 | Shell |
| S26X | S26.UNW | Non-peptidase homologues | *B. thetaiotaomicron* | The use of signal peptides to direct newly synthesised proteins to a secretory pathway is common to all three domains of living organisms, and the peptidases of family S26 are the major enzymes that remove the signal peptides and facilitate secretion. In E. coli the signal peptidase is inserted in the plasma membrane, and the catalytic domain is in the periplasmic space (Dalbey, 2004). In inner-membrane signal peptidases of eukaryotic mitochondria and chloroplasts process proteins that are destined for the inter-membrane spaces of the organelles (Packer & Howe, 2004). | MNIRKFKWILVFAGAIVIVLLLRGFAFTSCFIPSTGMENSIFQGERILVNKWSYGLRIPFMSIFSYHRWRERPVREQDIVVFNNPAGIRQPVIDRREIYIGRCIGVPGDTLFIDSLFSVISPEVQFNPDKKRLYAYPVDKENLITSLMHTLSIDDDGLMGSSDSTHVRSFSRYEYYLLEQAINGNNWIQPLAGKKDTELRPLIVPGKGKFIRVHPWNITLLRNTLVMHEGKQAEIKNDTLYVDGKPTQHCYFTKDYYWVGANNTINLTDSRLFGFVPQDHLIGKASLVWFSKEKDTGVFDGYRWNRFFRTVK | signal peptidase I | N | Y | 1 | 0 | Cyto. Membr. | M | 0 | 558 | 100 | 81.731 | 3 | Cloud |
| S26A | S26.001 | signal peptidase I | *B. fragilis* |  | MKKLLLLLMRLMKGCIWGITILFLGWILLQTVLFASFKVPTDSMVPALLPGDNIIVNKLPMGARLFNLSAAFRQERFVVYRLPALGGIKRNDVLVFNFPYPQHRDSIGFDILKYYVKRCVALPGDSISIHNYHYCVAGYEKDLGNIKSQDSMKERLQQLPENARKRYSFQTYPWNDSIHWNVCNFGPLYIPRMNDVIVMNRKNYLLYRHLIEWEQQKKLQWKGEKAYLDHHRITSYRFRTNYYFMAGDRVFNSQDSRYWGLLPEEYIVGRASFIWKSTDNEGKIRWKRIFKSIT | S26 family signal peptidase | N | Y | 1 | 0 | Cyto. Membr. | M | 1.88E-92 | 276 | 93 | 48 | 29 | Shell |
| S26A | S26.UPA | UP | *B. thetaiotaomicron* |  | MRQATRAQWIKCAIAILLYLVFLIWVRSWWGLIVIPFIFDIYITKKIPWSFWKRSKNPAVRSVMSWVDAIIFALVAVYFVNIYIFQNYQIPSSSLEKSLLVGDFLYVSKMSYGPRVPNTPLSMPLAQHTLPIVGTKSYIEWPQWKYKRVPGFGNVKLNDIVVFNFPAGDTVAVNYQQTTDFYTLAYGEGQRIYSKQIDMDSLTREQQRAVYDLYYAAGRKQILNNPRTYGEVLWRPVDRRENYVKRCVGLPGDTLQIVDGQVMIDGKAIQNPENLQFNYFVQTTGPYIPEEMFRELGISNADRTLMEDSGYEIGLLEMGLDSRNAQGKLNPVYHLPLTKKMYDTLLGNKKLISKIIMEPEAYAGQMYPLNLYTKWDRNNYGPIWIPAKGATITLTPDNLPIYERCIVAYEGNKLEVKSDGIYINGEKTNEYTFKMDYYWMMGDNRHNSADSRYWGFVPEDHVVGKPIVVWLSLDKDRGWFDGKIRWNRLFKWVD | signal peptidase I | N | N | 3 | 2 | Cyto. Membr. | M | 0 | 917 | 100 | 88.462 | 2 | Cloud |
| S49A | S49.UPA | UPs | *B. thetaiotaomicron* | SppA contributes to the destruction of cleaved signal pepides in the periplasmic space of E. coli acting in conjunction with oligopeptidase A, and it probably has a similar function in other bacteria (Ichihara et al., 1984; Novak, P. & Dev, I. K. 1988). | MKDFLKFTLATVTGIILSSIVLFIVSMVTLFGIMSASDTETIVKKNSVMMLDLNGTLVERTQENPLDILSQLFSDDSNIYGLDDILSSIKKAKENENIKGIYLQASSLGTSYASLQEIRNALLDFKESGKFIIAYGDSYTQGLYYLSSVADKVLLNPKGMIEWRGIAAAPVFYKDLLQKIGVEMQIFKVGTYKSAVEPFISTEMSPANREQVTAYINSIWRQVTEGVSNSRYIPVDSLNAYADRMLMFYPAEESVKCGLADTLIYRNDVRNYLKELVEIGKDDQLPMLGLSDMVNVRKNVPKDKSGNIVAVYYASGEITDYAGSSASDEGIVGSKVSRDLRKLKDNDDVKAVVLRVNSPGGSAFASEQIWHAVKELKTKKPVIVSMSDYAASGGYYISCAADTIVAEPTTLTGSIGIFGMVPNVKGLTDKIGLTYDVVKTNKYSDFGNIMRPFNEGEKALLQMTITEGYNTFIGRCAEGRHMTKEAIEKIAEGRVWTGEAAKELGLVDELGGIDKALDIAVAKADIENYTVISYPAKQDFFSSLFETKPTNYVEAQLLKSKLGEYYRQFDLLKNLQEKSMIQARVPFELNIK | signal peptide peptidase SppA | N | N | 1 | 1 | Cyto. Membr. | M | 0 | 1074 | 100 | 87.5 | 47 | Core |
| **Protein Turn Over** | | | | | | | | | | | | | | | | | | |
| M38 | M38.UNW | Non-peptidase homologues | *B. thetaiotaomicron* | The beta-aspartyl dipeptidase is a cytosolic enzyme and is a product of the iadA gene in Escherichia coli. Its function is the release of isoaspartate residues from peptides which accumulate during the stationery phase of bacterial growth and may be toxic. However, isoaspartates do not accumulate in iadA knockouts, probably because another enzyme is capable of releasing them from peptides (Gary & Clarke, 1995). Isoaspartates can also be metabolized by L-asparaginase (T02.002, Kelo et al., 2002), which also hydrolyses asparagine, though kinetic analysis suggests that the isoaspartyl dipeptidase activity is the major activity (Borek et al., 2004. The structure of L-asparaginase has been determined, showing that it is a N-terminal nucleophile hydrolase, unrelated to enzymes in family M38 (Prahl et al., 2004), which processes itself by cleavage of a normal peptide bond (Hejazi et al., 2002. | MLTQIINGRILTPQGWLKDGSVLICDGKILEVTNSDLAVIGATVIDARGMTIVPGFVSMHAHGGGGHDFTEATEEAFRMATTAHLKHGATSMFPTLSSTSFEKLYQAVDVCEGLMQEPDSPILGLHIEGPYLNPKMAGSQYEGFLKTPDENEYIPLLEHTTCIKRWDISPELRGAHDFAKYTRSKGIMTAVTHTEAEYDEIKAAYAVGFSHAAHFYNAMPGFHKRREYKYEGTVESVYLTDGMTVEVIADGIHLPATILKLVYKLKGVENTCLVTDALAYAAYEGHEPIDSRYIIEDGVCKMADHSALAGSLATMDVLVRTMVKKANIPLEDAVRMASETPARLIGVSDRKGALAKGLDADIVILDKELNVRCVWSMGKIVPGTDVLLHK | N-acetylglucosamine-6-phosphate deacetylase | N | N | 0 | 0 | Cyto. | Ic | 0 | 768 | 100 | 94.615 | 47 | Core |
| M38 | M38.UNW | Non-peptidase homologues | *B. thetaiotaomicron* |  | MKRTLIQNAVIVNEGRKVLGSVVIEGEKIAEILTGEEKTTTPCDEIIDATGCYLLPGAIDEHVHFRDPGLTHKADITTESRAAAAGGVTSIMDMPNTNPQTTTLEALDEKLTLLAGKSAVNYSCYFGATNNNYPQFAQLDKHRVCGVKLFMGSSTGNMLVDRMASLRNIFGGTDLLIAAHCEDQGIIKENTDKYKKAYGDDVPLTLHPVIRSEEACYRSSELAVQLAREANARLHIMHISTARELDLFSDAPLITKRITAEACVSHLLFTEDDYQTLGARIKCNPAIKTAEDRKALQEAVNSGLIDAIATDHAPHLLSEKEGGALKAMSGMPMIQFSLASMLELVDKGIFSIEKIVEKMSHAPAQMYEINNRGFIHKGYQADLVLVRPNSKWTVTTDCIVSKCQWSPLEGHTFNWKVEKTFVNGHLLYDNGKIDETYRGQELRFR | dihydroorotase | N | N | 0 | 0 | Cyto. | Ic | 0 | 830 | 100 | 88.539 | 47 | Core |
| M38 | M38.UPW | UP | *B. thetaiotaomicron* |  | MLTQIINARILTPQGWLKDGSVLIRDNKILEVTNCDLAIIGAKLIDAKGMYIVPGGVEIHVHGGGGRDFMEGTEEAFRVAVKAHMQHGTTSIFPTLSSSTIPMIRAAAETTEKMMAEPDSPVLGLHLEGHYFNMAMAGGQIPENIKNPDPEEYIPLLEETRCIKRWDAAPELPGAMQFGKYITAKGVLASVGHTQAEFEDIQTAYEAGYTHATHFYNAMPGFHKRKEYKYEGTVESIYLIDDMTVEVVADGIHVPPTILRLVYKIKGVERTCLITDALACAASDSQTAFDPRVIIEDGVCKLADRSALAGSVATMDRLIRTMVQKAEIPLADVIRMVSETPARIMGVLDRKGTLERGKDADIIALDRDLNVRAVWAMGQLVEGTNKLF | N-acetylglucosamine-6-phosphate deacetylase | N | N | 0 | 0 | Cyto. | Ic | 0 | 782 | 100 | 96.907 | 47 | Core |
| S16 | S16.UNW | Non-peptidase homologues | *Capnocytophaga ochracea* | The function of Lon peptidase in bacteria is thought to be the degradation of unfolded proteins (Roudiak et al., 1998). One physiological substrate of Lon peptidase in E. coli is SulA protein, which is a particularly unstable protein that is a cell-division inhibitor (Nishii et al., 2002). | MLIKVFGAAVQGIDATLITIEVNSSRGCMFYLVGLPDSAVKESHQRIISALQVNGYKMPTTNIVVNMAPADIRKEGSAYDLPLAIGLLGASETISSEKFSRYLLMGELSLDGSIQPIKGALPIAIKAREDGFEGLIIPQQNAREAAVVNQLKVYGVSNIREVIEFFNNERELEPTVVNTREEFYAHQSTFEFDFADVKGQENVKRALEVAAAGGHNLIMIGAPGSGKSMMAKRLPSILPPLSLGESLETTKIHSVAGKLNRNSSLITQRPFRDPHHTISQVAMVGGGSFPQPGEISLAHNGILFLDELPEFNRNVLEVLRQPLEDRRITISRIKSSIDYPASFTLVASMNPCPCGYYNHPTKACVCSPGQVQKYLNKISGPLLDRIDIQIEIIPVPFDKISDQRRGESSETIRERVIKARQIQEKRYAEYPGIYCNAQMNSKLLSLFARPDDKGLTLLKNAMDRLNLSARAYDRILKVSRTIADLEGSEQILSGHLAEAISYRNLDRENWAG | magnesium chelatase | N | N | 0 | 0 | Cyto. | Ic | 0 | 717 | 100 | 65.625 | 45 | SoftCore |
| S16 | S16.UPW | UP | *B. fragilis* |  | MKERYLLEDVGDNNGFSLITDYDGNDEQAFEVNVKSGEILPVLPLRNMVLFPGVFLPITVGRKSSLKLIRDAEKKHKDIAVVCQRSAHTEDPKLEDLHNVGTVGRIVRVLEMPDQTTTVILQGMKRLILKDITETHPYLKGEIELLEEDVPSKDDKEFQALVETCKDLTMRYIKSSDVMHQDSAFAIKNINNSMFLVNFICSNLPFKKDEKMDLLSINSLRERTYHLLEILNREVQLAEIKASIQMRAREDIDQQQREYFLQQQIKTIQDELGGSGQEQEIEEMRQKAERMRWNAEVRETFLKELAKLERTHPQSPDYSVQLNYLQTMLNLPWGVYTTDNLNLKNAEKTLNKDHYGLEKVKERILEHLAVLKLKGDMKSPIICLYGPPGVGKTSLGKSIASALKRKYVRMSLGGVHDEAEIRGHRKTYIGAMPGRIIKSLIKAGASNPVFILDEIDKVSADRQGDPSSALLEVLDPEQNTSFHDNFLDVDYDLSKVLFIATANNLNTIPGPLLDRMELIEVSGYITEEKIEIARKHLLPKELEANGLKKSDIKLPKETLEAIIESYTRESGVRELEKKIGKILRKSARQYATDGYFAKTEIKPTDLYDFLGAPEYTRDKYQGNDYAGVVTGLAWTAVGGEILFVETSLSRGKGGRLTLTGNLGDVMKESAMLALEYIKSHASLLQLNEDIFDNWNIHIHVPEGAIPKDGPSAGITMATSLASALTQRKVKANIAMTGEITLRGKVLPVGGIKEKILAAKRAGIKEIIMSVENKKNIDEIQDIYLKGLTFHYVNDIKEVFAIALTNEKVADAIDLSVKKPSQE | endopeptidase La | N | N | 0 | 0 | Cyto. | Ic | 0 | 1516 | 99 | 88.564 | 47 | Core |
| S09X | S09.UPW | UP | *B. fragilis* | [Many peptidases in the family are believed to be important for the degradation of biologically active peptides: for example, DPP-IV metabolizes the insulinotropic hormone, glucagon-like peptide 1 (Holst & Deacon, 1998; Deacon & Holst, 2002). Members of the family are found in different cellular locations: prolyl oligopeptidase in intracellular, DDP-IV and fibroblast activation protein α (S09.007) are expressed on cell surfaces; and oligopeptidase B is secreted to the bacterial periplasm (Tsuru, 1998).](https://www.ebi.ac.uk/merops/cgi-bin/refs?id=20040510N955) | MKKRNLLLAALLLILVAPSFAAKVDTLLVNSPSMNKDVQVVVVTPDAALGKKAVACPVIYLLHGYGGNAKTWIGVKPDLPQIADEKGIIFVCPDGKNSWYWDSPKDPSYRYETFVSSELVKYIDGHYKTIADRKGRAITGLSMGGHGAMWNAIRHKDTFGAGGSTSGGVDIRPFPLNWDMSKQLGEFAYNKKVWDEHTVINQIDKIENGDLAIIIDCGEADFFLNVNKDLHNRLLARKINHDFITRPGGHNGKYWNNSIDYQILFFDKFFKK | Hyp. prot. | Y (SP) | Y | 0 | 0 | ExtraceL. | Exp. | 1.72E-168 | 467 | 99 | 81.481 | 13 | Shell |
| S09X | S09.UNW | Non-peptidase homologues | *B. vulgatus* |  | MVNTAVTEQTGKKVSKLLKTNHRMLNPISPPGVYIADPEARQMPDGRVYVYGSRDEPTNVWCSHTYDVLSTSDLINWDVEQFSFATKGIGKQVDYTDQLLYAPDCIYHNGKYYLYYCLTNEKEDEGVAVSSSPYGPFKEGKAIAGIHGIDPSVFIDDDGQAYLFWGQANAKGAKLSKDMLSIEGEVHEKLLTYKEHAFNEGSSVRKRNGIYYYVYAGHQRHGESNCATLNYATATSPLGPYTYRGVIIDNWGSDRNLVNNHGSIAEIDGQWYIFYHRPTHASSSMRKVCMEPIVFNPDGTINEVEMTTQGCGGPINPLLRMDAARACLLSGHTTVVVRRPAHSNPVEYLSAIRNGDCAYWKYFDFTGMEVNSFICKTWGKNAAGTIEIRLDKPDGELIGTCELQPMNGDVAYSVHRTKVKPVTGVHALVLVFKNNDPSMTGQDFMNLEWFMFENHDR | Hyp. prot. | N | N | 0 | 0 | Cyto. | Ic | 2.07E-51 | 187 | 97 | 31.368 | 47 | Core |
| S09X | S09.UPW | UP | *B. thetaiotaomicron* |  | MAFAQQPVESTSNESDPMLRIGKSYLGTRYVANTLDRNEVEQLVINTKEVDCLTFVEYTLAQALGSSFADNLQRIRYRDGIINGYPSRLHYTSDWIDNGLRNGFLTDVTARNSSRTTKLSLSYMSTHPKQYKKLADSPENVTQMAEYEKALSGKTVSWLPKSELPENGLPWIMNGDIIAITTKLPGLDIAHVGIAYYVNGKLHLLHASSTLGKVVVSEKPFNHMLNNNKSWTGIRVVRMSHSKNN | xylanase | Y (SP) | N | 0 | 0 | Unknown | Exp. | 1.32E-132 | 384 | 93 | 78.07 | 1 | Cloud |
| S09X | S09.UPW | UP | *B. vulgatus* |  | MPVSIDIACRTLAYYDVANLAQKIKVPGFYSYGYNDNTCPPTTVTAALNVITAPKTIVVTPVSAHWRFEETNRKSIEWMKKRIN | Hyp. prot. | N | N | 0 | 0 | Unknown | Ic | 1.62E-20 | 84,3 | 88 | 48.649 | 47 | Core |
| S09B | S09.UPB | UPs | *B. vulgatus* |  | MNPYEIIDKYYPENTEQRQILVIHSLSVAGKAMKILDAHPELRLNRSFVKEAALLHDIGIFQTNAPTIQCFGSHPYIAHGYLGAEILREEGFPQHALVCERHTGAGLSLQDIVDQQLPVPHREMLPITLEEQLICFADKFFSKTHLDEEKSVEKARHSIAKYGEEGLSRFDRWCSLFL | phosphohydrolase | N | N | 0 | 0 | Cyto. | Ic | 3.18E-174 | 522 | * | 38.815 | 4 | Cloud |
| S09C | S09.B04 | *NaN* | *B. thetaiotaomicron* |  | MRKRIIYVIIIVVLLLTGCTIGGSFYMLNFSLTPDAKILSKDADSYPYMYRNYPFLRPWVDSLRQADALKDTFIINPHGIQLHAYYVAAPKPTDKTAVIVHGYTDNAIRMFMIGYLYNRDLGYNILLPDLQHQGESEGRAIQMGWKDRFDVLQWMNIANKIFGDSTQMAVHGISMGGATTMMVSGEEQQPFVKCFVEDCGYTSVWDEFSHELKSSFFLPPFPLMYTTSWLCEKKYGWNFKEASSLKQVAKCKLPMLFIHGDKDTYVPTWMVYPLYEAKPEPKELWIVSGAAHAVSYQENKQEYTDKVRDFVGRYIH | membrane protein | Y (Lipo) | N | 0 | 1 | Cyto. Membr. | M | 0 | 575 | 92 | 92.414 | 2 | Cloud |
| S09B | S09.013 | dipeptidyl-peptidase 4 | *B. thetaiotaomicron* |  | MRKVSLALLFCLLCLTGMAQGQKALDLKDITSGRFRPENIQGVIPTPDGEYYTQMNGEGTQIIKYSFKTGEKVEVVFDVNTARECDFKHFDSYQFSPDGQKLLIATKTTPIYRHSYTAVHYIYPLKRNDKGVTTNNIIERLSDGGPQQVPVFSPDGTMIAFVRDNNIFLVKLLYGNSESKVTEDGKQNSIINGIPDWVYEEEFGFNRALEFSADNTMIAFIRFDESEVPSYSFPIFAGQAPYINALKDYPGEYTYKYPKAGYPNSKVEVRTYDIKSHVTRTMKLPLDTDGYIPRIRFTKDASKLAIMTLNRHQDRFDLYFADPRSTLCKLVLRDESPYYIKENIFDNIHFYPDYFSMLSERDGYSHLYWYSMGGNLIKKVTNGKFEVKDFLGYDEEDGSFYYTSNEESPLRKAVYKTDKKGKKTKLSQQTGTNTPLFSKSMKYYMNKYSSLDTPMQITLNDNTGKTLKTLVTNDQLKQKLAGYAVPKKEFFTFQTTDGVTLNGWMMKPVNFSASKKYPVLMFQYSGPGSQQVLDTWGISWETYMASLGYLVVCVDGRGTGGRGEAFEKCTYLKIGVKEARDQVETALYLGKQAYVDKDRIGIWGWSYGGYMTLMSMSEGTPVFKAGVAVAAPTDWRFYDTIYTERFMRTPKENAEGYKESSAFTRADKLHGNLLLVHGMADDNVHFQNCTEYAEHLVQLGKQFDMQVYTNRNHGIYGGNTRNHLYTRLTNFFLNNL | peptidase S9 | Y (SP) | Y | 0 | 0 | Unknown | Exp. | 0 | 1414 | 100 | 93.478 | 47 | Core |
| S09 | S09.UPD | UP | *Chitinophaga_pinensis* |  | MKGEDKMKQTIMGIGLIVGVLVGFVPSVDAQTQKKPVDIEACMSWKRVESPDISPTGRWVTYRIAPMEYNPENTDAKTVHLFDTRTRKEILLDDVENIEFYNSDQALSYQKADSTGNMKTILMELPSGIKKEWKYKESFRPVNGTPYSVSVTNVPKDTVNHVPSFNRLVVRHLKTGTAFQIDSIGYYTLYNEGRSIIFVRRQAKGNALCYGPLTGPYQTIYQSAVKKEPVSFSLDTKLMTGEFSIKDSLWYNFSLKKNTCDLVFDRKEVILPAGMELARATLSHSQKFLTMELRPYQEKVNKDKKEEEVKPDKSFELELWTWDEYEVPTLQTRGRYFRPQYSKYIYDIASGKLTEVAPGHADLLEPDRAEEIHYVLYTDETPYRMQKEWLNEVPFDIYSVNVHTGKKQLVGRSYRTRPKWSMNGKWAVMYDPVAQVWNKFDGTTGKVTDISTAIGYPVFEETYDKPNPAPAYGIAGWTADGNNVLIYDAYDWWKIDLTGERQPECFTKGYGRKNKRSIRKMTSNIDKEVFKPDETVVVSVWDENTMDEGIYSLDMKGRLKKLAEGPYIYAIHRFSDNQKYCIWNRQNMSEFRDLWWSKADFSDPIRITNANPQQSEYKWGTAKLVEWTNYENKPNKGILYLPEDYDPQKEYPVLVQFYETHSGGLNTYHAPMLSSAMGDVMYFVSNGYIVFMPDVHFTIGTPGQSCYDAVVSGTKYLIEQGIAHPGKIGLQGHSWSGFQTSYLVTKTDLFTCANIGAPITDMVTGYLGIRGGSGLPRYFMYEEWQSRMGKNLWEAKDKYLANSAIIEADKIHTPLLIWHNDKDEAVAYEQGRALYLAMRRLQRPAWHLNYKGEGHFLSNQAAQKDWTIRMMQFFDYYLKGTKEPRWMKEGIHLRERGIDQKYDLLEK | peptidase S9 | Y (SP) | Y | 0 | 0 | Unknown | Exp. | 0 | 631 | 99 | 39.102 | 13 | Shell |
| S09X | S09.UNW | Non-peptidase homologues | *B. thetaiotaomicron* |  | MKRILLLTAVFIMMLGTNFSSAQTDADNFYKSDLVSVEKVSFSNQYKMKVAGNLFLPKNMKEGDRYPAIIVGHPMGAVKEQSANLYATKMAERGFVTLSIDLSFWGGSEGEPRNAVLPEVYAEDFSAAVDFLGTRPFVDRNLIGVIGICGSGSFAISAAKIDPRLKAIATISMYNMGTASRNGLKHSLTLEQRKQIMAEAAEQRYVEFLGGETKYTGGTVHELTEKSGPIEREFYEFYRTQRGEFTPEGATPLTTTHPTLSSNVKFMNFYPFADIETISPRPMLFITGENAHSREFSEDAYRLAAEPKELYIVPGAGHVDLYDRVSLIPFSKLESFFKEYLKK | membrane protein | Y (SP) | Y | 0 | 0 | Cyto. Membr. | Exp. | 0 | 648 | 100 | 93.878 | 1 | Cloud |
| S09 | S09.UPB | UP | *B. _vulgatus* |  | MVLKQLFIAGSLSFTLALQGWGQVPAEHDTVQANYELAEKFHEFTLGGKLSNNSMSLYPHEINDTDNFWFDFTTSAGKHYYYVNPKEGKKELLFDNEEMAMLLSGLTHEVVNPVRLDLSELKFAKDQKSFVFSYRSKKYDYNRITRKLKEVEEKKADDRDAEPIYSWMNFSPDKKYILYAKDHNLYVKGNKALGVDTTEVQLTTDGVPDFSYAREEDAGENGEVPSNARWCPDSRHAYIVLDDNRKLRDFWVINSISDKPELKKYKYEFPGDKYVTQNELVIIDIVERTARKAKIQKWNDQYVMPFSVTSDSKYVFFERTKRTWDEVDVCSVNTSTLEVKELIHEVDKPYRDPHARSVEVLNDGKDILFRSERTGWGHYYHYDGQGNLKNAISSGPWVSGHIAAIDTLQRTVYFYGYGDDPKINPFYYRLYKSNMDREGATLLTKEDGQHRVIFLKSRRYFIDTFSRVDMEPKILLKDNTGKVIMELAKPDLSQVYAAGWKKPENFVVKAADNVTDLYGVMWKPSNFDPEKKYPIISVVYPGPYFGFVPTNFTLDDSYCTRMAQLGFIVITVGHRGDTPMRGKAYHRYGYGNMRDYPLEDDKYAIEQLAQRHSFIDGKKVGIYGHSGGGFMAAAAICTYPDFYKAAVSCSGNHDNSIYNRGWGECYNGVREVEKVVKDSLGNETKEYEYKFRVKSNAEIAKNLKGHLMLVTGDMDKNVNPAHTYRMAQALIEAGKDFDMLVIPGAGHGYGSADEYFEKKMYRFFAKHLLGDTRADYWGDINRSK | peptidase S9 | Y (LIPO) | Y | 0 | 0 | Cyto. | Exp. | 0 | 578 | 98 | 40.727 | 7 | Cloud |
| S09B | S09.017 | prolyl tripeptidyl peptidase | B. thetaiotaomicron |  | MSKVGKRAFLLLLAIPIVINVMAQETKKPTLEDLIPGGESYRYAENLYGLQWWGDECIKPGIDTIYSIQPQIGKETMLITREQINKALAEDKSGKLSHLYSVRFPWAEKPQMLLNIAGKYIVYDFKNNRIVSSRKPKAKAENEDYCTANGNVAYTIGNNLYVNEQAVTNEPEGIVCGQSVHRNEFGINKGTFWSPKGNLLAFYRMDESMVTQYPLVDITARVGEVNNVRYPMAGMTSHQVKVGIYNPATGKSIYLDAGDPTDRYFTNISWAPDEKSLYLIEVNRDQNHAKLCQYNVETGKLTKVLYEETHPKYVEPQNPIIFLPWDTSKFIYQSQRDGYNHLYLFDTKASIYGDLQEGTGGAQYRESVKVKQLTKGNWLVKNILGFNAKRKEVIFTAIEGLRSGHFAVNVNNGKMSCPFDSSKESEHNGVLNASGTFLIDRYSTKDQPRIINLVDTKNFKETVNLLTAKDPYEGYQMPSIETGTIKAADGTTYLHYRLMKPANFDPNKKYPTIIYVYGGPHAQCVTGGWQNGARGWDTYMANKGYIMFTIDNRGSSNRGLEFENATFRRLGIEEGKDQVKGVEFLKSLPYVDGGRIGVHGWSFGGHMTTALLLRYPEIFKVGVAGGPVIDWGYYEVMYGERYMDTPETNPEGYKECNLKNLAGQLKGHLLIIHDDHDDTCVPQHTLSFMKACVDARTYPDLFIYPCHKHNVSGRDRVHLHEKITRYFEQNL | prolyl tripeptidyl peptidase | Y (SP) | Y | 0 | 0 | Mul. loc. | Exp. | 0 | 1265 | 100 | 81.446 | 47 | Core |
| S09 | S09.UPB | UP | *B. _vulgatus* |  | MKNSLFRYLCLAVALVSCNLAGAQQKANYQLAEKFRLLEQNPIDKYSTEVRPTFINDTDCFYYSFTTREGKKYYYVNPKKKEKRLLFDTDELLSKIAVYTKKAYSSADPHLSFTFMKDNETIRIDFDRGLYTYNIHTKELKQLDEKPTYKTGDPYWMKYSPDSLYFLYASKDNLYFVGNPKKGQDTIPVQLTTDGMPDYTFNREDEGKMEGRFGAESAHWIPGSHRFYAVREDNRKVRDLWVINSLSKPSPELITYKAELAGDKNVTQYELLLGDIDKREVKKVDINRWPDQYIDVLYASKDGKRLYFQRYNRTWNQSDICEVDVETGKVRVVIHEENKPYLDYQMRNVSFLNDGKEILFRSERNGWGHYYLYDTATGNLKNQLTDGTWVAGPVAQIDTVGRKMYFYGYGREKGIDPYYYILYEAHLDRPNALRLLTPENASHEVSISPSCRYLVDSYSTVSQEPVNVVRNRNGKVIMTLEKPDLQPVYEMGWKAPERFKVKAADGVTDLYGVMWKPADFDSTKVYPIISNVYPGPFFEYVPTRFTINDVYNTRLAQLGFIVITVGHRGGTPMRGKAYHTYGYNNMRDYPLADDKYAIEQLAARYPFIDATKVGIYGHSGGGFMSAAAICTYPDFYSAAVSSAGNHDNRIYNKGFVEIHYGVDEKVTTTKDSLGVESKTYNYSVRVRPNQELAKNYKHGLLLFTGAIDQTVNPANTLRLVDALIKADKDFDMFVLPKCTHGFFGESESFFEHKMWRHFARLLLHDNSADCDIDLNKDMIKDERRR | peptidase S9 | Y (SP) | Y | 0 | 0 | Mul. loc. | Exp. | 3.18E-174 | 522 | 98 | 38.815 | 3 | Cloud |
| S09C | S09.UPC | UPs | *B. thetaiotaomicron* |  | MKKKEVALAIFLLTGLTLQAQERVTQYKVRDAIEVRTPIMNDSINPKGEKHSTKMLLKTPVVLDLPDAPLQSLTVDTAGYLTLDKADKNSKIYVLKTQIRAERFLKGKLKVTSPVRWEVFIDEVSKQTKDAAEDSISSASSRDIALTLEPERDYEITIKLLSTAEDKAAPTLKCEFIKDNKFKDIACTLDPNAKKRFSLDNTVYGNRVISVAISPSGKYLLTRYWNNHAAKRSRTYCQLTELKTGKVLLDNARDGMRWMPKSDKLYYTVTALSGNDVITLDPATLNEETLLKGIPEQSFTWSPNEDFLIYYPREEGEKEQGALRRIVSPADRIPNTRGRSFLAKYNIANGVSERLTYGNHSTYLQDISPDGKFMIYSTSKENITQRPFSLSSLYLVDLETLKVDTLFHDERFLGGASYSPDGKQLLLTASPEAFGGIGKNCGNHPIANDFDTQAFIMDLATRKIQPITKDFNPTVSPLQWNRGDGCIYFNTDDGDCKNIYRYSPKNNSFEKLNLETDVTSAFTLSEYNPGIAAYIGQSDHNAGVAYLYDMKKKTSRLLADPLKPILDQIELGKTEPWNFTASDGTVITGKMCLPPNFDPNKKYPMLVYYYGGTTPTTRGISNPYCAQLFASRDYVVYVIQPSGTIGFGQEFSARHVNAWGKRTADDIIEGTKQFCKEHPFVDEKRIGCLGASYGGFMTQYLQTQTDIFAAAVSHAGISDVTSYWGEGYWGYSYNAIAAADSYPWKNPDLFTKQGSLFNADKINTPLLLLHGTVDTNVPIGESIQLFNALKILGKTVEFITVDGENHFISDYDKRIKWHNSIMAWFARWLQDRPEWWNEMYPERHL | peptidase S9 | Y (SP) | Y | 0 | 0 | Unknown | Exp. | 0 | 1607 | 99 | 89.881 | 47 | Core |
| S09D | S09.UPD | UPs | *B. thetaiotaomicron* |  | MRQANLFMMSAAMLLAACGGTKDAGKTDQVLIEKSNIKIEGKRMTPEALWAMGRIGGFAVSPDGKKIAYTVAYYSVPENKSNREVFVMNADGSDNQQITRTPYQENEVTWIKGGTKLAFLSNDNGSSQLYEMNPDGSERKQLTNYDGDIEGYSISPDGKKLLFISQVKTKESTADKYPDLPKATGIIVTDLMYKHWDEWVTTAPHPFIADFDGNGISNVVDILEGEPYESPMKPWGGIEQLAWNTTSDKVAYTCRKKTGLEYAISTNSDIYVYDLNTKETKNITEENKGYDTNPQYSPDGKYIAWQSMERDGYEADLNRLFIMNLETGEKRFVSKAFESNVDAFVWGADAKTIYFTGVWHGESQIYALNLANDSVKAITSGMYDYEGVALFGDKLIAKRHSMSMGDEIYAVALDGLATQLTQENKLIYDQLEMGKVEGRWMKTTDGKQMLTWVIYPPQFDPNKKYPTLLFCEGGPQSPVSQFWSYRWNFQIMAANDYIIVAPNRRGLPGFGVEWNEQISGDYGGQCMKDYFTAIDEIAKESYVDKDRLGCVGASFGGFSVYWLAGHHDKRFKAFIAHDGIFNMEMQYLETEEKWFANWDMGGAYWERQNPVAQRTFANSPHLFVEKWDTPILCIHGEKDYRILANQAMAAFDAAIMRGVPAELLIYPDENHWVLKPQNGVLWQRTFFEWLDKWVKKAPSE | peptidase S9 | Y (Lipo) | Y | 0 | 0 | Cyto. | Exp. | 0 | 1413 | 100 | 96.143 | 47 | Core |
| S09D | S09.UPD | UPs | *Chitinophaga pinensis* |  | MNQLAVGLFLFLSSLFSCQQKGDFKSMNVDDFDNLIQNEDIQRLDVRTLAEYSEGHIAKTININVMDDSFASMADSLLQKDKPVAVYCRSGKRSKKAANILSEKGYKVFELDKGFNSWQEAGKEIEH | rhodanese | Y (Lipo) | Y | 0 | 0 | Cyto. | Exp. | 0 | 631 | * | 39.102 | 47 | Core |
| S14 | S14.001 | peptidase Clp | *B. thetaiotaomicron* | Clp peptidases have important roles in the both protein quality control and the regulatory degradation of specific proteins. The ClpXP and ClpAP complexes are responsible for the degradation of nascent polypeptides whose synthesis is interrupted by pauses in translation caused by damage to the mRNA or by deficiencies in the components required for translation. When a pause in translation occurs an 11 amino acid peptide (AADENYALAA), referred to as the SsrA-tag, is co-translationally added to the C-terminus of the growing chain (Keiler et al., 1996). The SsrA-tag is recognised by the ATP-binding components of the Clp complex, targeting the protein for degradation (Gottesman et al., 1998). A specificity-enhancing factor, SspB, has been identified that increases the rate of degradation of SsrA-tagged proteins (Levchenko et al., 2000). A role for ClpAP has also determined in the targeting of proteins with hydrophobic or basic amino acids at the N-terminus, which is rare in normal cell proteins (Tobias et al., 1991). | MDDFRKYATKHLGMNGMVLDDVIKSQAGYLNPYILEERQLNVTQLDVFSRLMMDRIIFLGTQVDDYTANTLQAQLLYLDSVDPGKDISIYINSPGGSVYAGLGIYDTMQFISSDVATICTGMAASMAAVLLVAGKEGKRSALPHSRVMIHQPMGGAQGQASDIEITAREIQKLKKELYTIIADHSHTDFDKVWADSDRDYWMTAQEAKEYGMIDEVLIKK | ATP-dependent Clp protease proteolytic subunit | N | N | 0 | 0 | Cyto. | Ic | 3.65E-164 | 452 | 100 | 99.545 | 47 | Core |
| M48B | M48.004 | HtpX-2 peptidase | *B. thetaiotaomicron* | In bacteria, the HtpX endopeptidase is induced by heat shock and may be involved in the degradation of abnormal proteins because these accumulate in htpX mutants (Kornitzer et al., 1991. | MQYVGIQTQQNRNNLRSGFLLLLFPCLVTALLYLSCYLLVLFGYGKSMEIEMMPMVNHFFLSSLPYTLGVVVIWFLIAYWANTSIINSATCSKPLDRKENKRVYNLVENLCMSQGMSMPKINIIYDDSLNAFASGINDRTYTITLSRGIIQKLNDEELEAVIGHELTHIRNRDVRLLIISIVFVGIFSMLTEITFYAITHIRVRSNSKGSGGIFIFIFIALLIAAIGFLFASLMRFAISRKREYMADAGSAEMTKNPLALASALRKISADPAIEAVQRKDIAQLFIQNPKKKTKGLYSKFSGLFATHPPIEKRIEILEQF | Hyp. prot. | N | N | 4 | 4 | Cyto. Membr. | M | 4.54E-173 | 483 | 100 | 78.125 | 47 | Core |
| M48C | M48.UPC | UP | *B. thetaiotaomicron* |  | MKRQIAMIALLFLGMGMTASAQFGKKFNVGKALQAGKDVVSAVTLSDADIANMSKEYMVWMDAHNPLTKPDTEYGKRLEKLTGHIKEMDGLKLNFGVYEVVDVNAFACGDGSVRICAGLMDVMTDEEVMAVVGHEIGHVVHTDSKDAMKNAYLRSAVKNAAGAASNKVAKLTDSELGAMAEALAGAQYSQKQENEADEYGVEFCVKNNIDPYAMANALTKLAELAKDAPKSSYVQRMFSSHPDTAKRIERAKAKADSYKK | peptidase M48 | Y (SP) | Y | 0 | 0 | Mul. loc. | Exp. | 3.56E-159 | 442 | 100 | 81.609 | 47 | Core |
| S41A | S41.UPA | UP | *B. thetaiotaomicron* | The bacterial C-terminal processing peptidase-1 (S41.001) is believed to be important for the degradation of incorrectly synthesized proteins. Proteins synthesized from damaged mRNA are tagged with the tripeptide Leu-Ala-Ala at the C-terminus, which is the target for degradation by the C-terminal processing peptidase. | MKKLLSGRIAIVVVAVIATVAFFSFKSGDDRNFQIAKNLDIFNAIVKELDMFYVDTIDPNKTIREGIDNMLFTLDPYTEYFPEEDQSELEQMIKGSFGGIGSYIAYNTKLKRSMISEPFEGTPAAKAGLKAGDILMEIDGKDLAGKNNAEVSQMLRGQAGTSFKLKVNRPNAKGGNTPMEFTIVRESIQNPAIPYAAVLDNQVGYINLSTFSGNPSKEFKKAFLDLKKQGATSLVIDLRGNGGGLLDEAVEIANYFLPRGKVIVTTKGKIKQASNTYKTLREPLDLDIPIAVLVNSGTASASEILSGSLQDLDRAVVVGSRTFGKGLVQVPRSLPYGGTMKVTTSKYYIPSGRCVQAIDYKHRNEDGSVGTIPDSLTKVFYTAAGREVRDGGGVMPDVAIKQEKLPNILFYLVRDNLIFDYATQYCLKHPTIVEPEKFEVTDADYNDFKTLVKKADFKYDQQSEKILKTLKEAAEFEGYMEGASEEFKALEKKLNHDLERDLDYFSGDIKKMIASEIIKRYYYQRGNIIQQLKDDDGLKEAVKILNDPAKYKEMLSAPVAKK | peptidase S41 | N | N | 0 | 1 | Cyto. Membr. | Ic | 0 | 1048 | 99 | 90.374 | 1 | Cloud |
| S41A | S41.UPA | UPs | *Leptospira interrogans* |  | MSVIILGIESSCDDTSAAVIKDGYLLSNVVSSQAVHEAYGGVVPELASRAHQQNIVPVVHEALKRAGVTKEELSAVAFTRGPGLMGSLLVGVSFAKGFARSLNIPLIDVNHLNGHVLAHFIKAEGEENRQPNFPFLCLLVSGGNSQIILVKAYNDMEILGQTIDDAAGEAIDKCSKVMGLGYPGGPIIDKLARQGNPKAFTFSKPHIPGLDYSFSGLKTSFLYSLRDWLKEDPDFIEHHKVDLAASLEATVVDILMDKLRKAAKEYKIKEVAVAGGVSANNGLRNAFREHAEKYDWDIFIPKFSYTTDNAAMIAITGYFKYLDRDFCSIDLPAYSRVTLE | tRNA N6-adenosine(37)-threonylcarbamoyltransferase complex transferase subunit TsaD | N | N | 0 | 0 | Extracel. | Ic | 3.32E-58 | 201 | 94 | 38.066 | 47 | Core |
| S41A | S41.UPA | UP | *B. thetaiotaomicron* |  | MSTKNSSRFTPVIIAISVVIGILIGTFYAKHFAGNRLGIINGSSNKLNALLRIVDDQYVDTVNMTDLVEKAMPQILAELDPHSTYIPAQNLEEVNSELEGSFSGIGIQFTIQNDTIHVNAVIQGGPSEKVGLMAGDRIVTVDDSLFVGKKVTNERAMRTLKGPKGSQVKLGVKRMGEKDLLSFTITRGDIPQNTVDAAYMLNDEIGYVKVSKFGRTSHVELLNALAQLNHKKCKGLIIDLRGNTGGYMEAAIRMVNEFLPEGKLIVYTQGRKYPRAEEFANGTGSCQKMPLVVLIDEGSASASEIFTGAIQDNDRGTVVGRRSFGKGLVQQPIDFSDGSAIRLTIARYYTPAGRCIQRPYESGKDRNYELDLFNRYEHGEFFSRDSIKQNENELYYTSLGRPVYGGGGIMPDIFVPQDTTGVTSYLSTAINRGLTIQFTFQYTDNNRKKLSQYETEEELLNYLRHQGLVEQFVRFAESKGLKRRNILIQKSYKLLEKNIYGNIIYNMLGLEAYLQYFNKTDATVNKGIEILEKGEAFPKAPVAVEEEDTKDKKNEKKKRTAQAYRIVEDPTLYFDYAEASIS | peptidase S41 | N | N | 1 | 1 | Cyto. Membr. | M | 0 | 1103 | 100 | 91.753 | 1 | Cloud |
| S41A | S41.UNA | Non-peptidase homologues | *B. thetaiotaomicron* |  | MTKLRKIILIPALVIVSISGFFSCGVDRWPEYAHMTALDTWMYDIMQQNYLWYQDLPSYDDANLFLEPAQFLSKVKSKNDSYSFVDSVMETPLPSYGFDYSLVRNPDIDTAYNALITYVIPGSPAAAVLKRGDWIVKVDTSYISKKYESQLLQGTEPLEVTLGKYQLVPPTEPPVEGEEEEIYRVVPVGDPVKIGAAVPLVDNPVHCKKTLTLTDGSEVGYLMYNSFTAGTKESPEKYNAELREWSDELAQKNIHEVILDLRYNKGGSIDCVQLLSTMLVSSYYLDQTMGFLEYNDKNTDKDVTLTFDSKLLNTSGGKNLDLTTLIVLIGGETAGAPEMLMHCLNGKIQKLIAIGSSTKGQNVATEQFINEEFQWSVNPVVATIYDSEHDTYPGFKPTYSVNASTDYLTFLPFGDEKETLLSVALGVLNGTYPPKEEEEETPSTRIKIEKSVNSPSSRLFIGGSGLRIK | peptidase S41 | Y (Lipo) | Y | 0 | 0 | Unknown | Exp. | 0 | 555 | 100 | 60.246 | 2 | Cloud |
| S41A | S41.UPA | UP | *B. thetaiotaomicron* |  | MNKITIYVLLACLWTVSAVQAQSFGSPAMRKLQMAEFAISNFYVDKVDEDKLVEEAIIKMLAQLDPHSTYSDAEEVKKMNEPLQGNFEGIGVQFQMIEDTLLVVQPVSNGPSEKIGILAGDRIIAVNDSAIAGVKMSTEEIMKRLRGPKGSKVNLKIVRRGVQDPLVFTVKRDKIPILSLDASYMIQPKTGYIRINRFGATTAEEFKKAMKELQKQGMKDMILDLQGNGGGYLNAAIDLANEFLGQKELIVYTEGRTAKRSDFYAKGNGEFRNGRLIVLVDEYTASASEIVSGAVQDWDRGIIVGRRSFGKGLVQRPIDLPDGSMIRLTIARYYTPAGRCIQKPYDSSTDYNKDLIERFNHGELMNADSIHFPDSLKVQTKKLGRTVYGGGGIMPDYFVPIDTTLYTDYHRNLVAKGAVIKFTMKFIEGHRKELANKYKKFESFNAKFIIDDDMLATLREMGEKEGVKFNEEQYQKSLPLIKTQLKALIARDLWDMNEYFRVMNTTNESVQKALEILNSEEYQKKLRAN | peptidase S41 | Y (SP) | Y | 0 | 0 | Cyto. Membr. | Exp. | 0 | 1019 | 99 | 93.169 | 13 | Shell |
| S41B | S41.UPB | UPs | *B. fragilis* |  | MKKLLLSAFALCFAGTIAAQEAPLWMRYCALSPDGTTIAFTYKGDIYTVPSTGGRATQITTNPAFDTTPVWSPDGKQIAFASDRMGSLDVFVVAKEGGVPQRLTTHSGSEKPVAYKDDKHILFTANIAPSAEDAGFPSGQFQQIYEVAVTGGRPTMFSSMPMECISINKEGIMLYQDKKGYEDYWRKHQVSPIARDIWSYTPGKTPVYQKQTTFGGEDREPVWAPDGKSFYYLSEEKGSFNIFQRTPGANTSKQITFHTKHPVRFLSMASEGKLCYGYNGEIYTLVPGGQPQKVNITILSDKIDKDIIRQIRSYGATDIAVSPKGKEVAFIMRGDVYVTSIDYKTTKQITNTPDQERDISFAPDGRTLVYSSERNGLWQLYTSTIVRKEEKQFTYATELKEECLTKSNVASFQPQYSPDGKEIAFLENRTAIRVINLKSKAVRTVMDAKYQYSYADGDQWFQWSPDSKWILSDFIGIGGWNNKDVVLLKADGKGEMVNLTESGYSDTNAKWVLGGKAMIWNSDRSGYRSHGSWGSESDTYIMFFDVDAYNRFTMSKEDLALLEEAEKTEKEEKAKKEKEEKEKAEKKKDKKDAKTKDTKKEEDKKKEEVKPLTFDLENRFDRIVRLTVNSSRLSDAVLTPKGDVLYYLAAFEGDYDLWEHKLKENTTKILLKGVGGGALIPDKEGKNIFMCTRSQLKKIEIAGSKVTPIEFEAFFDYRPYGERAYIFDHVYQQVNDKFYVADLQGTDWKGYKEAYQRFLPHINNNYDFAEMLSELLGELNASHTGARYAGGGSALSTATLGVFYDESYSGTGLKIKEILDQSPFTQKKTDVKAGCIIEKVDGKTIEANADYFPLFEGKVGRKVILTVYDPATKKRFEETVKAISYGAQSELLYKRWVKRCAQKVEELSGGRIAYVHIKGMDSPSFRKMYSELLGRYRNKEAVIVDTRHNGGGWLHDDVVTLLSGKEYQRFVPRGQYIGSDPFNKWLKPSCMLACEDNYSNAHGTPYVYKTLGIGKLIGTPVAGTMTAVWWERQIDPSIVFGIPQVGCMDMQGKYLENQTLQPDILVYNEPGASLKGEDAQLKAAVNHLLKELSKKK | peptidase S41 | Y (SP) | Y | 0 | 0 | Unknown | Exp. | 0 | 1807 | 99 | 80.201 | 47 | Core |
| S41B | S41.UPB | UPs | *B. thetaiotaomicron* |  | MKKLITSLALALTALSSYAITPLWMRDARISPNGTEIVFCYKGDIYKVPAQGGTAVQLTTQASYEANPVWSPDGKQIAFASDRNGNFDLFIMPADGGTAQRLTYHSASEIPSAFTPDGKFVLFSASIQDPAGSALFPTGAMTELYKVPAGGGRTEQVLATPAEWVCFDKSGKNFLYQDRKGFEDEWRKHHTSSITRDIWLYNTQSGKHTNLTNREGEDRNPVYAPDGNSVYFLSERNGGSFNVYNFSLNTSQTSNVPQEVKTITTFRTHPVRFLSISDKGTLCYTYDGELYTQEANARPKKVNVELVRDDEKEIASLKFSQGATSASVSPDGKQVAFIVRGDVFVTSTDYTTTKQITNTPGKEAAVSFAPDNRTLVYASERTGNWQLYTAKIARKEEANFPNATLIEEEVLLPSKTVERAYPQYSPDGKELAFIEDRNRLMVLDLKTKKVRQVTDGSTWYNTGGGFDYEWSPDGKWFTLEFIGNRHDPYSDIGIVSAQGGTITNLTNSGYISGSPRWVLDGNAILFQTERYGMRAHASWGSQQDVMLVFLNQDAYDRYRLSKEDFELLKELEKEQKKAKDGDKKKDVNKSKKDEADEDKSSEDKADKKDIVVELNGIEDRIVRLTPNSSDLGSAILSKDGENLYYFSAFEGGYDLWKMNLREKDTKRLHKLNTGWASLMLDKKGDIFLLGSRMMQKMDAKSDALKSISYQAEMKMDLAAEREAMFDHVYKQHQKRFYNLTMHGVDWDAMTAAYRKFLPHIDNNYDFAELLSEWLGELNVSHTGGRYSPRGGGDVTSNLGLLFDWNYQEKGMKIAEVIEKGPFDHSRTKVKAGCIIEKINGEELAPDKDITALLNNKAGKKTLVSLYNPQNKERWEEVVMPITSGRLNGLLYKRWVKQRAADVEKWSNGRLGYVHIQSMGDGSFRTVYSDILGKYNNCDGIVIDTRFNGGGRLHEDIEILFSGQKYFTQVVRGREACDMPSRRWNKPSIMLQCEANYSNAHGTPWVYKHQKIGKLVGMPVPGTMTSVSWETLQDPSLVFGIPIIGYRLPDGSYLENSQLEPDIKVANSPETVVKGEDMQLKTAVDELLKEIDSQNR | peptidase S41 | Y (SP) | Y | 0 | 0 | Unknown | Exp. | 0 | 2082 | 100 | 91.336 | 47 | Core |
| **Unknow Function** | | | | | | | | | | | | | | | | | | |
| M93 | M93.001 | *NaN* | *B. caccae* | UNKNOWN | MKNWYIYLLCILVSSNFFACQDDDLEDQSIFDASEKEKSEFDRWLLENYVNPYNIDFKYRMEHIESDYTHNLVPTDFWLSVKLAKIVKHCWLEAYDEVGGLDFTRACAPKVIHLIGSASWDKGTYTLGTAEGGLKVTLYMGNWLDLTNVDRMNEYYFKVMHHEFAHILHQKKNYPVDYDKISAGNYTPTGWQNRKLAEVAPLGFVTPYAGSKPSEDIAEVTACFLTYPEAQWENVMTLAGEKGKPIIDQKLAMVKKYMKDSWQVDLDLLRKVIARRTNEISELDLDHIY | Hyp. prot. | Y (Lipo) | Y | 0 | 0 | Cyto. | Exp. | 8.59E-101 | 297 | 98 | 52.613 | 12 | Shell |
| M49 | M49.003 | dipeptidyl-peptidase IIIB | *B. thetaiotaomicron* | UNKNOWN | MKKHLISMAVTAALLSSCGGAKTTTAEADKFDYTVEQFADLQILRYKVPGFEELTLKQKELIYYLTEAALEGRDILFDQNGKYNLRIRRMLEAVYTNYQGDKTTPDFKNMEVYLKRVWFSNGIHHHYGTEKFVPNFSQDFLKQAVLGIDAQLLPLSDGQTAEQLCAELFPVIFDPAIMPKRVNQADGEDLVLTSACNYYNGVTQKEAEDFYNAMKDPKDETPVSYGLNSRLVKENGKLEEKVWKVGGLYTQAIEKIVYWLKKAETVAENEAQKAVISKLIRFYETGNLKDFDEYAILWVKDLDSRIDFVNGFTESYGDPLGMKASWESLVNFKDIESTHRTEIISSNAQWFEDHSPVDKAFKKDEVKGVSAKVITAAILAGDLYPATAIGINLPNANWIRAHHGSKSVTIGNITDAYNKAAHGNGFNEEFVYSDAEIKLIDTYADLTDELHTDLHECLGHGSGKLLPGVDPDALKAYGSTIEEARADLFGLYYVADPKLVELKLLSSPDAYKAQYYTYLMNGLMTQLVRIEPGNTVEEAHMRNRQLIARWVFEKGAADKVVEMVKKDGKTYVVINDYQKVRELFGELLAEIQRIKSTGDFEGARALVENYAVKVDPVLHAEVLERYKKLNLAPYKGFVNPKYELVTDDNGNITDVTVDYSEGYVEQMLRYSKDYSPLPSVNN | dihydrofolate reductase | Y (Lipo) | Y | 0 | 0 | Cyto. | Exp. | 0 | 1273 | 99 | 90.074 | 4 | Cloud |
| C13 | C13.UPW | UPs | *Pseudomonas syringae* | UNKNOWN | MASNFIKRAITGVLFVAILVGCILYTSFSFGILFIIISALTIYEFGQLVNMRADGVNVNKTIIMLGGAYLFLAVMGFCIDAADSKIFIPYVLLLLYLMISELYLKKENPVLNWAYSMLSQLYIGLPFALLNVLAFHNDPSSEYSSVSYNPILPLSIFIFLWLSDTGAYCIGSLIGKHRLFERISPKKSWEGSIGGGIVAIGSSFILAHYFPFMSMWQWAGLALVVVVFGTWGDLTESLLKRQLHVKDSGTILPGHGGMLDRFDSSLMAIPAAVVYLYALTWF | phosphatidate cytidylyltransferase | N | N | 9 | 6 | Cyto. Membr. | M | 1.38E-32 | 129 | * | 33.086 | 13 | Shell |
| C26 | C26.UPW | UPs | *Gibberella zeae* | UNKNOWN | MLAKRIVPCLDIKDGQTVKGTNFVNLRQAGDPVELGRAYSEQGADELVFLDITASHEGRKTFTELVKRIAANINIPFTVGGGINELSDVDRLLNAGADKISINSSAIRNPQLIDEIAKNFGSQVCVLAVDAKQTEKGWKCYLNGGRIETDKDLFDWTKEAQERGAGEILFTSMNHDGVKTGYANEALAALAGQLSVPVIASGGAGCKEHFRDVFLQGKADAALAASVFHFGEIKIPELKSYLCGEGITIR | imidazole glycerol phosphate synthase cyclase subunit | N | N | 0 | 0 | Cyto. | Ic | 1.19E-42 | 152 | 99 | 30.671 | 47 | Core |
| C26 | C26.UPW | UPs | *Tetraodon nigroviridis* | UNKNOWN | MRNVTLILDDGSRFSGKSFGYEKPVAGEVVFNTAMTGYPESLTDPSYAGQLMTLTYPLIGNYGVPPFSIEPNGLATFMESEKIHAEAIIVSDYSYEYSHWNAVESLGDWLKREQVPGITGIDTRELTKVLREHGVMLGKIVFDDEPDNVLEATYAGVNYVDKVSCKEVIRYNEGADKRKVVLVDCGVKTNIIRCLLKRDVEVIRVPWDYDFNGLEFDGLFISNGPGDPDTCDAAVQNIRKAMKNEKLPIFGICMGNQLLSKAGGAKIYKLKYGHRSHNQPVRMVGTERCFITSQNHGYAVDNNTLGADWEPLFINMNDGSNEGIKHKTNPWFSAQFHPEAASGPTDTEFLFDEFVNLLK | carbamoyl phosphate synthase small subunit | N | N | 0 | 0 | Cyto. | Ic | 9.54E-138 | 416 | 99 | 55.337 | 47 | Core |
| C44 | C44.UNW | Non-peptidase homologues | *B. thetaiotaomicron* | UNKNOWN | MCGIAGILNIKAQTKELRDKALKMAQKIRHRGPDWSGIYVGGSAILAHERLSIVDPQSGGQPLYSPDRKQVLAVNGEIYNHREIRTRYAGKYDFQTGSDCEVILALYKDKGIHFLEDISGIFAFVLYDEEKDEFLIARDPIGVIPLYIGRDKDGKIYFGSELKALEGFCDEYELFLPGHYYYSKEGQMKRWYARDWTEYETVKDNDAKAGDVKEALEDAVHRQLMSDVPYGVLLSGGLDSSVISAIAKKYAAKRIETDGASDAWWPQLHSFAIGLKGAPDLIKAREVAEYIGTVHHEINYTVQEGLDAIRDVIYFIETYDVTTVRASTPMYLLARVIKSMGIKMVLSGEGADEVFGGYLYFHKAPTPQAFHEETVRKLSKLHMYDCLRANKSLSAWGVEGRVPFLDKEFLDVAMTLNPKAKMCPGKNIEKRIVREAFADMLPESVAWRQKEQFSDGVGYSWIDTLKEITTAAVSDEQMENAAGRFPINTPQNKEEYYYRSIFEEHFPSESAARSVPSVPSVACSTAEALAWDIAFKNLNEPSGRAVKGIHEEAYT | asparagine synthase B | N | N | 0 | 0 | Cyto. | Ic | 0 | 1096 | 100 | 94.414 | 47 | Core |
| C44 | C44.UNW | Non-peptidase homologues | *B. thetaiotaomicron* | UNKNOWN | MGGFFGTVSQVSCVTDLFYGTDYNSHLGTKRGGLATYSEERGFIRSIHNLESTYFRTKFEDELDKFKGNAGIGIISDTDAQPIIINSHLGRFAIVTVAKITNIKELEDELLSQNMHFAELSSSNTNQTELIALLIIQGKTFVEGIENVFKHIKGSCSMLLLTEDGSIIAARDQWGRTPVVIGKKEGAYAATSESSSFPNLDYEIDRYLGPGEIVRLYSDHVVQLRKPGEGMQICSFLWVYYGFPTSCYEGKNVEEVRFTSGLKMGQMDKSEVDCACGIPDSGVGMALGYAEGKGVPYHRAISKYTPTWPRSFTPSNQEMRSLVAKMKLIPNRAMLQGKRLLFCDDSIVRGTQLRDNVKILYDYGAKEVHMRIACPPLIYACPFVGFSASKNALELITRRIIKELEGDEDKNLEKYATTGSPEYEKMVSIIAERFGLTSLKFNTLETLIEAIGLPKCKVCTHCFDGSSHF | amidophosphoribosyltransferase | N | N | 0 | 0 | Cyto. | Ic | 0 | 931 | 100 | 94.03 | 47 | Core |
| C44 | C44.UNW | UP | *Emericella nidulans* | UNKNOWN | MATAYHNLSEYDFNSVPNAEGMKFGIVVSEWNFNITGALLKGAVDTLKKHGAKDENILVKTVPGSFELTFGANQMMENCDVDAIIAIGCVIKGDTPHFDYVCMGATQGITELNATGDIPVIYGLITTNTMEQAEDRAGGKLGNKGDECAITAIKMIDFVWSLNK | 6,7-dimethyl-8-ribityllumazine synthase | N | N | 0 | 0 | Cyto. | Ic | 1.7E-21 | 90,9 | 78 | 32.738 | 47 | Core |
| C44 | C44.UPW | UP | *B. thetaiotaomicron* | UNKNOWN | MEQLKHECGVAMIRLLKPLEYYEKKYGTWMYGLNKLYLLMEKQHNRGQEGAGLACVKLEANPGEEYMFRERALGSGAITEIFENVQNNFKDLTSEQLHDAEFAKRTLPFAGETYMGHLRYSTTGKSGISYVHPFLRRNNWRAKNLALCGNFNMTNVDEIFARITAIGQHPRKYADTYIMLEQVGHRLDREVERVFNLAEAEGLTGMGITNYIEEHIDLANVLRTSSREWDGGYVICGLTGSGESFAIRDPWGIRPAFWYQDEEIAVLASERPVIQTAFNVPVEDIKELQPGQALLISKEGKLRTSQINKPREKQACSFERIYFSRGSDVDIYKERKRLGEKLVPNILKAINNDLDHTVFSFIPNTAEVAFYGMLQGLDDYLNEEKVQQIAALGHNPNMEELEVILSRRIRSEKVAIKDIKLRTFIAEGNSRNDLAAHVYDITYGSLVSGVDNLVIIDDSIVRGTTLKQSIIGILDRLGPKKIVIVSSSPQVRYPDYYGIDMAKMSEFIAFRAAVELLKERDMKDIIASAYRKSKDQIGLPKEQMVNYVKEIYAPFTDEEISAKMVELLTPKGTKAKVEIVYQPLEGLHEACPNHRGDWYFSGNYPTPGGVKMVNQAFINYIEQLYQF | amidophosphoribosyltransferase | N | N | 0 | 0 | Cyto. | Ic | 0 | 1258 | 100 | 95.534 | 47 | Core |
| C44 | C44.UPW | UP | *Eremothecium cymbalariae* | UNKNOWN | MGDPRAFLNIPRQEAGYRPVNERITDYSQVEQTLNTNSRKLQASRCMDCGVPFCHWACPIGNKQPEWQDALFKGKWREAYEILSSTCDFPEFTGRICPALCEKSCVLKLSCDQPVTIRENEAAIVEAAFREGYIQIQTPERNGKKVAVIGAGPAGLVVANQLNLKGYSVTLFDKNEAPGGLLRFGIPNFKLDKNVIDRRMNILTAEGIKFEMGVEIDVNHLPEGFDAYCICTGTPAARDLSIPGRDLKGIYFALEMLAQQNRILEGQTFSKDKLVNAKGKKVLVIGGGDTGSDCIGTSVRQGATSVTQIEIMPQPPVGHNPSTPWPQWPVVLKTTSSHEEGCIRRWCLTSNQFLGKNGNVTGVEVEEVEWIPATDGGRPTLKPTGKKEVIEADMVLLAMGFLKPEQPNFADNVFLAGDAATGASLVVRAMAGGRKTAAEIDTYLTTKN | dihydropyrimidine dehydrogenase subunit A | N | N | 0 | 0 | Cyto. | Ic | 1.2E-108 | 353 | 98 | 41.35 | 47 | Core |
| C56 | C56.UNW | Non-peptidase homologues | *B. thetaiotaomicron* | ARCHEA – The only member of the family for which peptidase activity has been reported is the PfpI endopeptidase (C56.001). This shows a preference for bulky, hydrophobic P1 residues, and Suc-Ala-Ala-Phe-NHMec serves is a test substrate (Halio et al., 1997). The enzyme acts only on peptides of less than 20 amino acids (Hicks et al., 2004). It should be noted that the specificity for hydrophobic P1 residues is similar to that of peptidases in family S8, which are also expressed by Pyrococcus furiosus, so care is needed in distinguishing the two activities. | MGTVYAFFADGFEEIEAFTAIDTLRRGGLNVEIVSVTPDEIVVGAHDVSVLCDINFDNCDFFDAELLLLPGGMPGAATLDKHEGLRRLILDFAAKNKPIAAICAAPMVLGKLGLLKGKKATCYPSFEQYLEGAECIDAPVVRDGNIITGMGPGAAMEFALTIVDLLVGKEKVDELVEAMCVKR | thiazole biosynthesis protein ThiJ | N | N | 0 | 0 | Cyto. | Ic | 1.33E-123 | 346 | 100 | 92.35 | 47 | Core |
| C56 | C56.UNW | Non-peptidase homologues | *B. thetaiotaomicron* |  | MKEVIFVILEGFADWEGAYIATCLNQGVKPGNPISYKVKTLSITKEPVSSIGGFKVLPDYGLNDMPENYAGLVLIGGMNWFSPLGELIVPLVEKAVKENKLVAGICNASVFLGMHGFLNEVKHTSNTLDYLKQYAGDKYTGDSNYINKQAVRDGNIVTANGTGQLEFCKEILYALEADTTDAIEESYLFYKNGFCPET | Hyp. prot. | N | N | 0 | 0 | Unknown | Ic | 3.06E-122 | 344 | 99 | 79.695 | 47 | Core |
| C56 | C56.UNW | Non-peptidase homologues | *Mesorhizobium loti* |  | METKELNKQEYLLRINKVTDYIHNNIDQPLPLQKMAGIACFSPFHFHRVFTILTGETPTDYIKRTRIEKAAQLLKRNKELSATEIARLCGFSSLSLLSRNFRQYFNMTIREFRSHQ | AraC family transcriptional regulator | N | N | 0 | 0 | Cyto. | Ic | 0.000000000000000562 | 72,8 | 84 | 37.755 | 47 | Core |
| C56 | C56.972 | phosphoribosylformylglycinamidine synthase | *B. fragilis* |  | MILFFRTPSKSVIAVESNHQLTPDESNKLCWLFGEAVTESEENLKGCFVGPRREMITPWSTNAVEITQNMGLEGITRIEEYFPVKDENADHDPMLQRMYKGLDQNVFTTNRQPEPIIYIEDLEDYNEKEGLALSKEEMDYLKKVENDLGRKLTDSEVFGFAQINSEHCRHKIFGGTFIIDGVEQESSLFQMIKKTTQENPNKIISAYKDNVAFAEGPVVEQFAPADHSKPDFFQIKDIKSVISLKAETHNFPTTVEPFNGASTGTGGEIRDRMGGGKGSWPIAGTAVYMTSYPRTDEGREWEEILPVRKWLYQTPEQILIKASNGASDFGNKFGQPLICGSVLTFEHTENNEVYGYDKVIMLAGGVGYGTQRDCLKGAPEAGNKVVVIGGDNYRIGLGGGSVSSVDTGRYSSGIELNAVQRANAEMQKRANNVVRALCEEDVNPVVSIHDHGSAGHVNCLSELVEECGGLIDMSKLPIGDKTLSAKEIIANESQERMGLLIKEEAIEHVRKIAERERAPMYVVGETTGDHRFAFQQADGVRPFDLAVEQMFGSSPKTYMIDKTVERHYEMPKYELSQLHEYLTNVLQLEAVACKDWLTNKVDRSVTGKVARQQCQGELQLPLSDCGVVALDYRGEKGIATSIGHAPQAALADPAAGSILSVSEALTNLVWAPMAEGMDSISLSANWMWPCRSQEGEDARLYTAVKALSDFCCALQINVPTGKDSLSMTQKYPNGEKVISPGTVIVSAGGEVSDVKKVVSPVLVNNEKTTLYHIDFSFDELKLGGSAFAQSLGKVGDDVPCVQDAEYFRDAFLAVQELVNKGLILAGHDISAGGLITTLLEMCFANVEGGMEISLDKMKEQDIVKILFAENPGIVIQVSDKHKEEVKKILEDAGVGYLKLGKPTDERHILVSKGDATYQFGIDYMRDVWYSSSYLLDRKQSMNGCAKKRFENYKMQPVEFAFMPEFKGKLSQYGITPDRRTPSGVRAAIIREKGTNGEREMAYSLYLAGFDVKDVTMTDLISGRETLEDVNMIVYCGGFSNSDVLGSAKGWAGGFLFNEKAKEALDKFYAREDTLSLGICNGCQLMMELGLINPEHKKKGKMLHNDSHKFESTFVGLTIPTNRSVMFGSLSGSKLGIWVAHGEGKFSLPYDEDKYNVVAKYSYDEYPGNPNGSDYSIAGLASADGRHLAMMPHLERAIFPWQNGCYPADRKNSDQVTPWIEAFVNARKWIEEKVR | phosphoribosylformylglycinamidine synthase | N | N | 0 | 0 | Cyto. | Ic | 0 | 2455 | 99 | 94.562 | 47 | Core |
| C59 | C59.UPW | Non-peptidase homologues | *B. thetaiotaomicron* | UNKNOWN | MKKKQTGVILMLAALSLMSIQPVEACTRAVYLGPEQLVITGRTMDWKEDIMSNIYVFPRGIQRVGHNKEKTVNWTSKYGSVIATGYDIGTCDGMNEKGLVASLLFLPESIYSLPGDIRPAMGISIWTQYVLDNFATVREAVDELKKETFRIDAPRMPNGGPESTLHMAITDETGNTAVLEYLDGKLSIHEGKEYQVMTNSPRYEYQLAINDYWKEVGGLQMLPGTNRSSDRFVRASFYIHAIPQTADAKIAVPSVLSVMRNVSVPFGINTPEKPHISSTRWRSVSDQKNKVYYFESTLTPNLFWLDLKKIDFSPKAGIKKLSLTKGEIYAGDAVKDLKDSESFTFLFETPLM | choloylglycine hydrolase | Y (SP) | Y | 0 | 0 | Unknown | Exp. | 0 | 699 | 100 | 93.75 | 47 | Core |
| C69 | C69.UPW | UP | *B. thetaiotaomicron* | UNKNOWN | MKKRLIFCAALFLAAVADTFACTNLIVGKNASTDGSTIVSYSADSYGLFGELYHYPAATYPKGTMLKVYEWDTGKYLGEIEQARQTYNVVGNMNEYQVTIGETTFGGRPELADSTGIIDYGSLIYIGLQRSRTAREAIKVMTDLVQQYGYYSEGESFTIADPNEIWIMEMIGKGPGIRGAVWVAVRVPDDCISAHANQSRIHQFDMNDKENCIYSPDVVSFAREKGYFSGVNKDFSFANAYAPLDFGARRFCEARVWSYFNKFTDNGKDYLPYIEGKTNTPMPLFVKPKHKLSVQDVKDMMRDHYEGTPLDISNDFGAGLYKTPYRLSPLNFKVGDREYFNERPISTQQSGFVFVAQMRANKPDPIGGVLWFGVDDANMAVFTPVYCCATKAPVCYTRVDGADYITFSWNSAFWIFNWVSNMVYPRYSLMIDDVRATQKELETTFNNAQEGIEEMAARLLAKDKSAAVEFLTNYTNMTAQSTFDTWKQLGTFLIVKYNDGVVKRVKDGKFERNSIGQPAGVIRPGYPKEFLEEYVKQTGKRYEVPE | peptidase C69 | Y (SP) | Y | 0 | 0 | Unknown | Exp. | 0 | 1071 | 99 | 93.015 | 12 | Shell |
| M49 | M49.UPW | UP | *Porphyromonas gingivalis* | UNKNOWN | MDIKEQLKDIKTQLRLSMNGAVSQSMREKGLLYKLNFGVELPRIKMIAEGYEKNHDLAQALWKENIRECKIMAAMLQPIDTFYPEIADIWVEDIQNIEIAELTCMNLFQHLPYAPAKSLHWIADEQEYVQTCGFLTAARLLMKKGDMTERASGELLDQAICAVHSESYHVRNAALLVIRKYMQHNEEHAFQVCRLVEGMADSEVEAEQILYNMVKEEAADL | peptidase | N | N | 0 | 0 | Cyto. | Ic | 1.25E-22 | 96,3 | 90 | 32.02 | 4 | Cloud |
| M97 | M97.UPW | UP | *B. cellulosilyticus* | UNKNOWN | MSGETHSTSATIPATENQSWFKKRKKKPEQEEKTKSDYEKLVEGSKITKGMFAVHQKKNDYYFEIPTSLLGRDLLIVNKLQRVPAELNDAGVNRGVNYENQMVSMEWDKATGKLMFRQQRPLPLAPQTDAIFRSVKDNFISPLIAAFKIEAINQDSTALVIKVNDIYDGTETSINNVFTNINLGTSTIKNLSRILSIKSFPNNVVATSELTTKVTEGTTSVYVTVEVSSSILLLPEKPMTGRFDNQKVGYFTNPLLSFSDAQQGTDKKQYITRWRMEPKPEDREAYLKGQTVEPIKPIVFYIDNSTPYQWRSYIKKGIEDWQTAFEKAGFKNAIIAKEITDSMHVDMDDVNYSVLTYAASEKKNAMGPSLLDPRSGEILEADIMWWHNVLSMVREWITVQTGTVCPEARSVQLPDSLMGDAIRFVACHEVGHSLGLRHNMMGSWAFPTDSLRSAAFTSRMNSTASSIMDYARFNYIAQPGDGVTVLSPHIGPYDMFAIEYGYRWYGKNTPEEEKDILFDFLSKHTDRLYKYSEAQDVRDAVDPRAQNEDLGDDPVRSSQLGIANLKRIVPQILQWTTTGEKGQTYEEASRLYYAVINQWNNYLYHVLANIGGIYIENTIVGDGVKTYTFVEKEKQQAALKFLMDEVLTYPKWLFDTEVGQYTYLLRNTPIGKQENAPTQILKNAQAYILWDLLGNTRLMRMIENESVNGKKAFTVVELMDGLHKNIFGITERGGIPNVMERSLQKNFLDALLTAAAEPEAVKINKKIANEHFLLDHTTPFCSCYAAEQRALRQEDRMGAPRVLNFYGSQLNRISDAISVKRGELLRIKKLLQNRLGTSDTATRYHYEDMILRINTALGIK | peptidase M10A/M12B | N | N | 0 | 0 | Unknown | Ic | 0 | 1459 | 98 | 81.324 | 12 | Shell |
| M97 | M97.UPW | UP | *B. salyersiae* | UNKNOWN | MRKLTTFLLLLAMLIPTAGAQSTDIFKKKKKKNSKTEAVDKAKADSIAKAKKSPFQPYASVITGKAKTMNGFFKVHCIEGKYFFEIPDSLFGRDILIVNRIVKAPVDKQKRKAGYPGDHISDEVIRFELGRDNKLFIRQISYLEHSTDTLGMYQAVLNSNVQPIVATFPLKTMRKDSLTKNYVIEMTDFIRRDNDMFSFSNYAKDNIGATSMISDASYIDTLKAFPQNIEIRTVRTFQRKPPMGSALEKMIAQYYNSTGPMTYELNSSMLLLPKEPMKPRLYDPRVGYFAVGYKDFDGNPHGMKYKANITRWRLEPKDEDKERYLRGELVEPKKPIVIYIDPATPKKWVPYLIQGVNDWQKAFEKAGFKNAIIGKEAPTDDPTWSLEDARHSAIVYKPSDIPNASGPHVHDPRSGEILETHINWYHNVMLLLYNWYIVQAGAIDPGARKPQFDDELMGELIRFVSSHEVGHTLGLRHNFGSSATVPVEKLRDKAWVEANGHTPSIMDYARFNYIAQPEDSISRAGIFPRIGIYDDWSIEWGYRWMPEFKTAEDEIPHMNKWIISKLKEDKRYTFGTESDRDDPRNQNEDLGDDAMLAGTYGIKNLKRIMPEIIKWTYEPNEGYEKARTLYSNVAGQFSLYMGHVATNVAGIYSTPISVEQTDVKAVEFVPKEIQKKAMAFLNKELFTMPTWLMDSQLLEKAQVNTSSFIFSVQSGILKGLLSSRTLDKMTTNELMNGTKAYTSAEMFQDLRKSIWNDLRGGKRPDLNQRALQKVYVNSLTAMLEKPKTNANQYMPDSLSEASAIARGQLTDLRRDLANAASASGGIYRSHYLNLKALIDTAFEAK | Hyp. prot. | Y (SP) | Y | 0 | 0 | Unknown | Exp. | 1.19E-166 | 506 | 92 | 39.627 | 1 | Cloud |
| A02A | A02.007 | feline immunodeficiency virus retropepsin | *NaN* | Retropepsin is autolytically released from the gag-pol polyprotein and the peptidase has to dimerize to be active. How this process is initiated is not known, but it may follow release of retropepsin monomers from the viral polyprotein by host peptidases. Similar processes occur in other retroid viruses, including important pathogens such as human T-cell leukemia virus, equine infectious anemia virus, mouse mammary tumor virus and Rous avian sarcoma virus. Homologous proteins are encoded in mammalian genomes, including the product of the human vprt gene and the mouse intracisternal A-particle, but the functions and origins of these are unknown. | MNIQVINKSKHSLPAYATELSAGMDIRANLSEPITLEPLQRCLVPTGLYIALPQGFEAQIRPRSGLAIKKGITVLNSPGTIDADYRGEICIILVNLSSETFVIEDGERIAQMVIAKHEQAVWQEVEVLDETERGAGGFGHTGKK | deoxyuridine 5'-triphosphate nucleotidohydrolase | N | N | 0 | 0 | Cyto. | Ic | 0.000000000000000718 | 73,9 | 86 | 37.302 | 47 | Core |
| A01A | A01.UPA | UP | *Magnaporthe grisea* | UNKNOWN | MAKQDLHLTRNIGIMAHIDAGKTTTSERILFYTGLTHKIGEVHDGAATMDWMEQEQERGITITSAATTTRWKYAGDTYKINLIDTPGHVDFTAEVERSLRILDGAVAAYCAVGGVEPQSETVWRQADKYNVPRIAYVNKMDRSGADFFEVVRQMKDVLGANPCPIVVPIGAEESFKGLVDLIKMKAIYWHDETMGADYSVEEIPADLVDEANEWRDKMLEKVAEFDDTLMEKYFDDPSTITEEEVLRALRNATVQMAVVPMLCGSSFKNKGVQTLLDYVCAFLPSPLDTENVIGTNPDTGAEEDRKPSDDEKTSALAFKIATDPYVGRLTFFRVYSGKIEAGSYIYNSRSGKKERVSRLFQMHSNKQNPVEVIGAGDIGAGVGFKDIHTGDTLCDETAPIVLESMDFPEPVIGIAVEPKTQKDMDKLSNGLAKLAEEDPTFTVKTDEQTGQTVISGMGELHLDIIIDRLKREFKVECNQGKPQVNYKEAITKTVDLREVYKKQSGGRGKFADIIVKIGPVDEDFKEGGLQFIDEVKGGNIPKEFIPSVQKGFTTAMKNGVLAGYPLDSLKVTLVDGSFHPVDSDQLSFEICAIQAYKNACSKAGPVLMEPIMKLEVVTPEENMGDVIGDLNKRRGQVEGMESSRSGARIVKAMVPLAEMFGYVTALRTITSGRATSSMVYSHHAQVSTSIAKAVLEEVKGRADLL | elongation factor G | N | N | 0 | 0 | Cyto. | Ic | 3.66E-169 | 520 | 97 | 42.029 | 47 | Core |
| M28F | M28.UPF | UP | *B. thetaiotaomicron* | Glutamate carboxypeptidase II (M28.010) degrades the neuropeptide Ac-AspGlu in the brain and also converts folylpoly-gamma-glutamate to pteroylglutamate in the intestine. IAP aminopeptidase is thought to convert one form of bacterial alkaline phosphatase to another by successively releasing N-terminal arginines (Ishino et al., 1987). | MKEKVLLIFFLCNVALFVSAQSHMEKGLQSISRSSTEAIINFLAGDELQGREAGFHGSRVASEYIVSLLQWIGVQPLNESYFQPFEAYRKERQKKGRLEVHPDSIAKLKQEVHQKLSMRNVLGMIPGKNTKEYVIVGAHFDHLGIDPALDGDQIYNGADDNASGVSAVLQIVRAFVASGKQPERNVIFAFWDGEEKGLLGSKYFVQTCPFISRIKGYLNFDMIGRNNKPQQPEHVVYFYTAAHPVFGDWLKEDIKKYGLRLSPDYRAWDNPVGGSDNGSFAKAGIPIIWYHTDGHPDYHQPSDHADRLNWDKIVEITKASFLNMWKMSNEREF | aminopeptidase | Y (SP) | Y | 0 | 0 | Extracell. | Exp. | 0 | 607 | 99 | 86.667 | 1 | Cloud |
| S01B | S01.UPB | UP | *Rhodopirellula baltica* | The peptidases of family S1 play many roles. These include intestinal digestion (trypsin and chymotrypsin), IgA-mediated immune response (tryptase and chymase from mast cells and the granzymes found in cytotoxic cells), and the role of the snake and easter peptidases in the dorsal-ventral signalling pathway of drosophila. | MEKFEDLIQSPMPVLVDFFAEWCGPCKAMKPVLEELKLVVGDKARIVKIDVDQHEDLATKYRIQAVPTFILFKNGEAVWRHSGVIHSSELQGVIERHYT | thiol reductase thioredoxin | N | N | 0 | 0 | Cyto. | Ic | 0.0000000000609 | 58,2 | 84 | 36.145 | 2 | Cloud |
| C26 | C26.A25 | trp1 | *B. thetaiotaomicron* | UNKNOWN | MKILLLDNYDSFTYNLLHAVKELGATDIEVVRNDQIGLDNVERFDKIILSPGPGIPEEAGLLLPIIKKYAATKSILGVCLGHQAIGEAFGARLENLKEVYHGVQTPVSILRQDVLFEGLGKEIPVGRYHSWVVSREDFPDCLEITAESREGQIMALRHRTYDVHGIQFHPESVLTPQGKEIIKNFLND | glutamine amidotransferase | N | N | 0 | 0 | Cyto. | Ic | 1.56E-130 | 364 | 100 | 93.085 | 47 | Core |
| C26 | C26.A25 | trp1 | *Schizosaccharomyces pombe* | UNKNOWN | MKDILSEIIANKRFEVDLQKQAISIEQLQEGISEMPSSRSMKQALISSSPGIIAEFKRRSPSKGWIKQEARPEEIAPAYAAAGASALSILTDEKFFGGNLKDIRAARPLVDIPILRKDFIIDEYQLYQAKIVGADAVLLIAAALKPEKCNELVKKAHDLGLEVLLEIHSSEELTYINEKIDMVGINNRNLGTFFTDVENSFRLAGQLPQDAVLVSESGISDPQTVKRLQTAGFRGFLIGETFMRTTQPGNTLGNFLQAIQ | indole-3-glycerol-phosphate synthase | N | N | 0 | 0 | Cyto. | Ic | 6.66E-46 | 164 | 95 | 38.911 | 47 | Core |
| C26 | C26.955 | aminodeoxychorismate synthase, subunit II | *Spirochaeta africana* | Gamma-glutamyl hydrolase is a lysosomal enzyme probably involved in the turnover of folyl poly-gamma-glutamates. | MKQILYKLFEHQYLGRDEARTILQNIAQGKYNDVQVASLITVFLMRNISVEELCGFRDALLEMRVPVDLSEFAPIDIVGTGGDGKNTFNISTASCFTVAGAGFPVVKHGNYGATSVSGASNVMEQHGVKFTDDIDQLRRSMEQCNIAYLHAPLFNPALKAVAPVRKGLAVRTFFNMLGPLVNPVLPAYQLLGVYNLPLLRLYTYTYQESKTKFAVVHSLDGYDEISLTNEFKVATSDNEKIYTPESLGFSRYRDIDLDGGQTPKEAAKIFDNIMNNTATEAQKNVVVVNSAFAIHVICPKKTIEECITLAKESLESGRALNTLKKFIELNN | anthranilate phosphoribosyltransferase | N | N | 0 | 0 | Cyto. | Ic | 3.35E-52 | 181 | 99 | 32.44 | 47 | Core |
| C26 | C26.956 | carbamyl phosphate synthetase | *Candida glabrata* |  | MENRSLVTIAEHSKEKILYMLEMAKQFEMNPNRRLLQGKVVATLFFEPSTRTRLSFETAANRLGARVIGFSDPKATSSSKGETLKDTIMMVSNYADIIVMRHYLEGAARYASEVAPVPIVNAGDGANQHPSQTMLDLYSIYKTQGTLENLNIFLVGDLKYGRTVHSLLMAMRHFNPTFHFIAPDELKMPEEYKLYCKTHQIKYVEHTDFSEEIIADADILYMTRVQRERFTDLMEYERVKNVYILRNKMLENTRPNLRILHPLPRVNEIAYDVDDNPKAYYFQQAQNGLYAREAILCDVLGITLEDVKNDILL | aspartate carbamoyltransferase | N | N | 0 | 0 | Cyto. | Ic | 4.15E-75 | 251 | 96 | 40.523 | 47 | Core |
| U32 | U32.001 | collagenase | *B. thetaiotaomicron* | Peptidase family U32 contains endopeptidases from bacteria. | MSLSLKDFEIMAPVGSRESLAAAIQAGADSIYFGIENLNMRARSANTFTIDDLREIARTCDEHGMKSYLTVNTIIYDKDIPLMHTIVDAAKEAGISAVIAADVAVMDYARRIGQEVHLSTQLNISNAEALKFYARFADVVVLARELNLEQVAEIYRQIQEEHICGPSGEQLRIEMFCHGALCMAVSGKCYLSLHEMNHSANRGACMQVCRRSYTVRDKETDVELDIDNEYIMSPKDLKTIHFMNKMLDAGVRVFKIEGRARGPEYVRTVVECYKEAIKAYLEDTFTDEKIAAWDERLKTVFNRGFWDGYYLGQRLGEWTRNYGSAATERKIYVGKGIKYFSNIGVSEFLVEAAEVSVGDKLLITGPTTGAVFMTLDEARVDLEPVQTVKKGQHFSMKSDKIRPSDKLYKLVSTEELKKFKGLDIEQKRG | collagenase | N | N | 0 | 0 | Cyto. | Ic | 0 | 853 | 99 | 95.082 | 1 | Cloud |
| U32 | U32.003 | collagenase | *B. thetaiotaomicron* |  | MIKQRKIELLAPAKNLECGIEAINHGADAVYIGAPKFGARAAAVNSLEDIEALVKHAHLYNARIYVTVNTILKEEELQETEEMIHALYRIGVDALIVQDMGITQLNLPPIPLHASTQMDNRTPEKVKFLKEAGFRQVVLARELSLREIKKIHDTCPEVPLEVFVHGALCVSYSGQCYVSQACFGRSANRGECAQFCRLPFSLVDSDGKVMVKDKHLLSLKDLNQSDELEQLLDAGASSFKIEGRLKDVSYVKNVTATYRQKLDAIFARRQEYVRASSGTCRFDFRPQLDKSFSRGFTHYFLHGRDKEIFSFDTPKSLGEEMGTMKEARGNYLTVAGLKSFNNGDGVCYIDEQGRLQGFRINRVDGNKLYPQEMPQIKPRTRLYRNFDQEFERILSRKSAERKLSVSILLADNNFGFSLTLTDEDDNSVTLSLPREKELARTPQTDHLRTQLAKLGNTPFEAEKIEISFADNWFLPSSVLADFRRQAVEKLIAARRINYHQERAVWKPTNHAFPQTALTYLGNVMNTRAASFYQEHGVQQIAGAYEKERVEDAVLMFCKHCLRYSMGWCPIHQRVRSPYREPYFLVSNDGKRFRLEFDCKNCQMKVKAAQ | protease | N | N | 0 | 0 | Cyto. | Ic | 0 | 1174 | 100 | 92.611 | 47 | Core |
| C26 | C26.964 | CTP synthetase | *Capnocytophaga ochracea* | Non-peptidase homologue | MGETKYIFVTGGVASSLGKGIISSSIGKLLQARGYNVTIQKFDPYINIDPGTLNPYEHGECYVTVDGHEADLDLGHYERFLGIQTTKANNITTGRIYKSVIDKERRGDYLGKTIQVIPHITDEIKRNVKLLGNKYKFDFVITEIGGTVGDIESLPYLESIRQLKWELGKNALCVHLTYVPYLAAAGELKTKPTQHSVKELQSVGIQPDILVLRAEHPLSDGLRKKVAQFCNVDDKAVVQSIDAETIYEVPILMQAQGLDSTILEKMGLPVGETPGLGPWRKFLERRHAAETKEPINIALVGKYDLQDAYKSIREALSQAGTYNDRKVEVHFVNSEKLTDENVGEALKGMAGVMIGPGFGQRGIDGKFVAIKYTRTHDIPTFGICLGMQCIAIEFARNVLGFADANSREMDEKTPHNVIDIMEEQKAITNMGGTMRLGAYECVLQKGSKVFEAYGQEHIQERHRHRYEFNNDYKAQYEAAGMKCTGINPESDLVEIVEIPALKWYVGTQFHPEYSSTVLNPHPLFVAFVKAAIENEK | CTP synthetase | N | N | 0 | 0 | Cyto. | Ic | 0 | 718 | 99 | 64.06 | 47 | Core |
| T05 | T05.UPW | UP | *Anaeromyxobacter dehalogenans* | YEAST – Only the precursor of ornithine acetyltransferase has peptidase activity. The mature form of the enzyme released by the autoproteolytic cleavage of the precursor catalyses the reversible interchange of an acetyl group between N-acetylornithine and glutamate (Liu et al., 1995) | MREKLTVIKVGGKIVEEEATLRQLLNDFAAIEGHKVLVHGGGRSATKIAAQLGIESKMVNGRRITDAETLRVVTMVYGGLVNKNIVAGLQAYGVNALGLTGADMNVIRSVKRPVKEVDYGFVGDVEKVDASLLSDLIYKGVVPVMAPLTHDGQGNMLNTNADTIAGETAKALSGLFDVTLVYCFEKKGVLRDENDDDSVIPQITRAEFEQYVADGVIQGGMIPKLENSFEAINAGVSEVIITLASAINGNGGTRIKK | acetylglutamate kinase | N | N | 0 | 0 | Cyto. | Ic | 6.34E-24 | 101 | 92 | 33.333 | 47 | Core |
| M16B | M16.A05 | PqqL protein | *B. fragilis* | MPP removes an N-terminal targeting signal from mitochondrial protein molecules that have been synthesized in the cytoplasm as they are imported into the mitochondrion. There is much interest in the possibility that insulysin (M16.002) may be a physiologically significant alpha-secretase, usefully degrading the amyloidogenic Alzheimer"s beta-peptide (Edland, 2004). | MGFNEFLSSIFGNKSTRDMKEIKPWVEKIKAAYPEIEKLDNDALRAKTEELKKYIHESATAERAKVEELKASIESLELEDREEVFAQIDKIEKEILDKYEKALDEVLPVAFSIVKATAKRFTENEEIVVAATDFDRQLAATKDFVRIEGDKAIYQNHWIAGGNDTVWNMVHYDVQLFGGVVLHKGKIAEMATGEGKTLVATLPVFLNALTGNGVHVVTVNDYLAKRDSEWMGPLYMFHGLSVDCIDRHQPNSDARRQAYLADITFGTNNEFGFDYLRDNMAISPKDLVQRQHNYAIVDEVDSVLIDDARTPLIISGPVPKGDDQLFEQLRPLVERLVEAQKVLATKYLSEAKKLIASNDKKEVEEGFLALYRSHKCLPKNKALIKFLSEQGIKAGMLKTEEIYMEQNNKRMHEVTEPLYFVIDEKLNSVDLTDKGIDLITGNSEDPTLFVLPDIAAELSELENMNLTNEQLLEKKDELLTNYAIKSERVHTINQLLKAYTMFEKDDEYVVIDGQVKIVDEQTGRIMEGRRYSDGLHQAIEAKEGVKVEAATQTFATITLQNYFRMYHKLSGMTGTAETEAGELWDIYKLDVVVIPTNRPIARKDMNDRVYKTKREKYKAVIEEIEQLVNAGRPVLVGTTSVEISEMLSKMLTMRKIEHKVLNAKLHQKEADIVATAGLSGTVTIATNMAGRGTDIKLSPEVKAAGGLAIIGTERHESRRVDRQLRGRAGRQGDPGSSVFFVSLEDDLMRLFSSDRIASVMDRLGFQEGEMIEHKMISNSIERAQKKVEENNFGIRKRLLEYDDVMNKQRTVVYTKRRHALMGERIGMDIVNMIWDRCANAIENNDYEGCQMELLQTLAMETPFTEEEFRNEKKEKLAEKTFGIAMENFKRKTERLAQIANPVIKQVYENQGHMYENILIPITDGKRMYNISCNLKAAYESESKEVVKAFEKSILLHVIDEAWKENLRELDELKHSVQNASYEQKDPLLIYKLESVTLFDSMVNKINNQTVSILMRGQIPVQEAPDEQAARRVEVRQAAPEQRQDMSKYRENKQDLSDPNQQAAASQDTREQQRREPIRAEKTVGRNDPCPCGSGKKYKNCHGKNV | preprotein translocase subunit SecA | N | N | 0 | 0 | Cyto. | Ic | 0 | 919 | * | 46.23 | 47 | Core |
| M16B | M16.A05 | PqqL protein | B. thetaiotaomicron |  | MKHLLRGLFIAVLIICCNFQSVFAQPMQQMPVDKNVRIGKLDNGLTYYIRHNALPEKRVEFYIAQKVGSILEEPQQRGLAHFLEHMAFNGTKHFPGDETGLGIIPWCETKGIKFGTNLNAYTSVDQTVYNISNVPTENQNVVDSCLLILHDWSSAINLADKEIDKERGVIREEWRSRNSGMLRIMTDAQATMYPDSKYADCMPIGSIDVINNFPYQDIRDYYAKWYRPDLQGIVIVGDINAEEMEAKLKEVFKDVKAPVNPAERIYYPVADNQEPLIYIGTDKEVANPSINIFFKQDATPDSLKNTISYYATQYVLNMAINMLNSRLNELRQTANPPFTGAGAGYGEYFLAKTKEAFSLTANSKIDGVDLAMKTILEEAERARRFGFTETEYERARANYMQAMESAYNEREKTKSGNYVDEYVNNFLDKEPIPGIEFEYMLVQQMAPNIPVTAVNELMKQLVTDNNQVVLLAGPQKEGLKYPTKEEIAALLKQMSSFDLKPYEDKVSNEPLISEDIKGGKIVSEKADDVYGSTKLVLSNGVTVYVKPTDFKADQIMMKGVSLGGTSVFPNDEIINISQLNGVALVGGIGNFSKVDLSKALAGKRASVGAGIGNTTETISGSCSPKDFETMMQLTYLTFTSPRKDNEAFESYKNRLKAQLQNSDANPMTAFSDTVTRALYGDHPRAIKLKESMVDQIDYDRILEMYKDRYKDASDFTFYLVGNVNLEQMKPMIAKYLGALPSINRKETFKDNKMYIRKGEYKNEFAKKQETPMATIMFLYSGTCKYDLRSNTLLSFLDQALDMVYTAEIREKEGGTYGVSCNGNLSKYPKEELVLQIVFQTDPAKKDKLSAIVVEQLEKMAKEGPSAEHMQKIKEYMLKKYKDAQKENGYWLNNLDEYFYTGIDNTKDYEKLVNSITAKEVQDFLAKLLKQNNEIQVVMTMPEENK | peptidase M16 | Y (SP) | Y | 0 | 0 | Unknown | Exp. | 0 | 1687 | 99 | 83.245 | 1 | Cloud |
| M16B | M16.UNB | Non-peptidase homologues | *B. thetaiotaomicron* |  | MNRKIQPEIQTLKDFRILPPVRTTLPNGIPLTVINAGEQDVVRMDILFGGGRWQQSQKLQALFTNRMLREGTKKYTAATIAGKLDYYGSWLELSSSSDYAYITVYSLNKYVAETLEVVESMIKEPLFPEKELHTILDTNIQQYLVNTSKVDFLAHRSLLQSLYGEQHPCGKIVVEEDYHAITPEVLRNFYERYYHSGNCSIFLSGKVTEDIIRRVKDAFGSPFGQYQLQTSKLNFPYIAVPEKRIFTEREDAMQSAVKMGYTTITREHPDYLKLRVLMTVFGGYFGSRLMSNIREEKGYTYGISAGIMFYPDSGLLIVSTETDNEYVEPLIQEVYHEIDRLHQEVVPVEELSMVRNYMLGEMCRSYESAFSLSDAWIFIATSGLGDDYFSRSLQAVQEVTPAELQDLAQRYLCKEKLKEVIAGKKIS | peptidase M16 | N | N | 0 | 0 | Cyto. | Ic | 0 | 755 | 100 | 86.651 | 13 | Shell |
| M16B | M16.UPB | UP | *B. thetaiotaomicron* |  | MHCNEYTLPNGLRIIHEPTLSKVAYCGFAIDAGTRDEAANEQGMAHFVEHLIFKGTVKRKAWHILNRMENVGGDLNAYTNKEETVVYSAFLTEHLERALELLGDIVFHSTFPQHEIEKETEVIIDEIQSYEDNPSELIFDDFEDMIFRNHPLGRNILGKPELLRSFRTEDVLSFTRRFYQPGNMVFFVQGQYDFKKIIRLAEKYMSDIPAVEIENRRTPPPLYIPEHLTVTKDTHQAHVMIGSRGYNAYDDKRTALYLLNNILGGPGMNSKLNVSLRERRGLVYNVESNLTSYTDTGAFCIYFGTDIEDMDTCLKLTYKELKRMRDVKMTSSQLAAAKKQLIGQIGVASDNFENNALGMAKTFLHYHKYESSELVFKRIEALTAETLLEVANEMFAEEYLSTLIYR | zinc protease | N | N | 0 | 0 | Cyto. | Ic | 0 | 799 | 100 | 94.089 | 1 | Cloud |
| M16B | M16.019 | YMXG peptidase | *B. thetaiotaomicron* |  | MKINRHILDNGLRLVHSRDESTQMVALNILYNVGARDEHPEHTGFAHLFEHLMFGGSVNIPDYDMPLQLAGGENNAWTNNDITNYYLTVPRQNVETGFWLESDRMLSLDFSERSLEVQRGVVMEEFKQRCLNQPYGDVGHLLRPLAYQKHPYQWPTIGKDLSHVANATLEEVKAFFFRFYAPNNAILAVTGNISFEEAVELTEKWFGSVPRREVPVRNLPQEPEQTEERRLTVERNVPLDSLFMAYHMCDHRHPDYYVFDILSDVLSNGRSSRLNQHLVQEKQLFSSIDAYISGSVDAGLFHIAGKPSAGVSLELAEAAVRDELDRLQQELVDGQELEKVKNKFESTQIFGNINYLNVATNLAWFELLGKAEDLEKEVERYRSVTAMQLREVAQSAFRKENGVVLYYKSK | peptidase M16 | N | N | 0 | 0 | Cyto. | Ic | 0 | 795 | 100 | 91.951 | 1 | Cloud |
| M16 | M16.A05 | UP | *B. fragilis* |  | MFKQTKFKVFLFLAVLLGGLPVNMLAQQKASHLEKKVNLPPNTVEGTLANGLHYLILPNEAPIHTTEFRLVMRIGSVQESEEQKGAAHFLEHMSFAGSKHFPGRGMVDYLETLGMKFGRDINAVTGYDRTIFMLTVPMDKTDHKVSSKTLLILKDWLSGITFEEERTKKERGVILEELRGYDLGDDFYALKIGKNHFTERMPLGSSEDIRSIDRKTLIEFYKKWYSPQMATVVVVGNVDPESIEKQIKEMFSSIPRKEVKGYRTYPLTYDPGVELYEIGDDLERSSELELMIPHPCVIGNTIGSIYQKELGSLLIRAISNRLKYQNIRCNVSDAWFLSDKNHFVFAFSGVDKANLLQQVSELSNEMESIQKNGFKQEEVEDAINEHLRRLKVDNSTQLSSKWCDDFVDYVISDDRYIQSDSEMKQLADKIRATDSATLQQLLREWLSYKDQTLLVAYRNNAGKQNSLKKEEVVQAWDEGIKNPLKDFTYVRKDVKEERVVTPACLVESYPFKASDIVSEKKYADLNITEVILKNDLRILLRPTNDESQSIFVTAFGRGGTADLSDKDYPLYEGTGGYMEMGGVACIPYDTLSSFMQQEEISMNIAISNYWHDIMGMSPAAKARELFNLMYEKMCRPELCYEDFDEIRKDEMERFGKESVLEQMMKRASDRMLTNRLDSLMGNTVPRPALTKMDLERLDLDKIADYYCSLYSNPSQMTFVVTGKFDTDSIKELLTATFGRMPKVNTVSYPNKAFKLPKKTYIEEFPNDNDTQTIFDYVFCGNYQPSLKNSLTLKLMRDILQNRLLSVLREGENIVYSPYASLFYNGLPQQVFYFDLSASVDFVNTKKVEELIKQIINELRTSKVSEEELETLKKSFWVTKRKVLSDEASAEWRTNLVNLLKNGESVADFERYEQCLNSISTVDIQKAFKHFTNPDKFVLLYIGKHQKYE | peptidase | Y (SP) | Y | 0 | 0 | Mul. loc. | Exp. | 0,00E+00 | 919 | 96 | 46.23 | 2 | Cloud |
| M10B | M10.UPB | UP | *Gibberella zeae* | The matrixins in family M10 are mostly secreted proteins that function extracellularly. The matrixins in subfamily M10A are synthesized with conventional signal peptides, but the bacterial serralysin in subfamily M10B is synthesized without a signal peptide, and is secreted by a different mechanism. The matrixins play important roles in the degradation of connective tissue matrix proteins and both in normal tissue turnover and in pathological tissue damage. | MDNSFAYQPATERYERMQYKYCGRSGLKLPLISLGLWHNFGSVDDFTMATDMIKYAFDHGITHFDLANNYGPVPGSAETNFGRILRENFQGYRDEMIISSKAGHEMWAGPYGGNSSRKNLMASIDQSLRRTGLEYFDIFYTHRYDGVTPVEETIQALIDIVKRGKALYIGISKYPPEQARIAYEMLAKAGVPCLISQYRYSMFDRAVEAEILPFAAASGSGFIAFSPLAQGLLTDKYLNGIPEHSRAARSSGFLQRSQVTPEKIEAARQLNEIAHRRGQTLAEMALAWVLRDERMTSVIVGASSVNQLADNLKALEHLEFTSEELAEIEQILSRMIL | glyceraldehyde 3-phosphate reductase | N | N | 0 | 0 | Cyto. | Ic | 1.72E-20 | 93,6 | 89 | 30.065 | 47 | Core |
| S01B | S01.UPB | UP | *Rhodopirellula baltica* | The peptidases of family S1 play many roles. These include intestinal digestion (trypsin and chymotrypsin), IgA-mediated immune response (tryptase and chymase from mast cells and the granzymes found in cytotoxic cells), and the role of the snake and easter peptidases in the dorsal-ventral signalling pathway of drosophila. | MKEKKVAREERNQEKLANADWVMAEFYATWCPHCQRMKPVVEEFKKLMEGTIEVVEVDVDQETALTDFYTIEATPTFILLRKGQQLWRQSGELTLERLERAVKGFKS | thiol reductase thioredoxin | N | N | 0 | 0 | Unknown | Ic | 0.000000000149 | 57,4 | 76 | 37.037 | 2 | Cloud |
| S54 | S54.025 | Rhomboid peptidase | *B. thetaiotaomicron* | Rhomboid-1 acts at two different stages during the development of the Drosophila embryo. Early on, it is required to establish position along the dorsoventral axis. Later, it is required to specify the fate of neuronal precursor cells (Bier et al., 1990). It cleaves the transmembrane proteins Spitz, Gurken and Keren within their transmembrane domains to release a soluble TGFalpha-like growth factor. Cleavage occurs in the Golgi, following translocation of the substrates from the endoplasmic reticulum membrane by Star, another transmembrane protein. The growth factors are then able to activate the epidermal growth factor receptor (Urban et al., 2002). Few substrates of mammalian rhomboid homologues have been determined, but rhomboid-like protein 2 (S54.002) has been shown to cleave ephrin B3 (Pascall & Brown, 2004. The AarA protein (S54.004) is thought to activate a factor that regulates gene expression in response to changes in the population size of the bacterium Providencia stuartii (Rather et al., 1999), but can also activate the Spitz protein (Gallio et al., 2002). In Saccharomyces cerevisiae the Pcp1 protein (S54.007) has a completely different function, and is a mitochondrial endopeptidase required for the activation of cytochrome c peroxidase and for the processing of the mitochondrial dynamin-like protein Mgm1 (Esser et al., 2002; Herlan et al., 2003). The peroxidase is nuclearly-encoded and has to be first processed by mAAA (XM41-001) to remove a targeting signal. | MPTVTKNLIIINVLVFFGTLVAQRYGIDLANYLGLHFFLASDFNPAQLITYMFMHGGFSHIFFNMFAVFMFGPILEQTWGPKRFLFYYILCGIGAGLIQEGVQYIQYITELSQHTEVNLIGYGIISMDQYLNMMTTVGASGAVYAILLAFGMLYPNNQLFIFPLPFPIKAKFFVIGYAAIELWAGLANSAGDNVAHFAHLGGMLFGLILILYWRKKSKNNGTYYN | rhomboid family intramembrane serine protease | N | Y | 6 | 5 | Cyto. Membr. | M | 3.16E-141 | 394 | 100 | 86.222 | 5 | Cloud |
| S54 | S54.UPW | UP | *B. thetaiotaomicron* |  | MGHIITDLKETFRRGNIFIQLIYINVSIFIIGTLINVFLQLFQHSIPDIFGIFALPASFIRFFHQPWSIITYMFMHAGLLHILFNMLWLYWFGSLFLHFFSAKHLRGLYILGGILGGLLYMVAYNVFPLFNQEVAVSTLVGASASVLAIVAATAYREPNYRVQLFLFGAIRLKYLALVVIGIDVLSITSSNAGGHIAHLGGALAGLWFATSLSKGADLTAWINWLLDAFISLFQKKTWKHKPKMKVHYGGGTTSREKDYDYNAHKKAQSDEIDRILDKLKKSGYDSLTTEEKKSLFDASKR | rhomboid family intramembrane serine protease | N | N | 6 | 7 | Cyto. Membr. | M | 0 | 503 | 100 | 81.728 | 12 | Shell |
| S54 | S54.029 | rhomboid peptidase 2 | B. thetaiotaomicron |  | MKQDIQRIMLAAAKPLFLIFILYMLKIVEVGMHWDLSHLGIYPMEKRGVFGILTHPLIHSGFSHLLANTLPLFFLSWCLFYFYRGIAGKIFILIWIGAGLLTFIIGKPGWHIGASGLIYGLAFFLFFSGILRKYVPLIAISLLVTFLYGGIIWHMFPYFSPANMSWEGHLSGGIMGTLCALAFVNHGPQRPEPFADEKDDDEEEEENPKEEFSNQDLCNASINNVIFAVNNLTEIANRIIPKNLRII | rhomboid family intramembrane serine protease | N | N | 6 | 6 | Cyto. Membr. | M | 4.9E-121 | 344 | 79 | 91.282 | 2 | Cloud |
| M79 | M79.UPW | UP | *B. thetaiotaomicron* | UNKNOWN | MEAENIVDKKELKGLPIWACILLFIVVFFVFMLLYSALIQGFLSLVLGVEARHPGIVGYILQETGMLLTALTSAVIMLRFERRPFSDLGLSVKGHARGLWYGLLIAVLFYLVGFGLSLLLGEIEVTGFKFEPVNLLGSWVFFLLVALFEEILMRGYILGRLLHTRMNKFLSLFISSALFALLHIFNPEIDFLPMLNLLLAGMLLGASYLYTKNLCFPISLHLFWNWIQGPVLGYEVSGNNFISSMLTLHLPEDNVLNGGAFGFEGSLICTVLMIVLTILIVWWGEKREAISLAVPQSC | protease | N | N | 8 | 8 | Cyto. Membr. | M | 6.37E-155 | 435 | 100 | 76.846 | 1 | Cloud |
| M79 | M79.007 | MroQ peptidase | *B. thetaiotaomicron* | UNKNOWN | MKTAIKLVLIDLVIAQIVAPVLIMIPCTIYLLVTTGNLDKAVLTQTIIIPAQLAGQIMMGIYLWKAGYISTKKETWSLVSAPYLLGSAVAILTSGFVVSSLMSLLDWIPNIMEQSFDILQSGWGGILAIAIVGPVLEELLFRGAITKALLQQYNPTKAILISALLFGVFHINPAQILPAFLIGILLAWTYYKTGSLIPCIFMHILNNSLSVYLSIKYPEAENMDDLISGTPYLIILIGAIVILAGVIPYMQRMTSNK | protease | N | N | 8 | 6 | Cyto. Membr. | M | 1.83E-132 | 375 | 99 | 76.772 | 1 | Cloud |
| M43X | M43.UPW | UP | *B. fragilis* | In vivo pappalysin circulates bound to proMBP, and in the complex the proteolytic activity of pappalysin-1 is reduced more than 100-fold (Overgaard et al., 2000). | MKTRLIFLLPLLWLLIGCEDSEPESKPDSTDPPLIEYHYELPVVFHVLYQNEQQNIKKGRIQEIITDCNKYYQNRLGSNSVDMNLEFVLATENPQGVKLDEPGVHPIQVSNPVQDCEVFMTDKANLKYLWDTDKYINIMLYPFKQDENSEGVILGISHLPYTIKPDYLEGLNQLNGIPSHSSLKYPHCISINNTYINSTPSNESKKIYSSTDIVATIAHELAHYLGLFHTFSESDDEGLNTCMDTDYCDDTPTYDREAYSNWMQSYIESKNGIKNMSLSEFNKLFERKDCVTNLTSTPNNIMDYDISWVNRFTPNQQERIRYVLMHSPLVPGPKVERTTTRTLSGEQELPMRMIK | zinc-dependent metalloproteinase lipoprotein, BF0631 family | Y (Lipo) | Y | 0 | 0 | Mul. loc. | Exp. | 4.88E-86 | 267 | 90 | 43.939 | 1 | Cloud |
| C01A | C01.UPA | UP | *B. thetaiotaomicron* | Family C1 peptidases contribute proteolytic activity to the digestive vacuoles of protozoa and to the lysosomal system of eukaryotic cells, specific examples of lysosomal enzymes including cathepsin B (C01.060), cathepsin L (C01.032), cathepsin S (C01.034), cathepsin K (C01.036), cathepsin H (C01.040) and dipeptidyl-peptidase I (C01.070). | MKKLLISVAVFAFAIASFAQAPGYEFTTVVSHKATPVKDQSSTGTCWCFATASFIESELLRMGKGEYDLSEMFIVRQKYMNQMEDNYLRRGKGNIGEGSLAHTFKNAYKQVGIVPEEAYSGLIDGNKEHNHGALSRYFKALVDANIASKKRTPQYYALINNLFDTYLGKLPEKFTYKGKEYTPQSFTESLGLNMDDYIELTSFTHKPYYEMFSPEVPDNWENQPMYNLPLDELIETIDYALNKGYTVCWDGDVSEQGFSFKNGVAINPQVEDVKDYSTTDRARFESMPKYQRLDEVFKFEHPYPEIAVTPEIRQEGYEKFVTTDDHLMHITGIAKDQNGTKYYITKNSWGAESNKSGGYLNMSESYVRAKTICVMVHKDSLPKELKQKLGIR | aminopeptidase | Y (SP) | Y | 0 | 0 | Mul. loc. | Exp. | 2.83E-109 | 327 | 100 | 43.108 | 3 | Cloud |
| C01B | C01.UPB | UP | *B. thetaiotaomicron* |  | MNKRILPVFVFCALCYSAQAQDAKGGISDAMMQQIKQSYQNTSADKAIRNAIGSNDIRKLALNQDNMKGMDTHFSVKVNSKGITNQKSSGRCWLFTGLNVMRAKAIDKYHLGSFEFSQTYPFFFDQLEKANLFLQGIIDTSDKPMNDKMVEWLFRNPLSDGGTFTGVADIVSKYGLVPKDVMPETNSSENTSRMAGLITLKLREQGIQLRDMAAKGAKPAALEKTKTEMLGTIYRLLVLNLGVPPTEFTWTEYNAQGKLVSTENYTPLSFLKKYGDENLISNYIMLMNDPSREYYKCYEIDYDRHRYDGKNWTYVNLPVEDIKEMAIASLKDSTMMYFSCDVGKFLNSDRGLLDVKNYDYESLMGTTFGMDKKERIQSFASGSSHAMTLMAVDLDENGKPTKWMVENSWGPASGYQGHLIMTDEWFDEYMFRLVVETKYASSKIQEILKQKPIRLPAWDPMFVGEE | aminopeptidase | Y (SP) | Y | 0 | 0 | Mul. loc. | Exp. | 0 | 877 | 100 | 88.627 | 47 | Core |
| C01 | C01.UPA | UP | *B. thetaiotaomicron* |  | MKKSILIAALGLFSLSTMAQDAKPEEGFVFTTVKENPITSIKNQNRSSTCWSFSTLGFVESELLRLGKGEYDLAEMFVVHKTMQDRGANYVRYHGDSSFSPGGSFYDVMYCIKNYGIVPQEVMPGIMYGDTLPVHNELDAVASGYINAIAKGKLSKLTPVWKNGLAAIYDTYLGKCPENFTYKGKEYTPKTFAESLGLNPDDYVSLTSYTHHPFYSQFAIEIQDNWRNGLSYNLPIDEFMAVMDNAVKKGYTFAWGSDVSEQGFTRDGIAVMPDINKESELSGSDMARWTGLTTANKRQIMTTKPHPEIDVTQEMRQVAFDNWETTDDHGMVIYGIAKDQNGKEYFMVKNSWGKSGKYNGIWYASKAFVAYKTMNILVHKDALPKEIAKKLGIK | aminopeptidase | Y (SP) | Y | 0 | 0 | Per. | Exp. | 0,00E+00 | 756 | 100 | 89.594 | 35 | Shell |

Sup. Fam., Super family; Sub Fam., Sub-family; Orga., Refers to the organism from which the referenced protease is derived.; Prot. Name, Protein name; Pred. Loc., Predicted localisation ; % ID, percentage of identity; *B.*, *Bacteroides ;* Hyp. prot., Hypothetical protein; Y, Yes ; N, No ; SP, Signal peptide ; Lipo, Lipoprotein signal peptide; UP, Unassigned peptidase ; Cyto., cytoplasmic ; Cyto Memb, cytoplasmic membrane ; Extracel., extracellular ; Mul. loc., may have multiple localisation sites ; Outer Memb., outer membrane ; Per, periplasmic ; Exp., Exported ; Ic, Intracellular; M, Membrane; Cloud, Cloud genome; Core, Core genome; Shell, Shell genome.
